# Supplementary material for: Dbh+ catecholaminergic cardiomyocytes contribute to the structure and function of the cardiac conduction system in murine heart
Source: Nat Commun. 2023 Nov 28;14:7801. doi: 10.1038/s41467-023-42658-9 (PMC10684617; doi:10.1038/s41467-023-42658-9)
Supplement: Supplementary file 1 — Supplementary Information [file 41467_2023_42658_MOESM1_ESM.pdf]

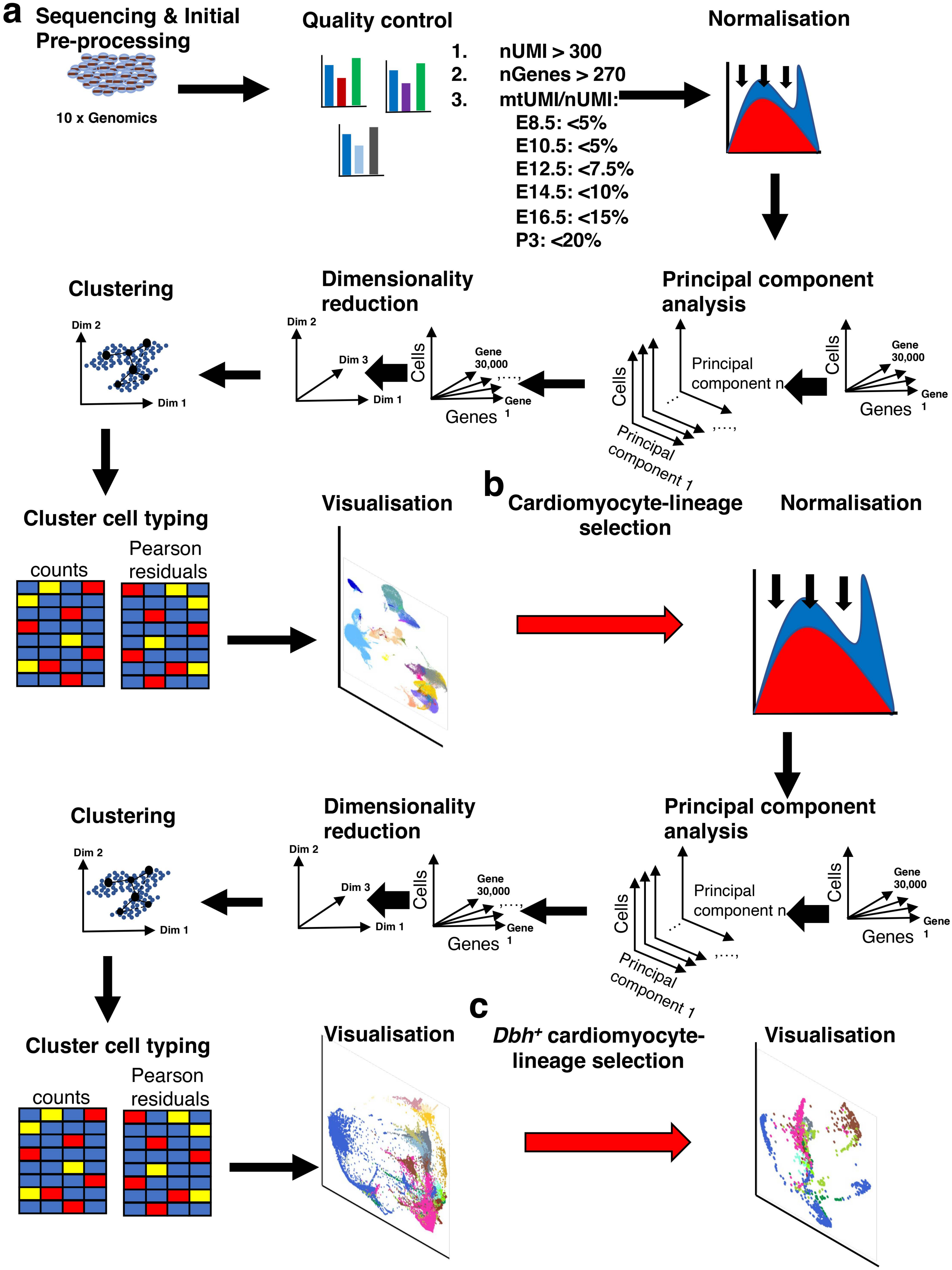
a
Sequencing & Initial Pre-processing
10 x Genomics
Quality control
1. nUMI > 300
2. nGenes > 270
3. mtUMI/nUMI:
E8.5: <5%
E10.5: <5%
E12.5: <7.5%
E14.5: <10%
E16.5: <15%
P3: <20%
Normalisation
Clustering
Dim 2
Dim 1
Dimensionality reduction
Dim 3
Cells
Gene 30,000
Gene 1
Genes
Principal component analysis
Principal component n
Principal component 1
Cluster cell typing
counts
Pearson residuals
Visualisation
b
Cardiomyocyte-lineage selection
Normalisation
Clustering
Dimensionality reduction
Principal component analysis
Cluster cell typing
counts
Pearson residuals
Visualisation
c
Dbh+ cardiomyocyte-lineage selection
Visualisation

Supplementary Figure 1. Summary of single-cell RNA sequencing computational

workflow

a i) Isolated cells were sequenced by 10 x Genomics Illumina HiSeq, and then underwent initial read-wise quality control and UMI barcode matrix construction. Cells then underwent cell-wise quality control, with cells kept only if they met all of the following characteristics described to the right. We normalised from raw RNA counts through SCTtransform, to account for variance in library size. We performed principal component analysis to identify and select only those components contributing to the majority of computational and biological variation for downstream analyses. We performed dimensionality reduction using UMAP, to reduce the dimensionality of the dataset around 10,000x, to enable more intuitive engagement. We performed Louvain clustering to identify sufficiently similar cells within our data to produce clusters. We labelled clusters with biological identities based on their gene expression, using both normalised count data and their Pearson's residuals from respective average gene expression. Any ambiguous clusters were analysed further, including looking at wider gene expression, subclustering, and in a small number of cases assigning biological identities directly to cells. viii) We visualised the data in its completed format as presented in the paper.

b We selected only those cell types involved in the cardiomyocyte lineage, from mesoderm through to P3 cardiomyocytes (See methods), and subset their raw count data from the original raw matrix. We normalised from raw RNA counts through SCTtransform, to account for variance in library size. We performed principal component analysis to identify and select only those components contributing to the majority of computational and biological variation for downstream analyses. We performed dimensionality reduction using PHATE, to reduce the dimensionality of the dataset around 10,000x, to enable more intuitive engagement. We performed kmeans clustering on the PHATE operator, similar to spectral clustering, to identify sufficiently similar cells within our data to produce clusters. We labelled clusters with biological identities based on their gene expression, using both normalised count data and their Pearson's residuals from respective average gene expression. Any ambiguous clusters were analysed further, including looking at wider gene expression. We visualised the data in its completed format as presented in the paper.

c We selected all cells from the cardiomyocyte lineage that had raw UMI counts of Dbh>0. We visualised the data in its completed format as presented in the paper.

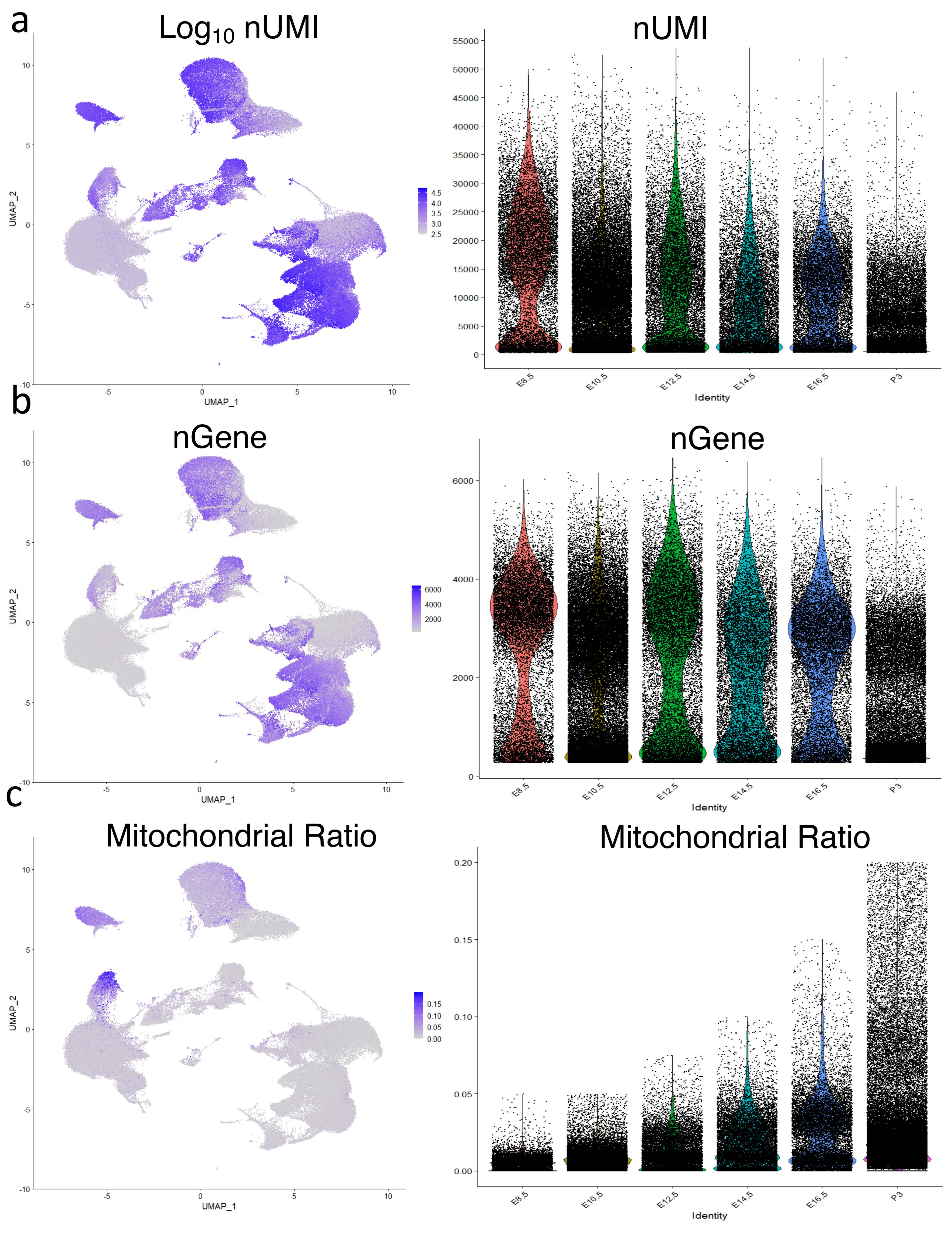
a
Log10 nUMI
nUMI
b
nGene
nGene
c
Mitochondrial Ratio
Mitochondrial Ratio
UMAP_1
UMAP_2
Identity
E8.5
E10.5
E12.5
E14.5
E16.5
P3

Supplementary Figure 2. Quality control metrics for single-cell data.
a. A UMAP plot of all initial cells post-quality control, coloured by Log_{10}(nUMI) and a violin plot demonstrating the same across different stages.
b. A UMAP plot of all initial cells post-quality control, coloured by nGene and a violin plot demonstrating the same across different stages.
c. A UMAP plot of all initial cells post-quality control, coloured by the ratio of mitochondrial nUMI to total nUMI and a violin plot demonstrating the same ac
ross different stages.

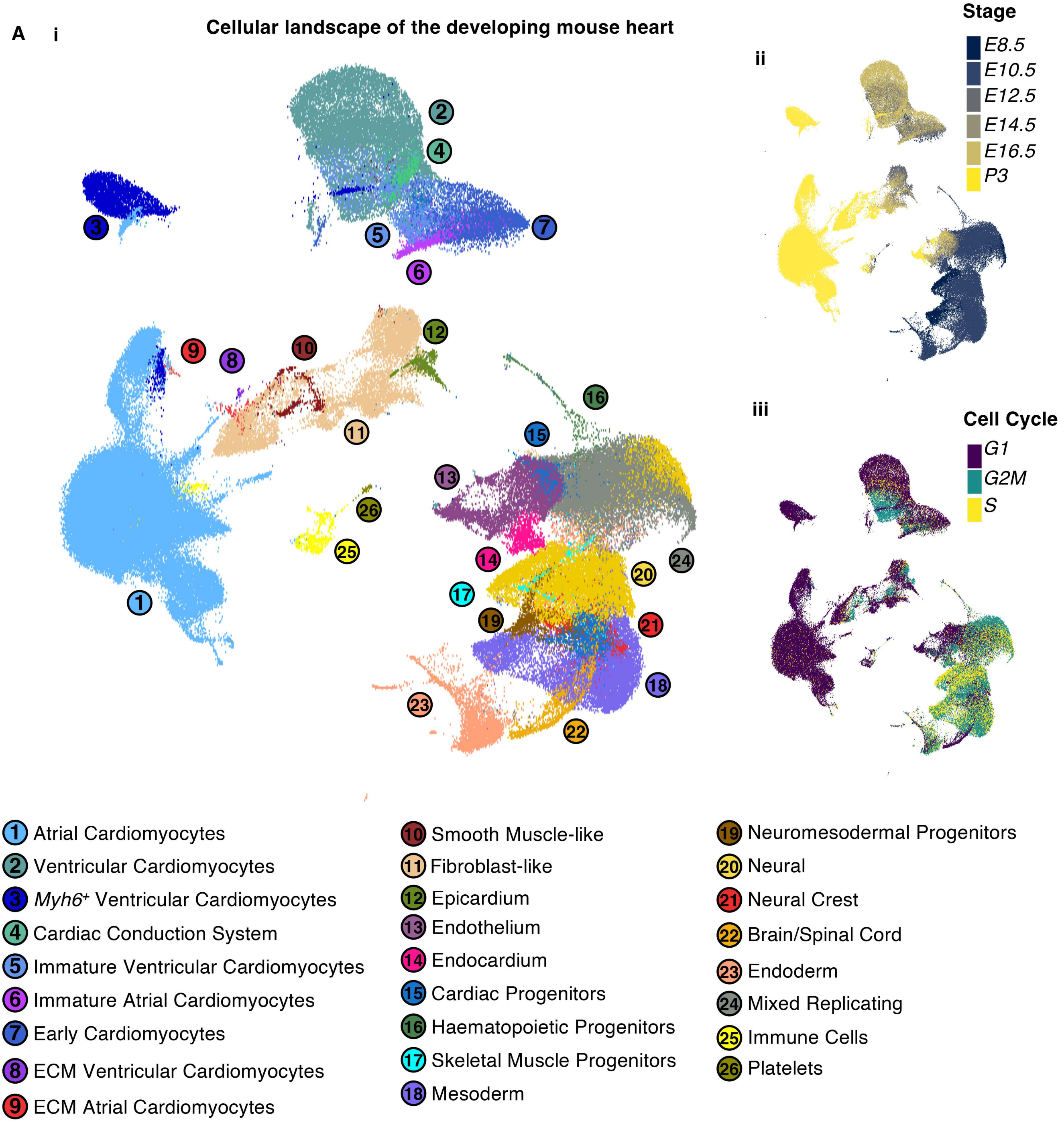
A
i
Cellular landscape of the developing mouse heart
ii
Stage
E8.5
E10.5
E12.5
E14.5
E16.5
P3
iii
Cell Cycle
G1
G2M
S
1 Atrial Cardiomyocytes
2 Ventricular Cardiomyocytes
3 Myh6+ Ventricular Cardiomyocytes
4 Cardiac Conduction System
5 Immature Ventricular Cardiomyocytes
6 Immature Atrial Cardiomyocytes
7 Early Cardiomyocytes
8 ECM Ventricular Cardiomyocytes
9 ECM Atrial Cardiomyocytes
10 Smooth Muscle-like
11 Fibroblast-like
12 Epicardium
13 Endothelium
14 Endocardium
15 Cardiac Progenitors
16 Haematopoietic Progenitors
17 Skeletal Muscle Progenitors
18 Mesoderm
19 Neuromesodermal Progenitors
20 Neural
21 Neural Crest
22 Brain/Spinal Cord
23 Endoderm
24 Mixed Replicating
25 Immune Cells
26 Platelets

Supplementary Figure 3. scRNAseq identified 4 major groups of cell types across the developing mouse heart.

a) i) UMAP plot of all initial cells post-quality control, coloured by cell type identified after unsupervised clustering. This dataset includes E8.5 and E10.5 whole embryos, and whole isolated hearts from E12.5, E14.5, E16.5, and P3 mice. Corresponding cell types are labelled below.

ii) UMAP plot of all initial cells post-quality control, coloured by stage of tissue isolation.

iii) UMAP plot of all initial cells post-quality control, coloured by predicted Cell Cycle stage identified using Seurat.

Mixed Replicating
Snrpg^{hi}
Rp-genehi
Ptma^{hi}
Other

Mesoderm
Foxd1^{+}
Prrx1^{+}
Meox1^{+}

Neural
Sox2^{+}
Otx2^{+}
Pax6^{+}

Endoderm
Pyy^{+}
Epcam^{+}
Afp^{+}
Ttr^{+}

Cardiac Progenitors
Mesoderm Genes^{+}
Hand1^{hi}
Wnt2^{hi}
Bmp4^{+}

Haematopoietic Progenitors
Redrum^{+}
Hemgn^{+}
Hbb-genes^{+}

Skeletal Muscle Progenitors
Mesoderm Genes^{+}
Myf6^{+}
Myog^{+}

Neuromesodermal Progenitors
Neural Genes^{+}
Mesoderm Genes^{+}
Sox2^{+}
T^{hi}

Neural Crest
Neural Genes^{+}
Sox10^{+}
Foxd3^{+}

Early Cardiomyocytes
Tnni1^{hi}
Smpx^{hi}
Tnni3^{lo}
Myl7^{+}
Tagln^{+}
Pln^{+}
Atp2a2^{lo}

Endocardium
Endothelium Genes^{+}
Etv2^{hi}
Tal1^{+}

Endothelium
Ecscr^{hi}
Emcn^{hi}
Cdh5^{hi}
Kdr^{hi}

Epicardium
Upk3b^{hi}
Upk1b^{hi}
Aldh1a2^{hi}

Smooth Muscle-like
Rgs5^{hi}
Myh11^{hi}
Lmod1^{hi}

Fibroblast-like
Fbln2^{hi}
Fbn1^{hi}
Postn^{hi}
Col1a1^{hi}

Brain/Spinal Cord
Neural Genes^{+}
Isl1^{+}
Neurog1^{+}
Onecut2^{+}

Early Ventricular Cardiomyocytes
Early Cardiomyocyte Genes^{+}
Myl7^{lo}
Myl2^{hi}
Myh7^{+}
Pln^{+}

Early Atrial Cardiomyocytes
Early Cardiomyocyte Genes^{+}
Myl7^{hi}
Myl2^{lo}
Sln^{+}

Platelets
Ppbp^{hi}
Pf4^{hi}
Gp1bb^{hi}
Gp6^{hi}

Ventricular Cardiomyocytes
Atrial Genes$^{lo/-}$
Myl2^{hi}
Myh7^{hi}
Kcne1^{hi}
Gja1^{hi}

Cardiac Conduction System
Hcn4^{+}
Cacna2d2^{hi}
Tbx3^{+}
Shox2^{+}
Cacna1g^{+}

Atrial Cardiomyocytes
Ventricular Genes$^{lo/-}$
Myl7^{hi}
Nppa^{hi}
Sln^{hi}
Myh6^{+}

Immune Cells
C1qb^{hi}
Tyrobp^{hi}
C1qc^{hi}
Fcer1g^{hi}
Lyz2^{hi}
Fcgr3^{hi}

ECM Ventricular Cardiomyocytes
Ventricular Cardiomyocyte Genes^{+}
Fbn1^{+}
Postn^{+}
Col1a1^{+}

***Myh6*$^{+}$ Ventricular Cardiomyocytes**
Ventricular Genes^{hi}
Atrial Genes^{lo}
Myh6^{hi}

ECM Atrial Cardiomyocytes
Atrial Cardiomyocyte Genes^{+}
Fbn1^{+}
Postn^{+}
Col1a1^{+}

Supplementary Figure 4. Key marker genes for each population initially identified within our scRNA-Seq dataset.

Each cell type corresponding to Supplementary Figure 3 cell types, is identified in bold, with some respective key exemplary key markers underneath. Gene$_{+}$ indicates a gene is expressed above the population level in this group. Gene$_{hi}$ indicates that a gene is highly expressed above the population level in this group. Gene$_{lo}$ indicates a gene is expressed below the level expected for it to alter the classification of a particular group and is typically in reference to sub-population expression levels rather than the whole dataset.

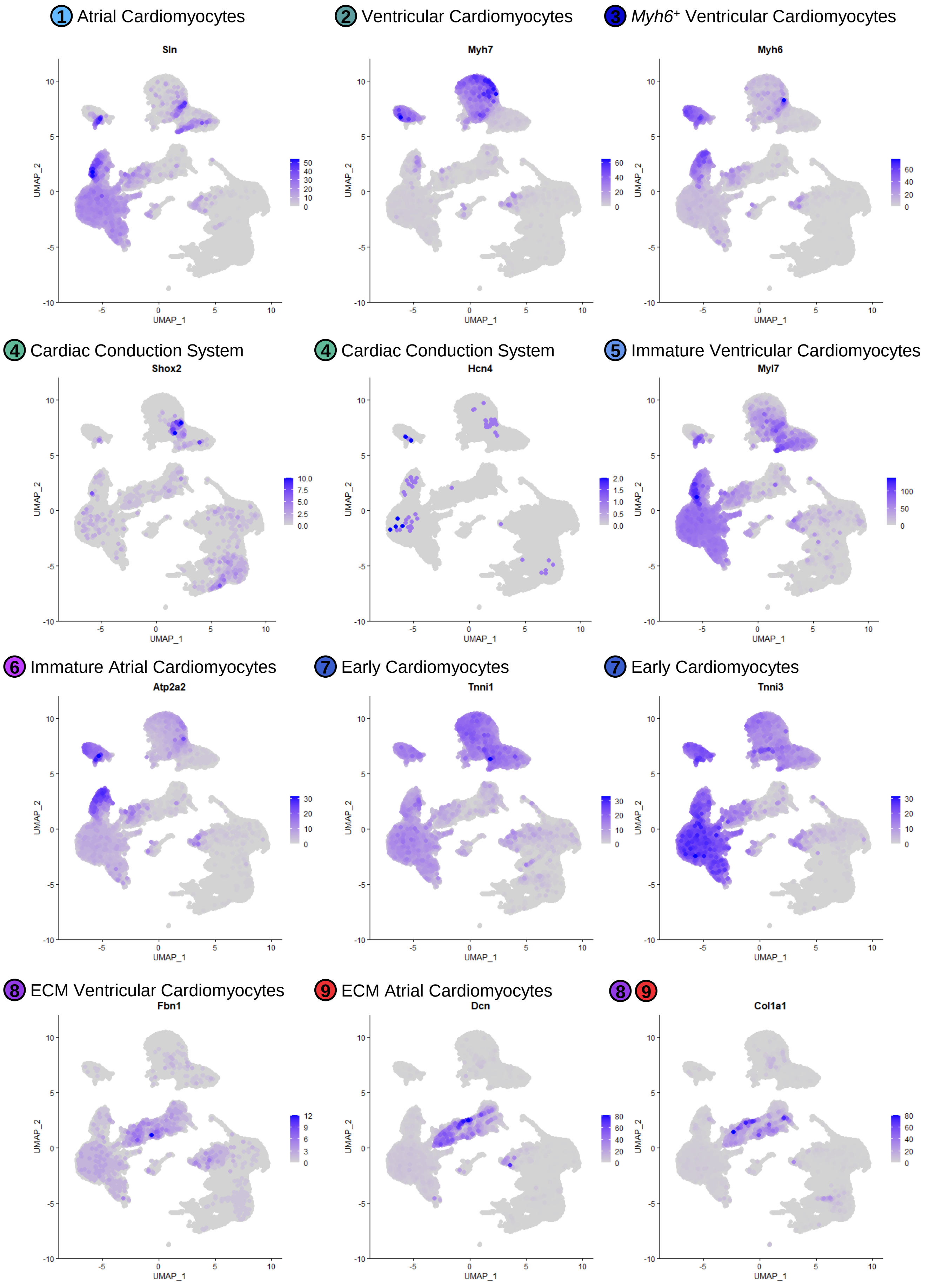

1 Atrial Cardiomyocytes
Sln
2 Ventricular Cardiomyocytes
Myh7
3 *Myh6*+ Ventricular Cardiomyocytes
Myh6
4 Cardiac Conduction System
Shox2
4 Cardiac Conduction System
Hcn4
5 Immature Ventricular Cardiomyocytes
Myl7
6 Immature Atrial Cardiomyocytes
Atp2a2
7 Early Cardiomyocytes
Tnni1
7 Early Cardiomyocytes
Tnni3
8 ECM Ventricular Cardiomyocytes
Fbn1
9 ECM Atrial Cardiomyocytes
Dcn
8 9
Col1a1
UMAP_1
UMAP_2

Supplementary Figure 5a. **Expression of example marker genes across different cell populations identified in whole dataset.**
Each panel displays a UMAP plot of the whole dataset with cells coloured by the expression of the specific gene identified at the top of the plot. The expression is given in post-SCT normalized counts. The cell type that the individual genes are representative of are indicated above each plot, with corresponding colours and numbers as in Supplementary Figure 3.

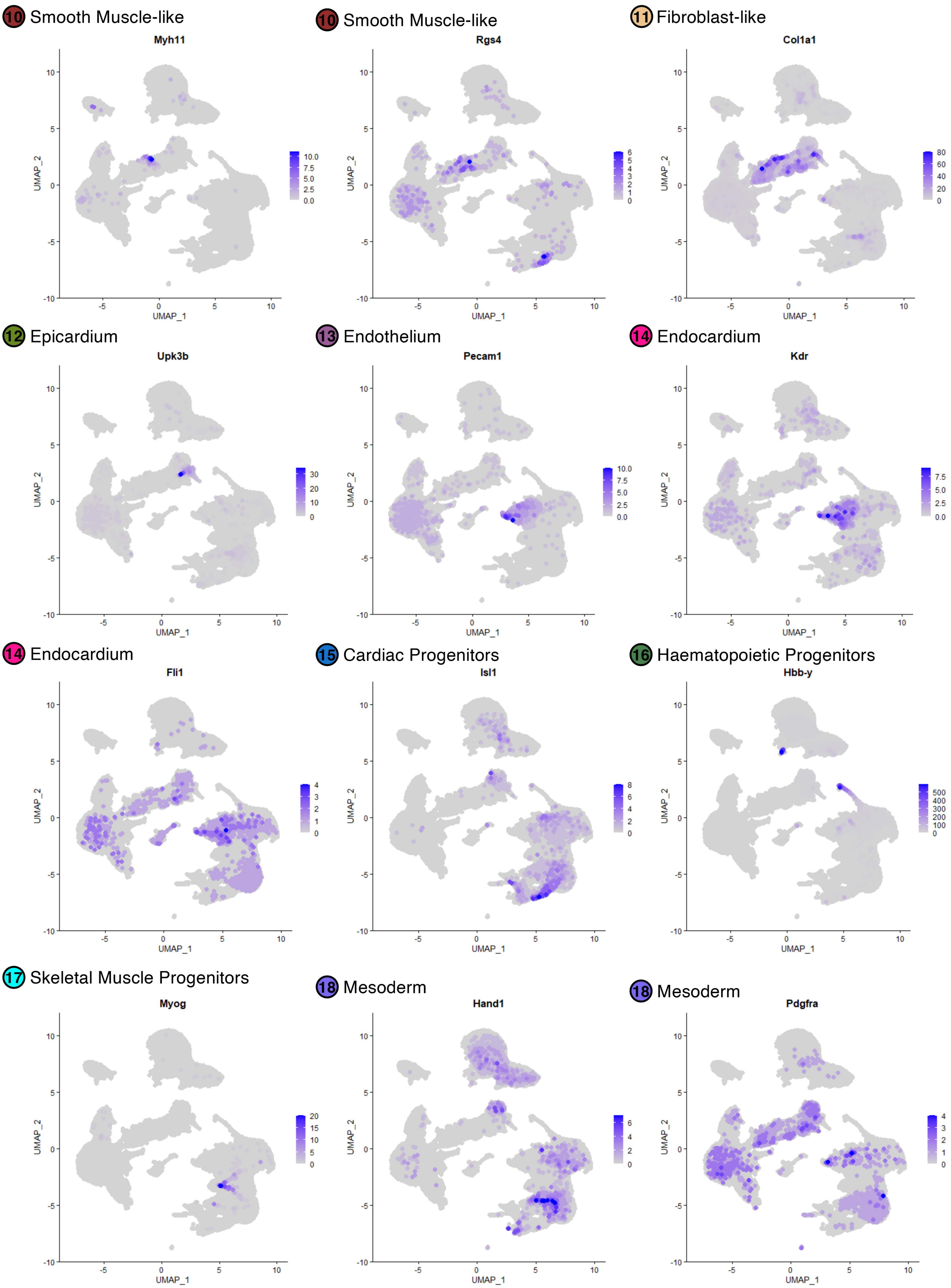
10 Smooth Muscle-like
Myh11
10 Smooth Muscle-like
Rgs4
11 Fibroblast-like
Col1a1
12 Epicardium
Upk3b
13 Endothelium
Pecam1
14 Endocardium
Kdr
14 Endocardium
Fli1
15 Cardiac Progenitors
Isl1
16 Haematopoietic Progenitors
Hbb-y
17 Skeletal Muscle Progenitors
Myog
18 Mesoderm
Hand1
18 Mesoderm
Pdgfra
UMAP_1
UMAP_2

Supplementary Figure 5b. Expression of example marker genes across different cell populations identified in whole dataset.

Each panel displays a UMAP plot of the whole dataset with cells coloured by the expression of the specific gene identified at the top of the plot. The expression is given in post-SCT normalized counts. The cell type that the individual genes are representative of are indicated above each plot, with corresponding colours and numbers as in Supplementary Figure 3.

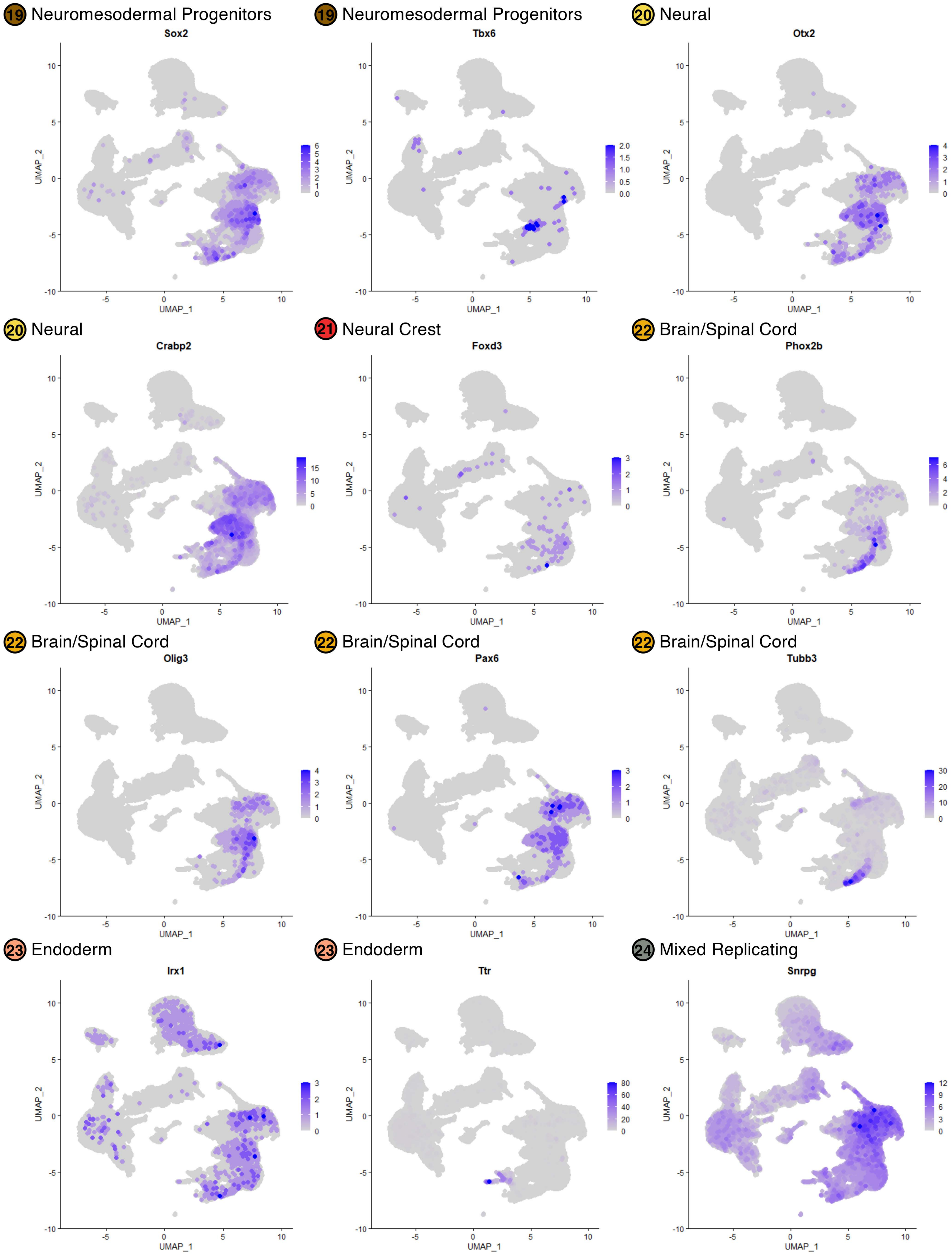
19 Neuromesodermal Progenitors
Sox2
19 Neuromesodermal Progenitors
Tbx6
20 Neural
Otx2
20 Neural
Crabp2
21 Neural Crest
Foxd3
22 Brain/Spinal Cord
Phox2b
22 Brain/Spinal Cord
Olig3
22 Brain/Spinal Cord
Pax6
22 Brain/Spinal Cord
Tubb3
23 Endoderm
Irx1
23 Endoderm
Ttr
24 Mixed Replicating
Snrpg
UMAP_1
UMAP_2

Supplementary Figure 5c. Expression of example marker genes across different cell populations identified in whole dataset.

Each panel displays a UMAP plot of the whole dataset with cells coloured by the expression of the specific gene identified at the top of the plot. The expression is given in post-SCT normalized counts. The cell type that the individual genes are representative of are indicated above each plot, with corresponding colours and numbers as in Supplementary Figure 3.

25 Immune Cells

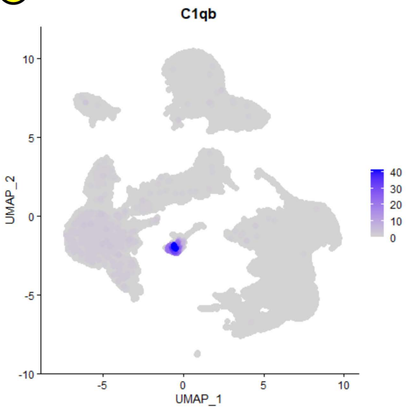

26 Platelets

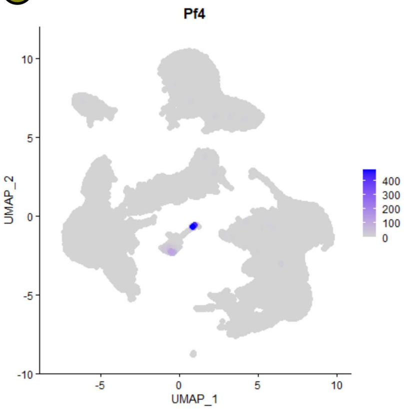

Supplementary Figure 5d. Expression of example marker genes across different cell populations identified in whole dataset.
Each panel displays a UMAP plot of the whole dataset with cells coloured by the expression of the specific gene identified at the top of the plot. The expression is given in post-SCT normalized counts. The cell type that the individual genes are representative of are indicated above each plot, with corresponding colours and numbers as in Supplementary Figure 3.

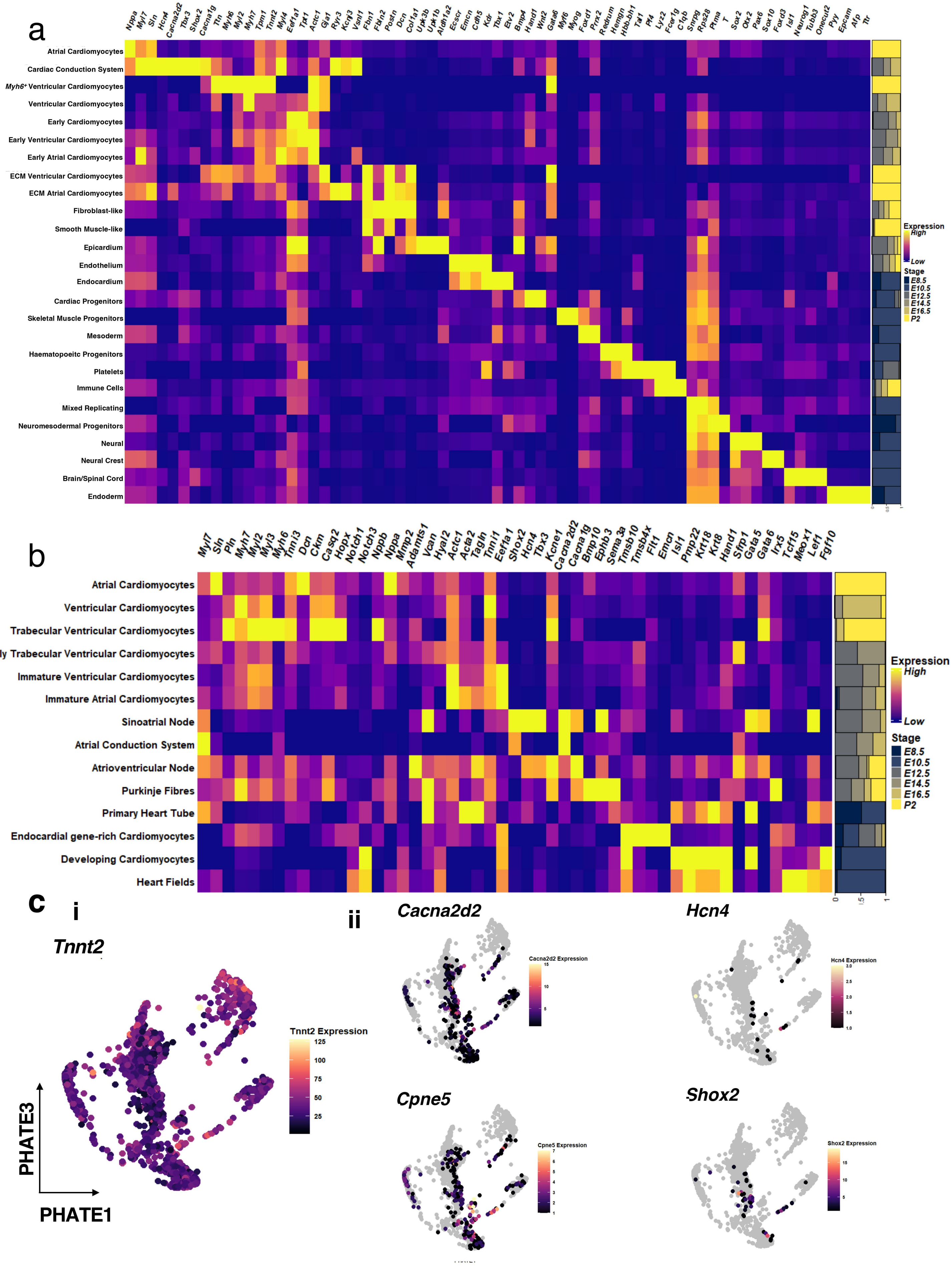
a
Nppa Myl7 Sln Hcn4 Cacna2d2 Tbx3 Shox2 Cacna1g Ttn Myh6 Myl2 Myh7 Tpm1 Tnnt2 Myl4 Eef1a1 Tpt1 Actc1 Gja1 Ryr3 Kcnj3 Vsnl1 Fbn1 Fbln2 Postn Dcn Col1a1 Upk3b Upk1b Aldh1a2 Ecscr Emcn Cdh5 Kdr Tbx1 Etv2 Bmp4 Hand1 Wnt2 Gata6 Myf6 Myog Foxd1 Prrx2 Redrum Hemgn Hbb-bh1 Tal1 Pf4 Lyz2 Fcer1g C1qb Snrpg Rps28 Ptma T Sox2 Otx2 Pax6 Sox10 Foxd3 Isl1 Neurog1 Tubb3 Onecut2 Pyy Epcam Afp Ttr
Atrial Cardiomyocytes
Cardiac Conduction System
Myh6+ Ventricular Cardiomyocytes
Ventricular Cardiomyocytes
Early Cardiomyocytes
Early Ventricular Cardiomyocytes
Early Atrial Cardiomyocytes
ECM Ventricular Cardiomyocytes
ECM Atrial Cardiomyocytes
Fibroblast-like
Smooth Muscle-like
Epicardium
Endothelium
Endocardium
Cardiac Progenitors
Skeletal Muscle Progenitors
Mesoderm
Haematopoeitc Progenitors
Platelets
Immune Cells
Mixed Replicating
Neuromesodermal Progenitors
Neural
Neural Crest
Brain/Spinal Cord
Endoderm
Expression
High
Low
Stage
E8.5
E10.5
E12.5
E14.5
E16.5
P2
b
Myl7 Sln Pln Myh7 Myl2 Myl3 Myh6 Tnni3 Dcn Ckm Casq2 Hopx Notch1 Notch3 Nppb Nppa Mmp2 Adamts1 Vcan Hyal2 Actc1 Acta2 Tagln Tnni1 Eef1a1 Shox2 Hcn4 Tbx3 Kcne1 Cacna2d2 Cacna1g Bmp10 Ephb3 Sema3a Tmsb10 Tmsb4x Flt1 Emcn Isl1 Pmp22 Krt18 Krt8 Hand1 Sfrp1 Gata5 Gata6 Irx5 Tcf15 Meox1 Lef1 Fgf10
Atrial Cardiomyocytes
Ventricular Cardiomyocytes
Trabecular Ventricular Cardiomyocytes
arly Trabecular Ventricular Cardiomyocytes
Immature Ventricular Cardiomyocytes
Immature Atrial Cardiomyocytes
Sinoatrial Node
Atrial Conduction System
Atrioventricular Node
Purkinje Fibres
Primary Heart Tube
Endocardial gene-rich Cardiomyocytes
Developing Cardiomyocytes
Heart Fields
Expression
High
Low
Stage
E8.5
E10.5
E12.5
E14.5
E16.5
P2
0
.5
1
c
i
Tnnt2
Tnnt2 Expression
125
100
75
50
25
PHATE3
PHATE1
ii
Cacna2d2
Cacna2d2 Expression
15
10
5
Hcn4
Hcn4 Expression
3.0
2.5
2.0
1.5
1.0
Cpne5
Cpne5 Expression
7
6
5
4
3
2
1
Shox2
Shox2 Expression
15
10
5

Supplementary Figure 6. Cell types display a range of associated biologically defining marker genes in our single-cell RNA sequencing analyses.

a) Cell types identified in Supplementary Figure 3 from initial analysis of all cells post-quality control. The annotation on the right describes the relative proportions of each cluster in terms of developmental stage. Values for expression are normalised by row and then by column for visualization purposes.

b) Cell types identified in Figure 1b from cardiomyocyte lineage. The annotation on the right describes the relative proportions of each cluster in terms of developmental stage. Values for expression are normalised by row and then by column for visualisation purposes.

c) i) A 2D plot of *Tnnt2* expression across *Dbh*$_+$ cardiomyocytes. ii) 2D plots of various CCS markers across *Dbh*$_+$ cardiomyocytes.

Heart Fields

Osr1^{hi}
Hand1^{hi}
Wnt2^{hi}
Krt8^{+}

Miscellaneous

Markers for other cell types$^{-/lo}$
Cell damage markers^{hi}
E.g. *Dnajb3*^{hi}

Developing Cardiomyocytes

Acta2^{hi}
Hmga2^{hi}
Pmp22^{hi}
Krt8/18^{hi}
Isl1^{hi}
Notch3^{hi}

Endocardial Gene-rich Cardiomyocytes

Myl7^{+}
Myl2^{+}
Tnni1^{+}
Tmsb4x^{hi}
Flt1^{hi}
Emcn^{hi}

Primary Heart Tube

Developing Cardiomyocyte Genes^{+}
Isl1^{lo}
Notch3^{lo}
Acta2^{+}
Tagln^{+}

Immature Atrial Cardiomyocytes

Atrial Cardiomyocyte Genes^{lo}
Tnni3^{lo}
Tnni1^{hi}
Acta2^{hi}

Immature Ventricular Cardiomyocytes

Ventricular Cardiomyocyte Genes^{lo}
Tnni3^{lo}
Tnni1^{hi}
Acta2^{hi}

Early Trabecular Ventricular Cardiomyocytes

Ventricular Cardiomyocyte Genes^{+}
Nppb^{+}
Nppa^{+}
Vcan^{hi}
Hyal2^{hi}
Tnni1^{hi}

Atrial Cardiomyocytes

Myl7^{hi}
Myh6^{hi}
Myl4^{hi}
Nppa^{hi}
Sln^{hi}
Tnni3^{hi}

Ventricular Cardiomyocytes

Myh7^{hi}
Myl2^{hi}
Pln^{hi}
Tnni3^{hi}

Trabecular Ventricular Cardiomyocytes

Ventricular Cardiomyocyte Genes^{+}
Myh6^{hi}
Nppb^{hi}
Hopx^{hi}
Nppa^{+}
Casq2^{hi}

Atrial Cardiac Conduction System

Myl7^{+}
Tnnt2^{+}
Cacna2d2^{hi}
Igfbp5^{hi}
Nppa^{lo}

Sinoatrial Node

Myl7^{+}
Tnnt2^{+}
Cacna2d2^{hi}
Shox2^{hi}
Igfbp5^{hi}
Vsnl1^{+}
Hcn4^{+}

Atrioventricular Node

Myl7^{+}
Tnnt2^{+}
Rspo3^{hi}
Cacna2d2^{hi}
Cacna1g^{hi}
Bmp2^{hi}
Igfbp5^{hi}
Shox2^{-}
Vsnl1^{-}

Purkinje Fibres

Trabecular Ventricular Cardiomyocyte Genes^{+}
Nppa^{lo}
Nppb^{lo}
Igfbp5^{hi}
Slit2^{hi}
Sema3a^{hi}
Cacna1g^{hi}
Ephb3^{hi}

Supplementary Figure 7. Key marker genes for each population initially identified within our scRNA-Seq dataset cardiomyocyte lineage.

Each cell type corresponding to Supplementary Figure 3 cell types, is identified in bold, with some respective key exemplary key markers underneath. $Gene_{+}$ indicates a gene is expressed above the population level in this group. $Gene_{hi}$ indicates that a gene is highly expressed above the population level in this group. $Gene_{lo}$ indicates a gene is expressed below the level expected for it to alter the classification of a particular group and is typically in reference to sub-population expression levels rather than the whole dataset.

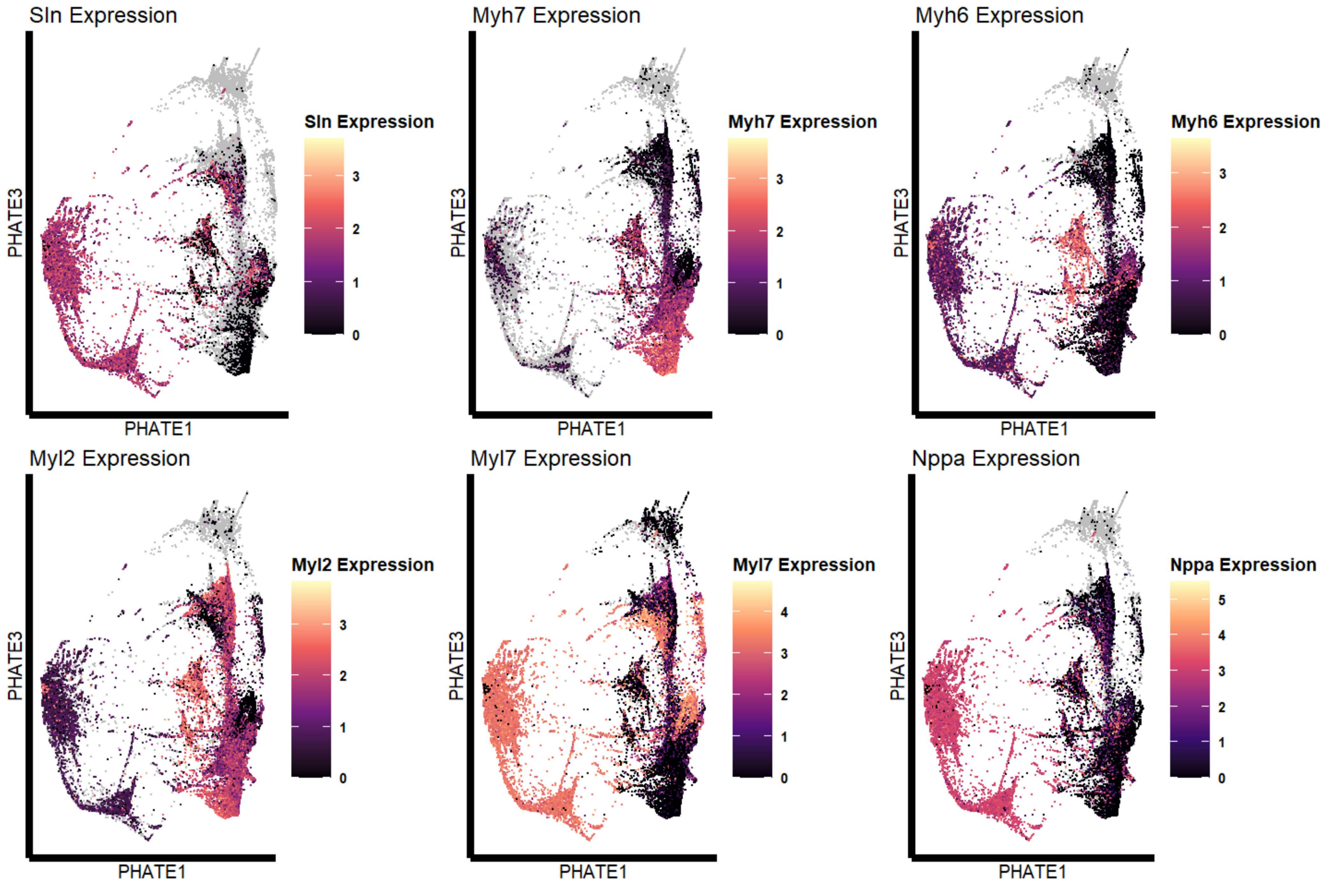

Sln Expression
Sln Expression
3
2
1
0
PHATE3
PHATE1
Myh7 Expression
Myh7 Expression
3
2
1
0
PHATE3
PHATE1
Myh6 Expression
Myh6 Expression
3
2
1
0
PHATE3
PHATE1
Myl2 Expression
Myl2 Expression
3
2
1
0
PHATE3
PHATE1
Myl7 Expression
Myl7 Expression
4
3
2
1
0
PHATE3
PHATE1
Nppa Expression
Nppa Expression
5
4
3
2
1
0
PHATE3
PHATE1

Supplementary Figure 8a. Expression of example marker genes across different cell populations identified within the cardiomyocyte lineage.
Each panel displays a PHATE plot of the cardiomyocyte lineage with cells coloured by the expression of the specific gene identified at the top of the plot. The expression is given in log counts.

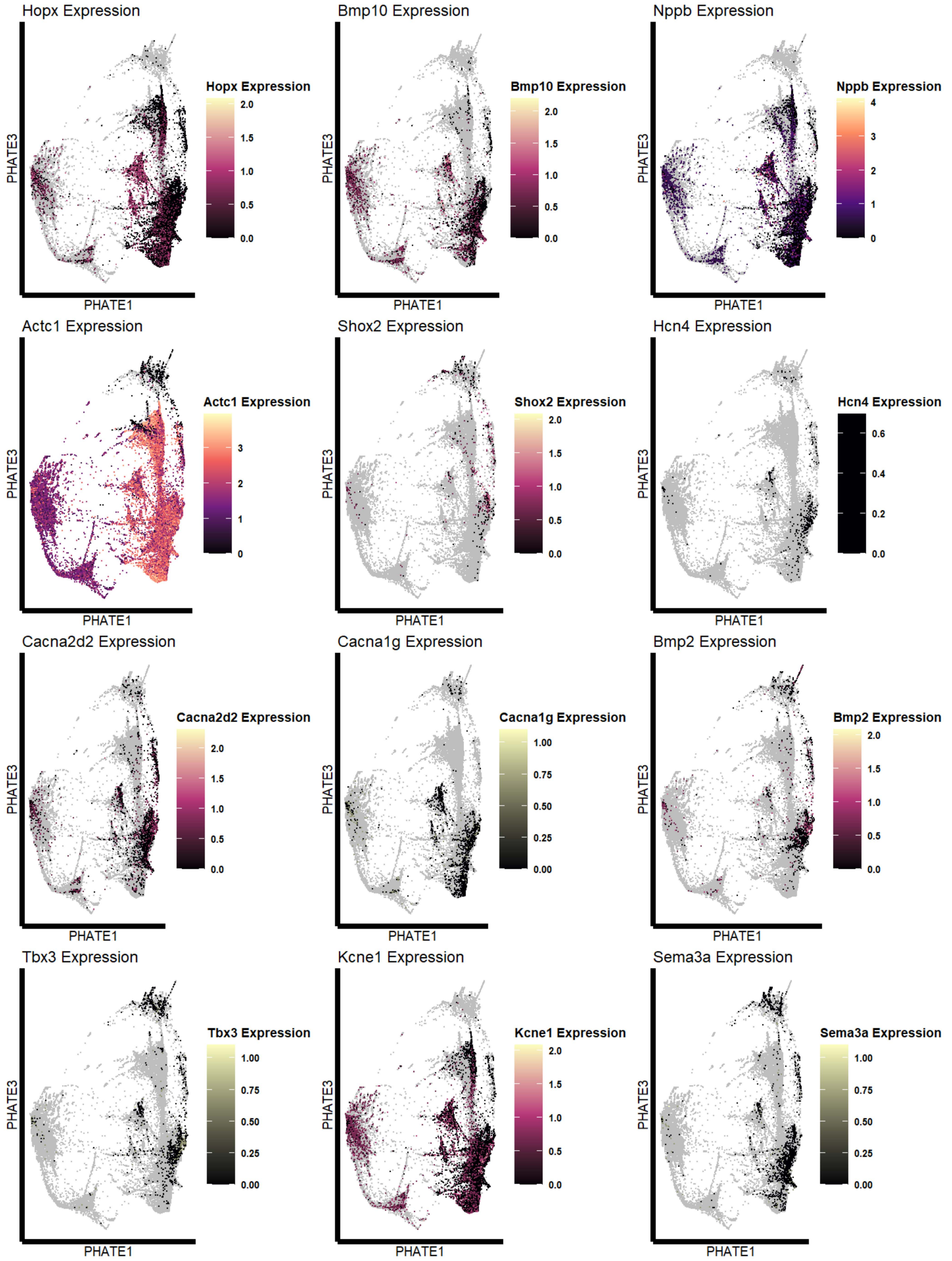

Hopx Expression
Hopx Expression
2.0
1.5
1.0
0.5
0.0
PHATE3
PHATE1
Bmp10 Expression
Bmp10 Expression
2.0
1.5
1.0
0.5
0.0
PHATE3
PHATE1
Nppb Expression
Nppb Expression
4
3
2
1
0
PHATE3
PHATE1
Actc1 Expression
Actc1 Expression
3
2
1
0
PHATE3
PHATE1
Shox2 Expression
Shox2 Expression
2.0
1.5
1.0
0.5
0.0
PHATE3
PHATE1
Hcn4 Expression
Hcn4 Expression
0.6
0.4
0.2
0.0
PHATE3
PHATE1
Cacna2d2 Expression
Cacna2d2 Expression
2.0
1.5
1.0
0.5
0.0
PHATE3
PHATE1
Cacna1g Expression
Cacna1g Expression
1.00
0.75
0.50
0.25
0.00
PHATE3
PHATE1
Bmp2 Expression
Bmp2 Expression
2.0
1.5
1.0
0.5
0.0
PHATE3
PHATE1
Tbx3 Expression
Tbx3 Expression
1.00
0.75
0.50
0.25
0.00
PHATE3
PHATE1
Kcne1 Expression
Kcne1 Expression
2.0
1.5
1.0
0.5
0.0
PHATE3
PHATE1
Sema3a Expression
Sema3a Expression
1.00
0.75
0.50
0.25
0.00
PHATE3
PHATE1

Supplementary Figure 8b. **Expression of example marker genes across different cell populations identified within the cardiomyocyte lineage.**

Each panel displays a PHATE plot of the cardiomyocyte lineage with cells coloured by the expression of the specific gene identified at the top of the plot. The expression is given in log counts.

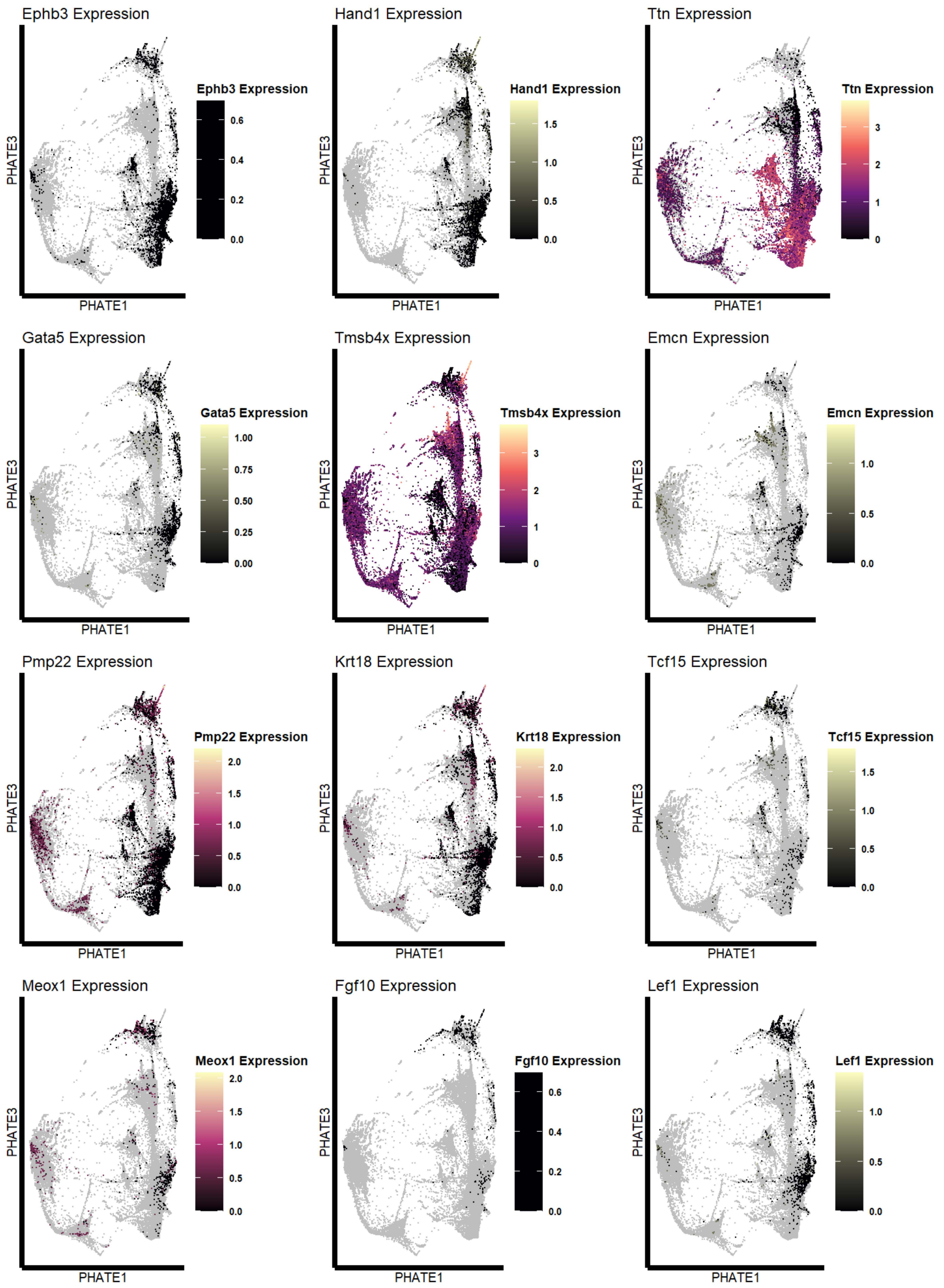

Ephb3 Expression
Hand1 Expression
Ttn Expression
Gata5 Expression
Tmsb4x Expression
Emcn Expression
Pmp22 Expression
Krt18 Expression
Tcf15 Expression
Meox1 Expression
Fgf10 Expression
Lef1 Expression
PHATE1
PHATE3

Supplementary Figure 8c. **Expression of example marker genes across different cell populations identified within the cardiomyocyte lineage.**
Each panel displays a PHATE plot of the cardiomyocyte lineage with cells coloured by the expression of the specific gene identified at the top of the plot. The expression is given in log counts.

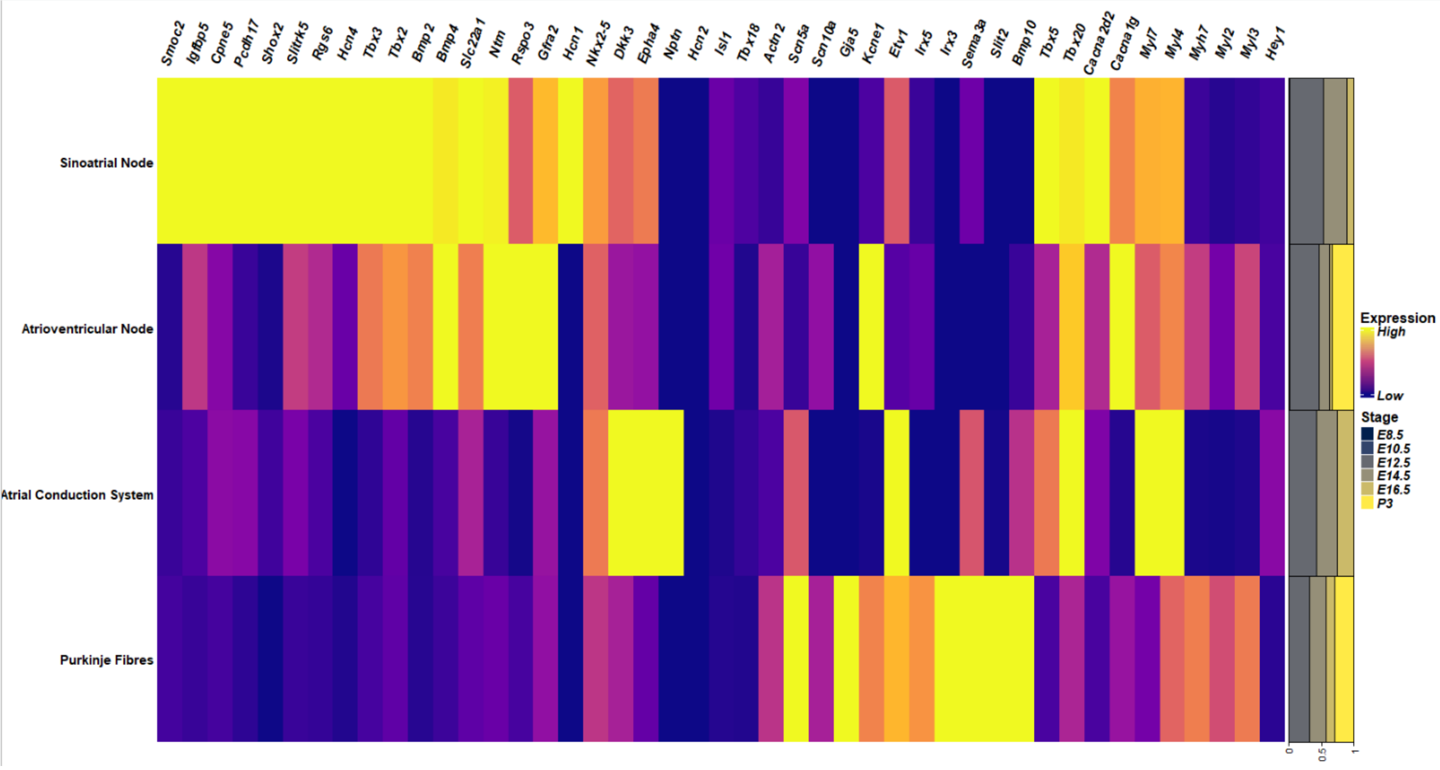

Smoc2
Igfbp5
Cpne5
Pcdh17
Shox2
Slitrk5
Rgs6
Hcn4
Tbx3
Tbx2
Bmp2
Bmp4
Slc22a1
Ntm
Rspo3
Gfra2
Hcn1
Nkx2-5
Dkk3
Epha4
Nptn
Hcn2
Isl1
Tbx18
Actn2
Scn5a
Scn10a
Gja5
Kcne1
Etv1
Irx5
Irx3
Sema3a
Slit2
Bmp10
Tbx5
Tbx20
Cacna2d2
Cacna1g
Myl7
Myl4
Myh7
Myl2
Myl3
Hey1
Sinoatrial Node
Atrioventricular Node
Atrial Conduction System
Purkinje Fibres
Expression
High
Low
Stage
E8.5
E10.5
E12.5
E14.5
E16.5
P3
0
0.5
1

Supplementary Figure 9. We can resolve several distinct CCS-associated populations within our cardiomyocyte lineage.

A heatmap displaying cell types identified in Figure 1b from cardiomyocyte lineage with the expression of a range of CCS-associated marker genes. The annotation on the right describes the relative proportions of each cluster in terms of developmental stage. Values for expression are normalised by row and then by column for visualisation purposes.

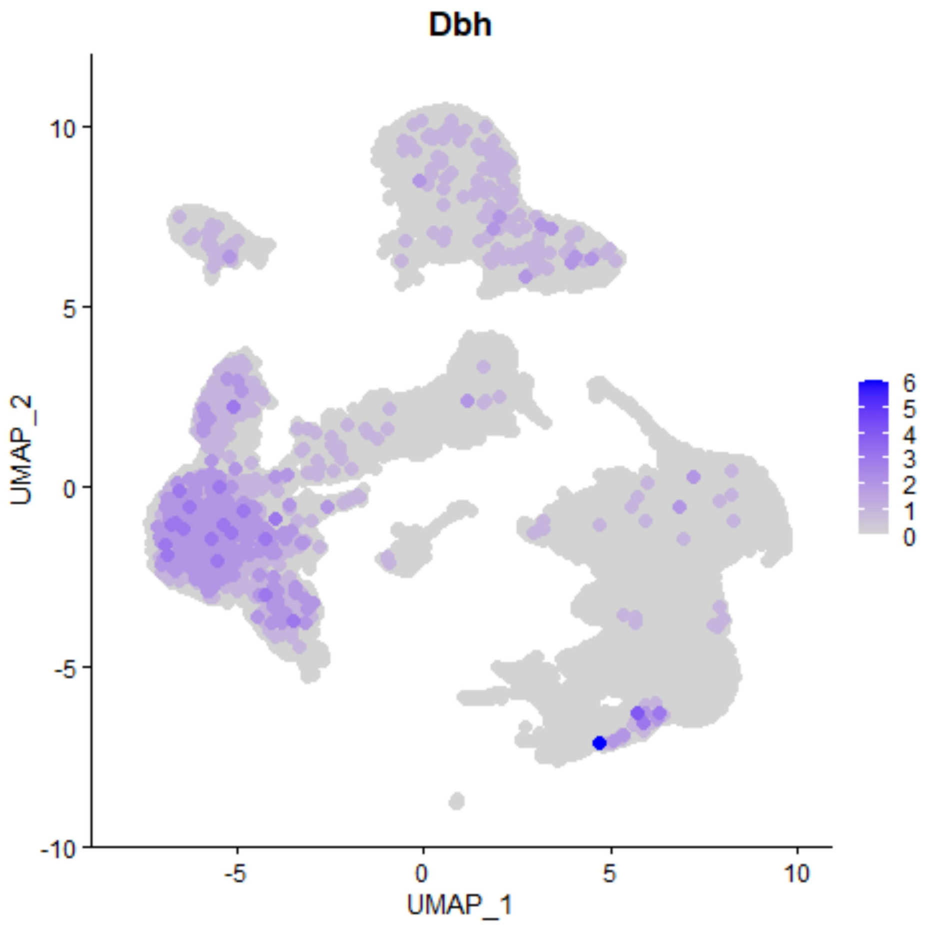
Dbh
UMAP_2
UMAP_1
10
5
0
-5
-10
-5
0
5
10
6
5
4
3
2
1
0

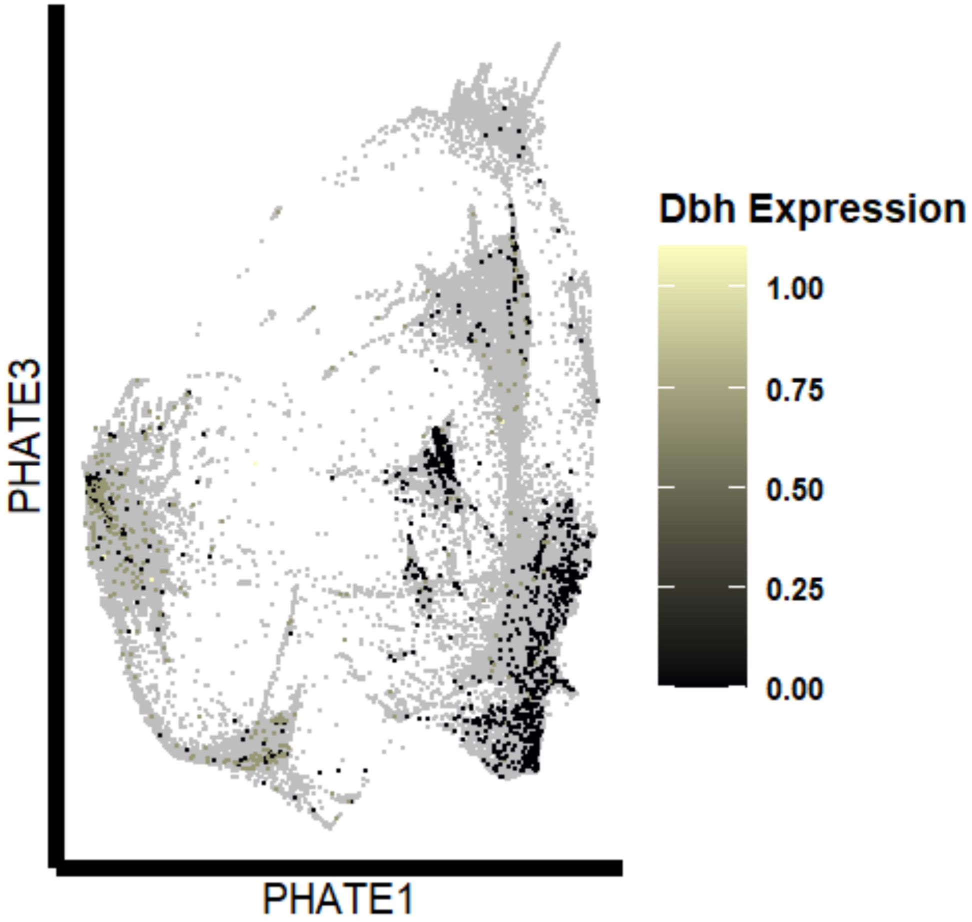
Dbh Expression
1.00
0.75
0.50
0.25
0.00
PHATE3
PHATE1

Supplementary Figure 10. Expression of *Dbh* across the whole dataset and across the cardiomyocyte lineage

The top panel displays the expression of Dbh across the whole dataset UMAP plot in post-SCT normalized counts. Dbh expression can be observed primarily across cardiomyocytes and the developing brainstem/brain. The bottom panel displays Dbh expression across the ardiomyocyte lineage PHATE plot in log counts.

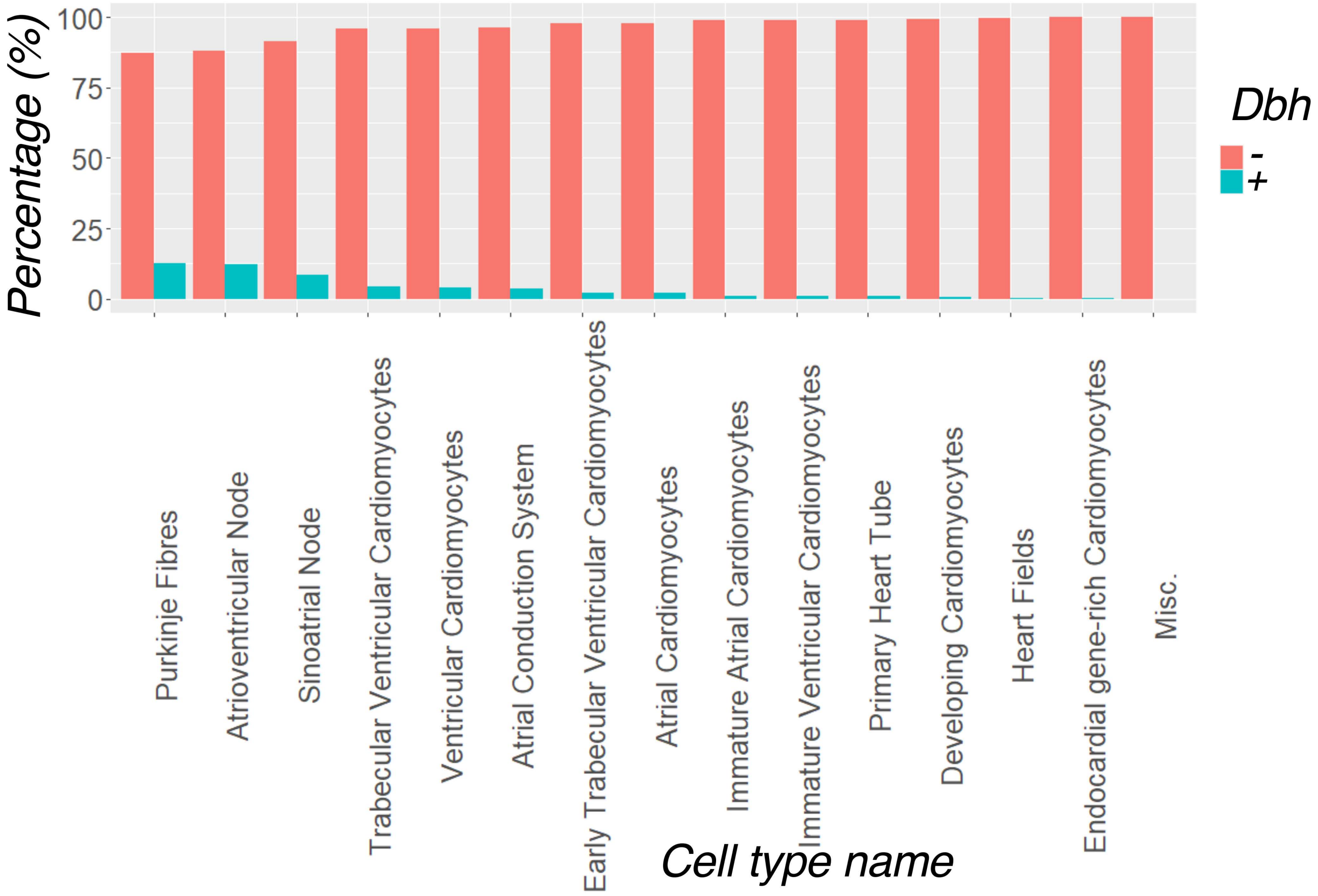

Cell type	Total number of cells in type (N)	Number of cells *Dbh+* (N)	Number of cells *Dbh-* (N)	Percentage *Dbh+* (%)
Atrial Cardiomyocytes	74021	1575	72446	2.1
Early Trabecular Ventricular Cardiomyocytes	5439	123	5316	2.3
Heart Fields	1783	5	1778	0.3
Ventricular Cardiomyocytes	7692	300	7392	3.9
Immature Ventricular Cardiomyocytes	6897	72	6825	1.0
Atrioventricular Node	486	59	427	12.1
Purkinje Fibres	1606	205	1401	12.8
Primary Heart Tube	561	5	556	0.9
Trabecular Ventricular Cardiomyocytes	4069	170	3899	4.2
Immature Atrial Cardiomyocytes	1752	17	1735	1.0
Endocardial gene-rich Cardiomyocytes	1407	2	1405	0.1
Developing Cardiomyocytes	793	5	788	0.6
Sinoatrial Node	286	24	262	8.4
Atrial Conduction System	760	29	731	3.8
Misc.	153	0	153	0.0

Supplementary Figure 11. *Dbh* expression as a percentage of each cell type expressing *Dbh.* The top panel displays the percent of each cell type expressing *Dbh* across the cardiomyocyte lineage. The bottom panel displays *Dbh* expression in terms of absolute numbers and percentages in a tabular fashion for the same data as the top panel. *Dbh* positive expression was defined as >0 counts in the raw data.

a

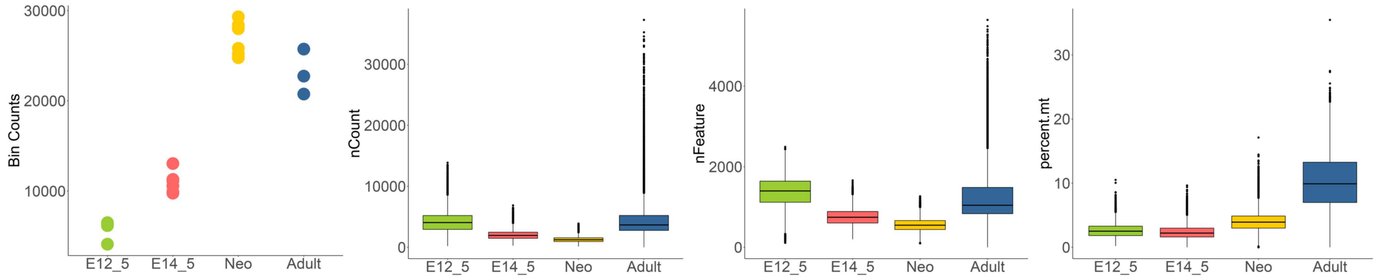

Bin Counts
30000
20000
10000
E12_5
E14_5
Neo
Adult
nCount
30000
20000
10000
0
E12_5
E14_5
Neo
Adult
nFeature
4000
2000
0
E12_5
E14_5
Neo
Adult
percent.mt
30
20
10
0
E12_5
E14_5
Neo
Adult

Supplementary Figure 12. Unsupervised clustering identified cell types across the developing murine heart using Stereo-Seq.

a) Total bin number, nCount, nFeature, and mitochondrial percentage across different stages. Neo represents P3. Adult represents P56.

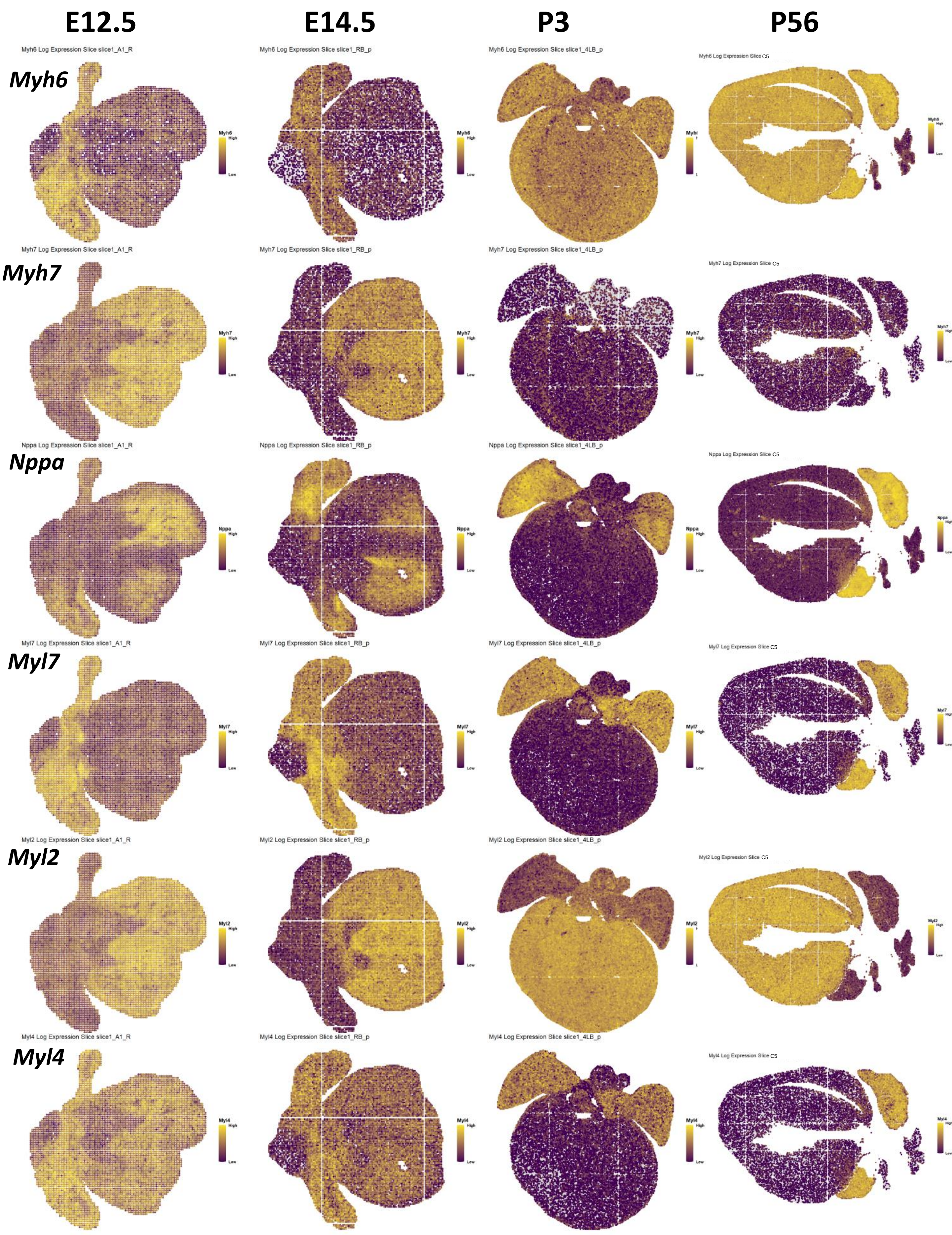
E12.5
E14.5
P3
P56
Myh6
Myh7
Nppa
Myl7
Myl2
Myl4
Myh6 Log Expression Slice slice1_A1_R
Myh6 Log Expression Slice slice1_RB_p
Myh6 Log Expression Slice slice1_4LB_p
Myh6 Log Expression Slice C5
Myh7 Log Expression Slice slice1_A1_R
Myh7 Log Expression Slice slice1_RB_p
Myh7 Log Expression Slice slice1_4LB_p
Myh7 Log Expression Slice C5
Nppa Log Expression Slice slice1_A1_R
Nppa Log Expression Slice slice1_RB_p
Nppa Log Expression Slice slice1_4LB_p
Nppa Log Expression Slice C5
Myl7 Log Expression Slice slice1_A1_R
Myl7 Log Expression Slice slice1_RB_p
Myl7 Log Expression Slice slice1_4LB_p
Myl7 Log Expression Slice C5
Myl2 Log Expression Slice slice1_A1_R
Myl2 Log Expression Slice slice1_RB_p
Myl2 Log Expression Slice slice1_4LB_p
Myl2 Log Expression Slice C5
Myl4 Log Expression Slice slice1_A1_R
Myl4 Log Expression Slice slice1_RB_p
Myl4 Log Expression Slice slice1_4LB_p
Myl4 Log Expression Slice C5
High
Low

Supplementary Figure 13a. Stereo-Seq faithfully recapitulates known gene expression changes across the developing, perinatal, and mature murine heart.
Each image displays a representative plot of the respective gene (listed at start of row), for the respective stage (listed at top of column). High gene expression is indicated by yellow and low gene expression is indicated by purple. Expression has been normalised on a per stage basis.

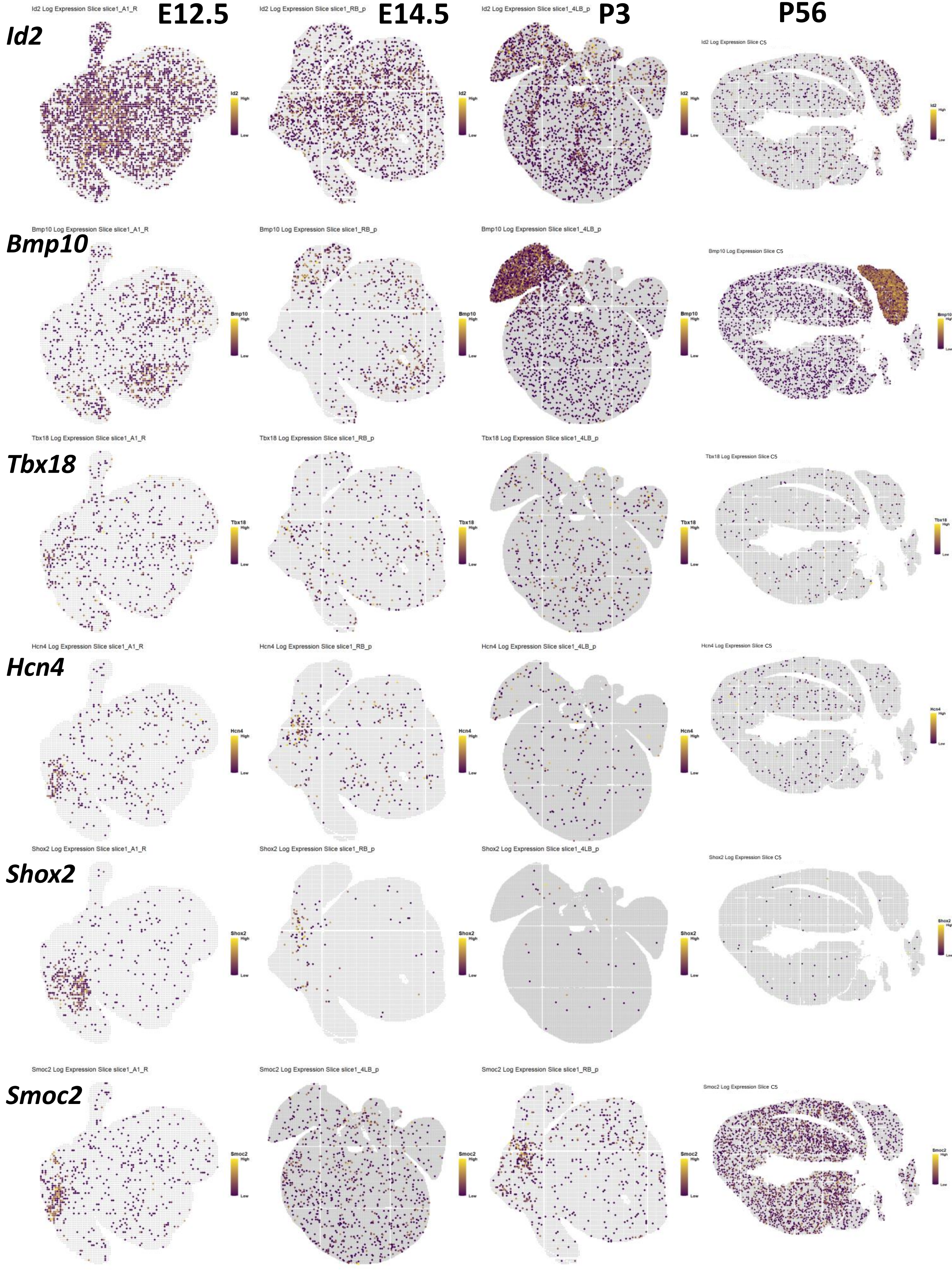
E12.5
E14.5
P3
P56
Id2
Bmp10
Tbx18
Hcn4
Shox2
Smoc2
Id2 Log Expression Slice slice1_A1_R
Id2 Log Expression Slice slice1_RB_p
Id2 Log Expression Slice slice1_4LB_p
Id2 Log Expression Slice C5
Bmp10 Log Expression Slice slice1_A1_R
Bmp10 Log Expression Slice slice1_RB_p
Bmp10 Log Expression Slice slice1_4LB_p
Bmp10 Log Expression Slice C5
Tbx18 Log Expression Slice slice1_A1_R
Tbx18 Log Expression Slice slice1_RB_p
Tbx18 Log Expression Slice slice1_4LB_p
Tbx18 Log Expression Slice C5
Hcn4 Log Expression Slice slice1_A1_R
Hcn4 Log Expression Slice slice1_RB_p
Hcn4 Log Expression Slice slice1_4LB_p
Hcn4 Log Expression Slice C5
Shox2 Log Expression Slice slice1_A1_R
Shox2 Log Expression Slice slice1_RB_p
Shox2 Log Expression Slice slice1_4LB_p
Shox2 Log Expression Slice C5
Smoc2 Log Expression Slice slice1_A1_R
Smoc2 Log Expression Slice slice1_4LB_p
Smoc2 Log Expression Slice slice1_RB_p
Smoc2 Log Expression Slice C5
High
Low

Supplementary Figure 13b. Stereo-Seq faithfully recapitulates known gene expression changes across the developing, perinatal, and mature murine heart.
Each image displays a representative plot of the respective gene (listed at start of row), for the respective stage (listed at top of column). High gene expression is indicated by yellow and low gene expression is indicated by purple. Expression has been normalised on a per stage basis.

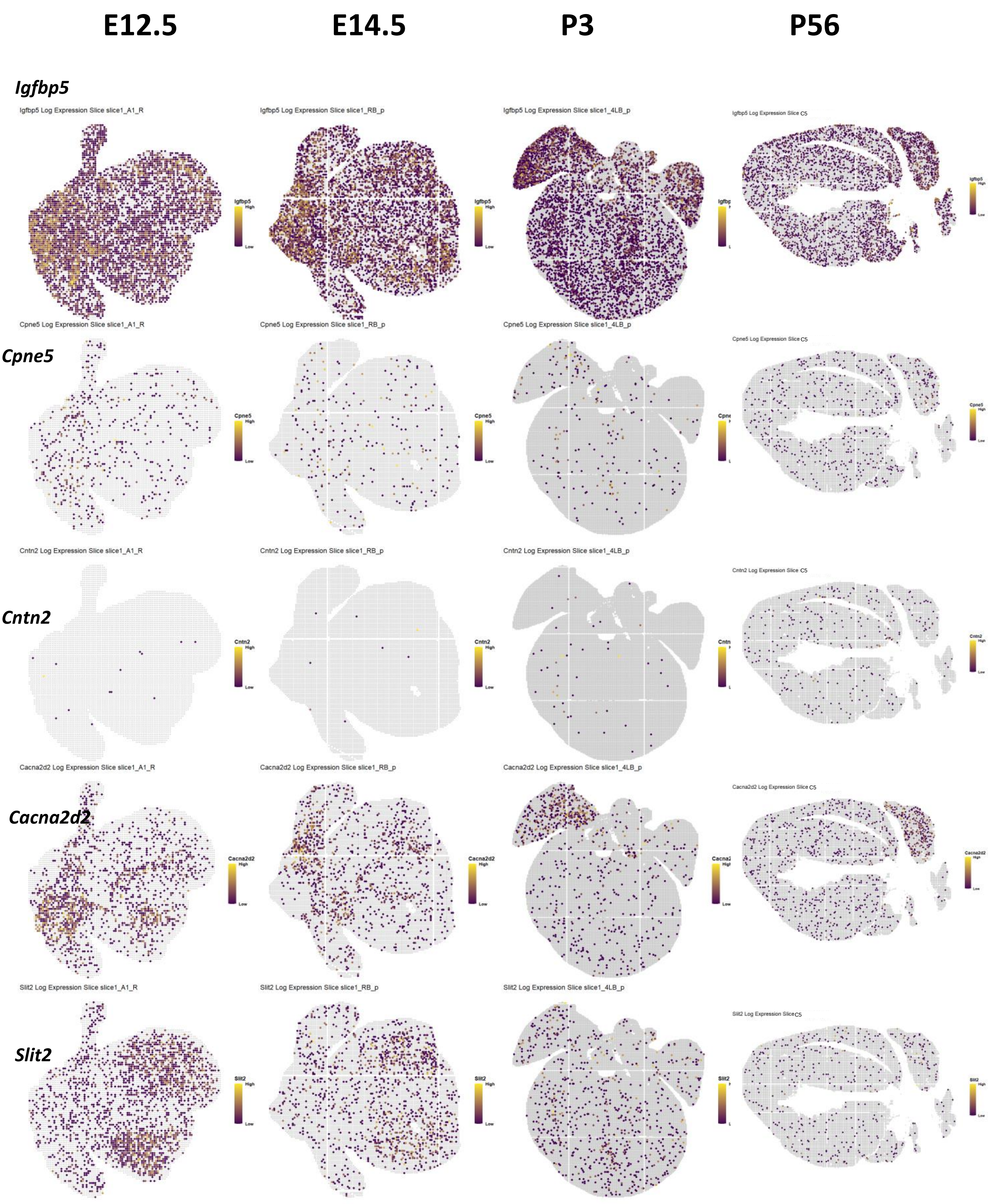
E12.5
E14.5
P3
P56
Igfbp5
Igfbp5 Log Expression Slice slice1_A1_R
Igfbp5 Log Expression Slice slice1_RB_p
Igfbp5 Log Expression Slice slice1_4LB_p
Igfbp5 Log Expression Slice C5
Cpne5
Cpne5 Log Expression Slice slice1_A1_R
Cpne5 Log Expression Slice slice1_RB_p
Cpne5 Log Expression Slice slice1_4LB_p
Cpne5 Log Expression Slice C5
Cntn2
Cntn2 Log Expression Slice slice1_A1_R
Cntn2 Log Expression Slice slice1_RB_p
Cntn2 Log Expression Slice slice1_4LB_p
Cntn2 Log Expression Slice C5
Cacna2d2
Cacna2d2 Log Expression Slice slice1_A1_R
Cacna2d2 Log Expression Slice slice1_RB_p
Cacna2d2 Log Expression Slice slice1_4LB_p
Cacna2d2 Log Expression Slice C5
Slit2
Slit2 Log Expression Slice slice1_A1_R
Slit2 Log Expression Slice slice1_RB_p
Slit2 Log Expression Slice slice1_4LB_p
Slit2 Log Expression Slice C5
High
Low

Supplementary Figure 13c. Stereo-Seq faithfully recapitulates known gene expression changes across the developing, perinatal, and mature murine heart.
Each image displays a representative plot of the respective gene (listed at start of row), for the respective stage (listed at top of column). High gene expression is indicated by yellow and low gene expression is indicated by purple. Expression has been normalised on a per stage basis.

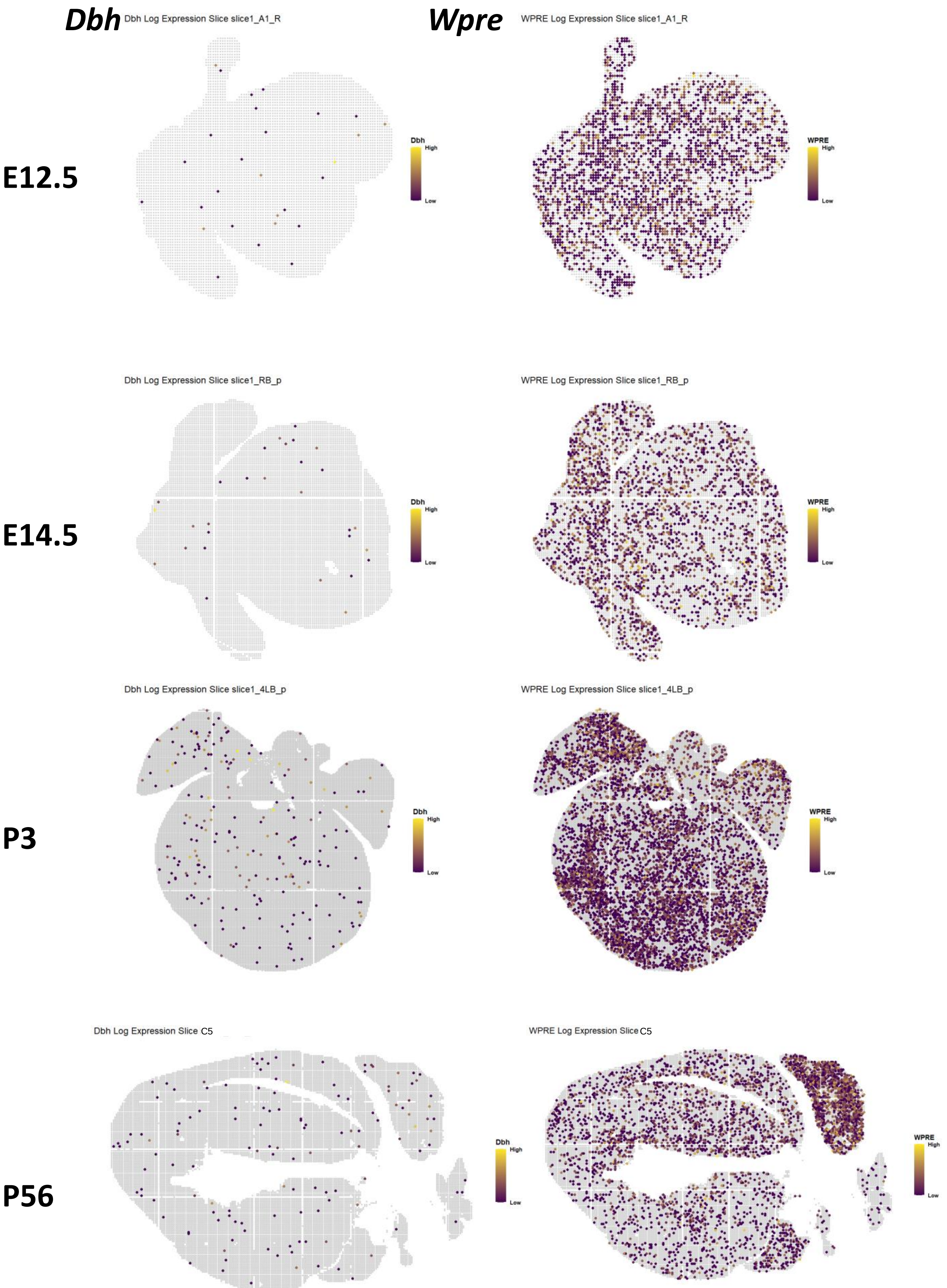
Dbh
Wpre
E12.5
E14.5
P3
P56
Dbh Log Expression Slice slice1_A1_R
WPRE Log Expression Slice slice1_A1_R
Dbh Log Expression Slice slice1_RB_p
WPRE Log Expression Slice slice1_RB_p
Dbh Log Expression Slice slice1_4LB_p
WPRE Log Expression Slice slice1_4LB_p
Dbh Log Expression Slice C5
WPRE Log Expression Slice C5
Dbh
WPRE
High
Low

Supplementary Figure 13d. Stereo-Seq faithfully recapitulates known gene expression changes across the developing, perinatal, and mature murine heart.
Each image displays a representative plot of the respective gene (listed at start of row), for the respective stage (listed at top of column). High gene expression is indicated by yellow and low gene expression is indicated by purple. Expression has been normalised on a per stage basis. N.B. *Dbh* and *Wpre vs* developmental stages have been transposed with respect to rows vs columns for genes and stages as in other parts of this figure.

a

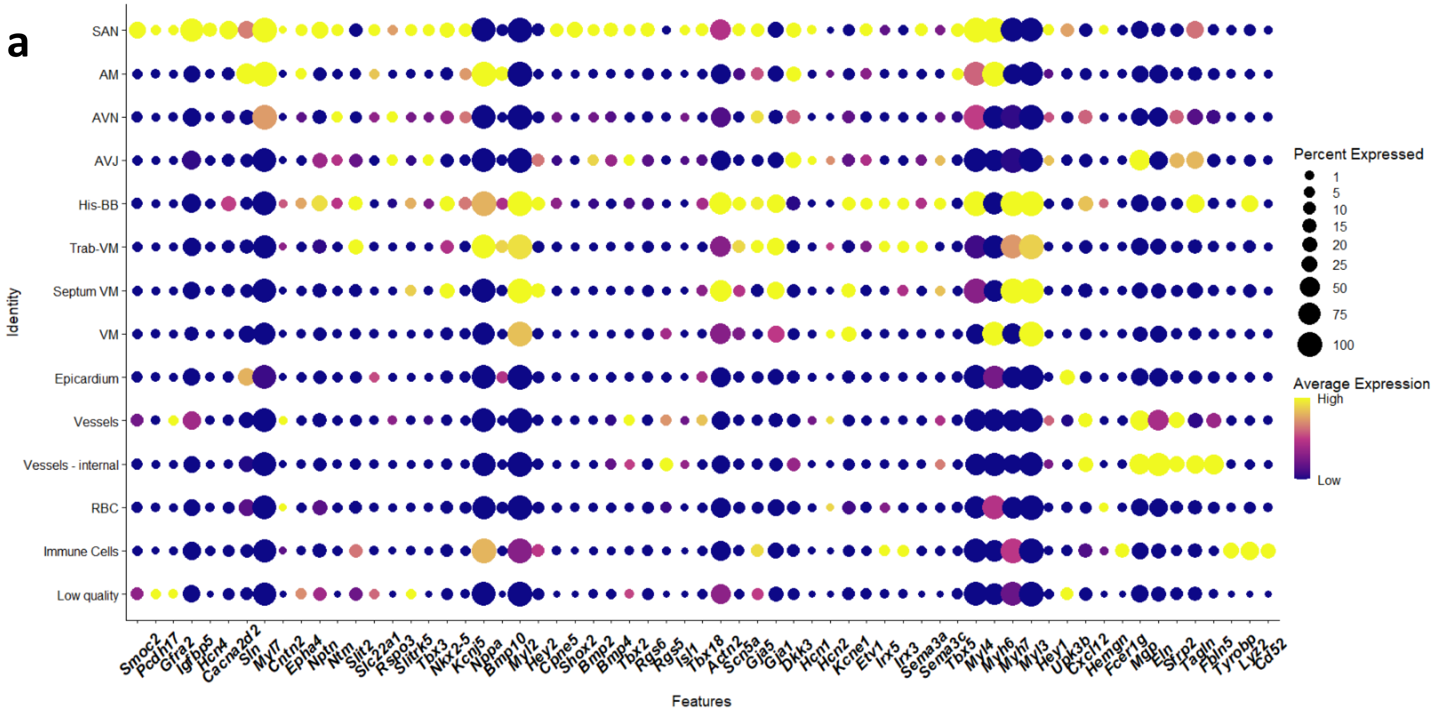

Identity
SAN
AM
AVN
AVJ
His-BB
Trab-VM
Septum VM
VM
Epicardium
Vessels
Vessels - internal
RBC
Immune Cells
Low quality
Smoc2
Pcdh17
Gfra2
Igfbp5
Hcn4
Cacna2d2
Sln
Myl7
Cntn2
Epha4
Nptn
Ntm
Slit2
Slc22a1
Rspo3
Slitrk5
Tbx3
Nkx2-5
Kcnj5
Nppa
Bmp10
Myl2
Hey2
Cpne5
Shox2
Bmp2
Bmp4
Tbx2
Rgs6
Rgs5
Isl1
Tbx18
Actn2
Scn5a
Gja5
Gja1
Dkk3
Hcn1
Hcn2
Kcne1
Etv1
Irx5
Irx3
Sema3a
Sema3c
Tbx5
Myl4
Myh6
Myh7
Myl3
Hey1
Upk3b
Cxcl12
Hemgn
Fcer1g
Mgp
Eln
Sfrp2
Tagln
Fbln5
Tyrobp
Lyz2
Cd52
Features
Percent Expressed
1
5
10
15
20
25
50
75
100
Average Expression
High
Low

Supplementary Figure 14. Unsupervised clustering identified cell types across the developing murine heart.
a) A dot plot demonstrating scaled expression of selected genes across cell types identified through unsupervised clustering of E12.5, E14.5, and P3 samples. The expression has been scaled for visualization purposes.

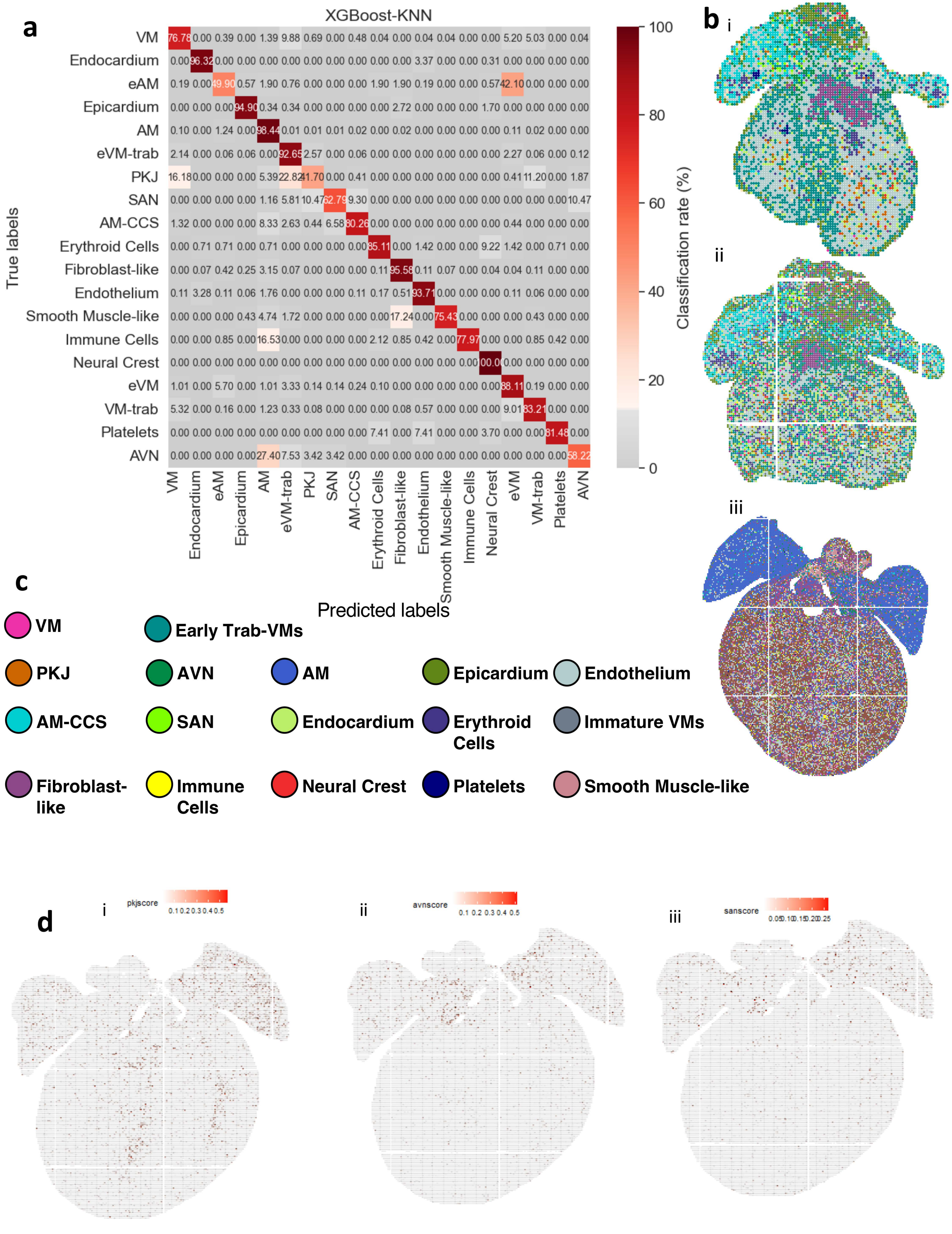

a
XGBoost-KNN
True labels
Predicted labels
Classification rate (%)
0
20
40
60
80
100
VM 76.78 0.00 0.39 0.00 1.39 9.88 0.69 0.00 0.48 0.04 0.00 0.04 0.04 0.00 0.00 5.20 5.03 0.00 0.04
Endocardium 0.00 96.32 0.00 0.00 0.00 0.00 0.00 0.00 0.00 0.00 0.00 3.37 0.00 0.00 0.31 0.00 0.00 0.00 0.00
eAM 0.19 0.00 49.90 0.57 1.90 0.76 0.00 0.00 0.00 1.90 1.90 0.19 0.00 0.00 0.57 42.10 0.00 0.00 0.00
Epicardium 0.00 0.00 0.00 94.90 0.34 0.34 0.00 0.00 0.00 0.00 2.72 0.00 0.00 0.00 1.70 0.00 0.00 0.00 0.00
AM 0.10 0.00 1.24 0.00 98.44 0.01 0.01 0.01 0.02 0.00 0.02 0.00 0.00 0.00 0.00 0.11 0.02 0.00 0.00
eVM-trab 2.14 0.00 0.06 0.06 0.00 92.65 2.57 0.00 0.06 0.00 0.00 0.00 0.00 0.00 0.00 2.27 0.06 0.00 0.12
PKJ 16.18 0.00 0.00 0.00 5.39 22.82 41.70 0.00 0.41 0.00 0.00 0.00 0.00 0.00 0.00 0.41 11.20 0.00 1.87
SAN 0.00 0.00 0.00 0.00 1.16 5.81 10.47 62.79 9.30 0.00 0.00 0.00 0.00 0.00 0.00 0.00 0.00 0.00 10.47
AM-CCS 1.32 0.00 0.00 0.00 8.33 2.63 0.44 6.58 80.26 0.00 0.00 0.00 0.00 0.00 0.00 0.44 0.00 0.00 0.00
Erythroid Cells 0.00 0.71 0.71 0.00 0.71 0.00 0.00 0.00 0.00 85.11 0.00 1.42 0.00 0.00 9.22 1.42 0.00 0.71 0.00
Fibroblast-like 0.00 0.07 0.42 0.25 3.15 0.07 0.00 0.00 0.00 0.11 95.58 0.11 0.07 0.00 0.04 0.04 0.11 0.00 0.00
Endothelium 0.11 3.28 0.11 0.06 1.76 0.00 0.00 0.00 0.11 0.17 0.51 93.71 0.00 0.00 0.00 0.11 0.06 0.00 0.00
Smooth Muscle-like 0.00 0.00 0.00 0.43 4.74 1.72 0.00 0.00 0.00 0.00 17.24 0.00 75.43 0.00 0.00 0.00 0.43 0.00 0.00
Immune Cells 0.00 0.00 0.85 0.00 16.53 0.00 0.00 0.00 0.00 2.12 0.85 0.42 0.00 77.97 0.00 0.00 0.85 0.42 0.00
Neural Crest 0.00 0.00 0.00 0.00 0.00 0.00 0.00 0.00 0.00 0.00 0.00 0.00 0.00 0.00 00.0 0.00 0.00 0.00 0.00
eVM 1.01 0.00 5.70 0.00 1.01 3.33 0.14 0.14 0.24 0.10 0.00 0.00 0.00 0.00 0.00 88.11 0.19 0.00 0.00
VM-trab 5.32 0.00 0.16 0.00 1.23 0.33 0.08 0.00 0.00 0.00 0.08 0.57 0.00 0.00 0.00 9.01 83.21 0.00 0.00
Platelets 0.00 0.00 0.00 0.00 0.00 0.00 0.00 0.00 0.00 7.41 0.00 7.41 0.00 0.00 3.70 0.00 0.00 81.48 0.00
AVN 0.00 0.00 0.00 0.00 27.40 7.53 3.42 3.42 0.00 0.00 0.00 0.00 0.00 0.00 0.00 0.00 0.00 0.00 58.22
VM Endocardium eAM Epicardium AM eVM-trab PKJ SAN AM-CCS Erythroid Cells Fibroblast-like Endothelium Smooth Muscle-like Immune Cells Neural Crest eVM VM-trab Platelets AVN
b
i
ii
iii
c
VM
Early Trab-VMs
PKJ
AVN
AM
Epicardium
Endothelium
AM-CCS
SAN
Endocardium
Erythroid Cells
Immature VMs
Fibroblast-like
Immune Cells
Neural Crest
Platelets
Smooth Muscle-like
d
i
pkjscore
0.1 0.2 0.3 0.4 0.5
ii
avnscore
0.1 0.2 0.3 0.4 0.5
iii
sanscore
0.05 0.10 0.15 0.20 0.25

Supplementary Figure 15. Integrated single-cell and spatial transcriptomics resolves even rare cell types across the heart, but retains mismatch between transcriptomic and biological feature importance.

a) Supervised machine learning techniques can produce accurate classifiers for most cell types based on gene expression and marker gene selection. We used SMASH to identify biologically relevant marker genes with robust computational signatures sufficient to facilitate cross-dataset and cross-platform supervised learning based classification of pixels within our spatial transcriptomics data with maximally informative, but interpretable, unique gene sets per cell type as classification label. This matrix demonstrates the highly accurate classification of cell types by a trained classifier, on our separate test data, for subsequent identification of cell type marker genes in single-cell RNA sequencing data by SMASH. Y-axis true labels refers to the set of cell-wise labels provided for our dataset, based on our initial analysis. The x-axis predicted labels are the same labels, with a high number (dark red) at the intersect of the two in the main body of the matrix indicating that the trained classifier was able to correctly identify the cell label. The scale shows the percentage of classification, with dark red indicating a higher percentage of the true label on y, was predicted to be the cell type on the x axis. Each row sums to 100.00, representing all the cells within the given cell type label on the y-axis, have been classified with predicted label(s).

b) Representatives slices from E12.5 (i), E14.5 (ii), and P3 (iii), coloured by cell type identified through RCTD predicted cell type and corresponding to cell labels in panel c.

c) Labels for cell types of respective colour in panel b.

d) i) Slices from P3 hearts, coloured by the RCTD predicted score for Purkinje fibres, with red indicating higher likelihood. ii) Slices from P3 hearts, coloured by the RCTD predicted score for the atrioventricular node, with red indicating higher likelihood. iii) Slices from P3 hearts, coloured by the RCTD predicted score for the sinoatrial node, with red indicating higher likelihood. All slices are presented in the same anatomical orientation with respect to left to right.

Abbreviations: VM - ventricular cardiomyocytes; eAM –immature atrial cardiomyocytes; AM – atrial cardiomyocytes; eVM-trab – early trabecular ventricular cardiomyocytes; PKJ – Purkinje fibres; SAN – sinoatrial node; AM-CCS – atrial cardiac conduction system; eVM – immature ventricular cardiomyocytes; VM-trab – trabecular ventricular cardiomyocytes; AVN – atrioventricular node.

RNAscope

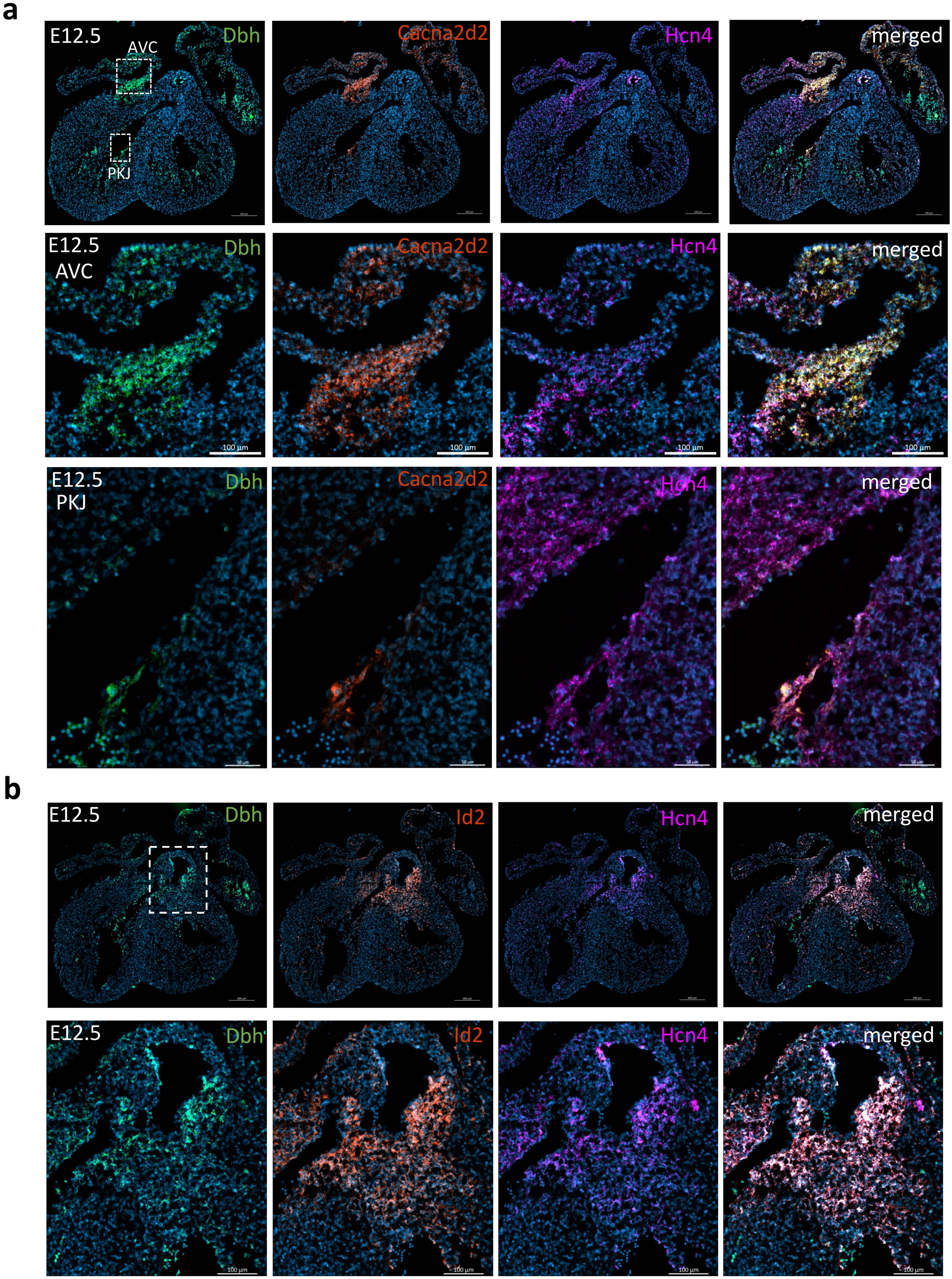
a
E12.5
AVC
PKJ
Dbh
Cacna2d2
Hcn4
merged
E12.5
AVC
Dbh
Cacna2d2
Hcn4
merged
100 µm
E12.5
PKJ
Dbh
Cacna2d2
Hcn4
merged
b
E12.5
Dbh
Id2
Hcn4
merged
E12.5
Dbh
Id2
Hcn4
merged
100 µm

Supplementary Figure 16. The use of RNAscope to confirm the expression pattern of Dbh and CCS markers in E12.5 hearts.

a) Representative RNAscope images of the distribution of Dbh (green), CCS markers Cacna2d2 (red) and Hcn4 (magenta) in a whole field of E12.5 heart. The second row shows SV and the third row show PKJ.

b) Representative RNAscope images of the distribution of Dbh (green), CCS markers Id2 (red) and Hcn4 (magenta) in a whole field of E12.5 heart.

SV: Sinus Venosus

AVC: Atrioventricular canal

PKJ: Purkinje fiber network

Scale Bar:

a) top panel: 200μm; middle panel: 100μm; bottom panel: 50μm;

b) top panel: 200μm; bottom panel: 100μm

a

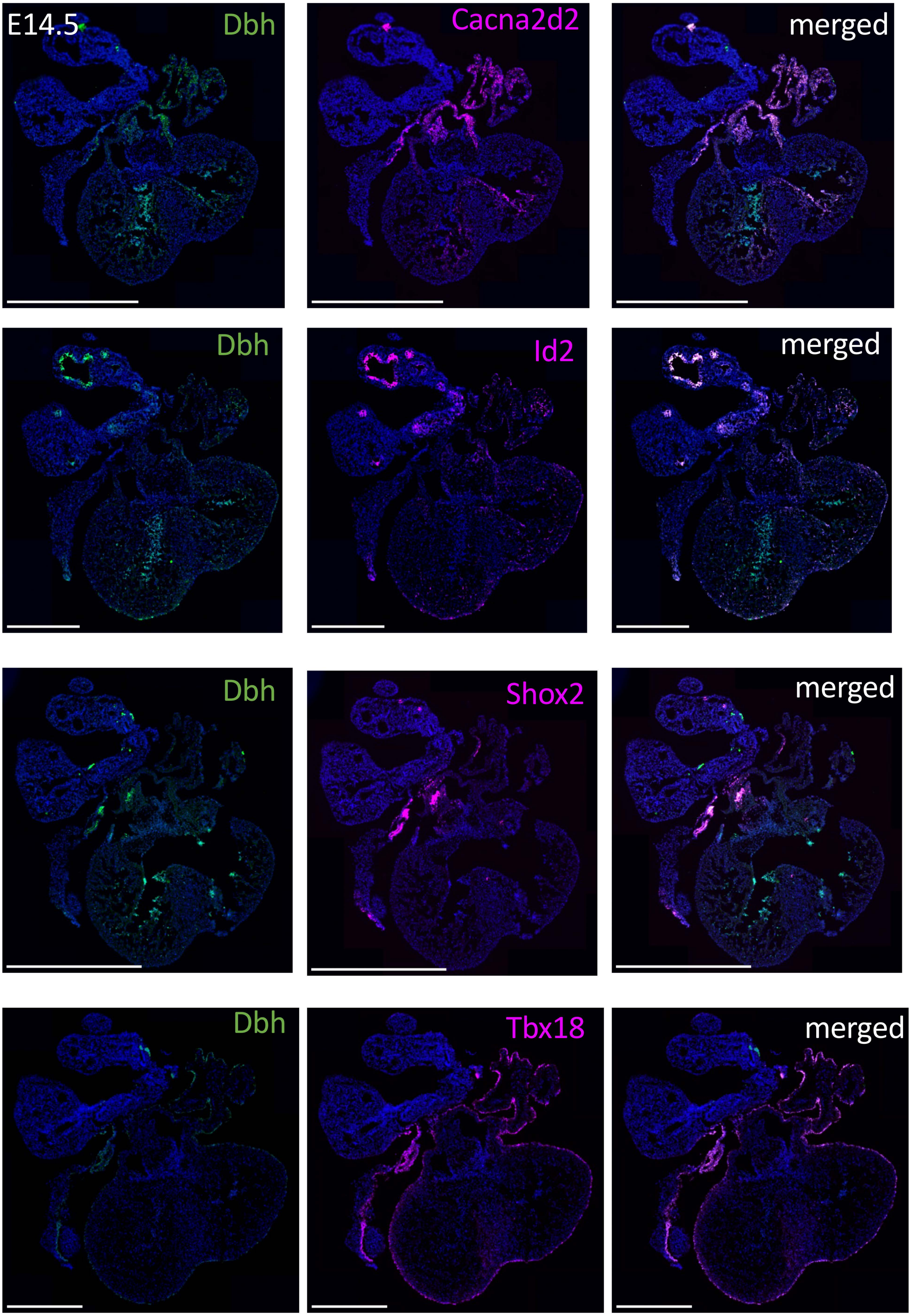
E14.5
Dbh
Cacna2d2
merged
Dbh
Id2
merged
Dbh
Shox2
merged
Dbh
Tbx18
merged

Supplementary Figure 17. Spatio-temporal lineage tracing identified the association between *Dbh*+ cells and cardiac conduction system (CCS) at E14.5 and P3

a) RNAscope showing the distribution of Dbh probe (green) with CCS markers including Cacna2d2 (magenta), Id2(magenta), Shox2(magenta), Tbx18(magenta) in the whole field images of E14.5. Scale Bar: 500μm

b

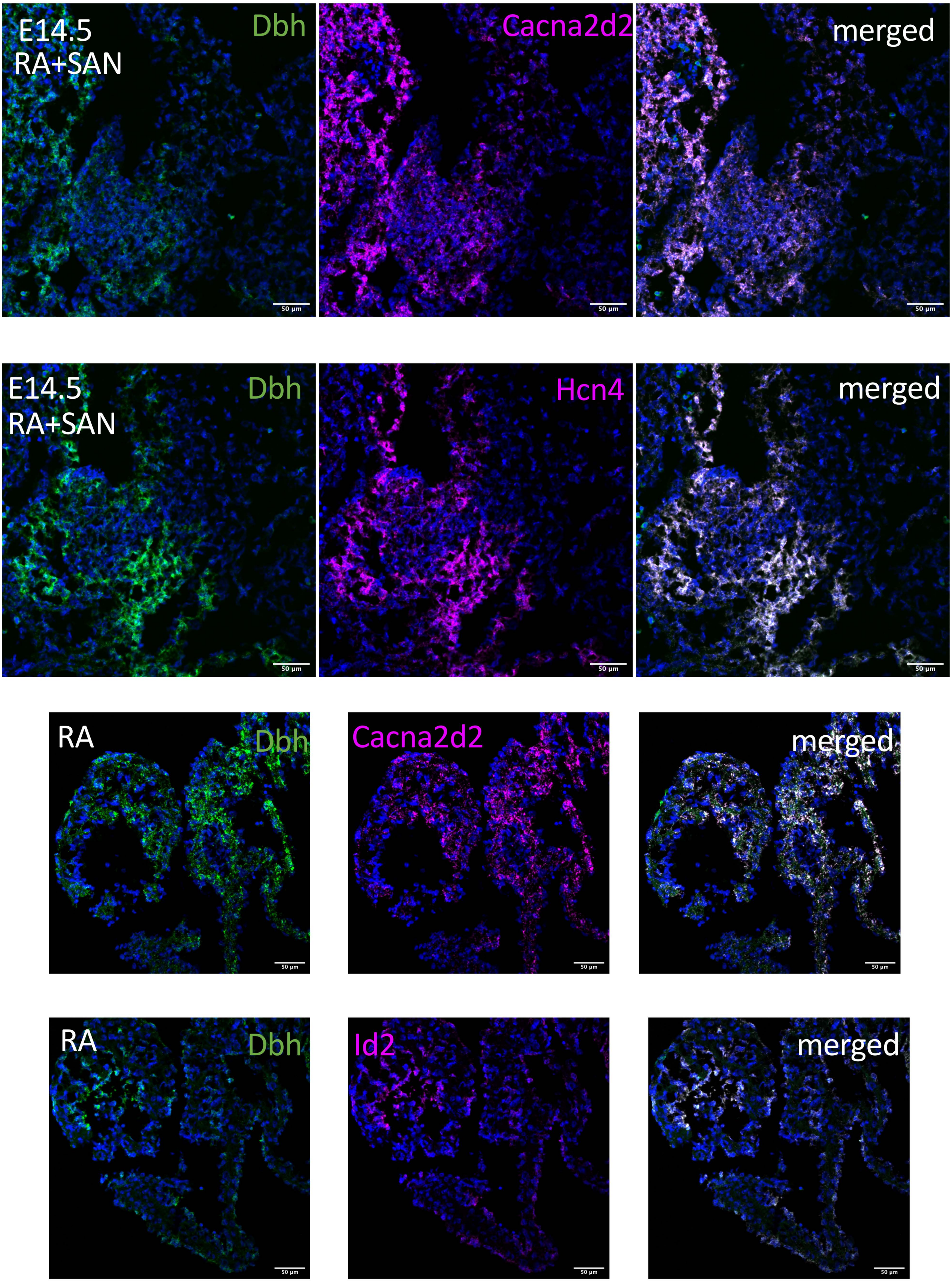
E14.5
RA+SAN
Dbh
Cacna2d2
merged
50 µm
E14.5
RA+SAN
Dbh
Hcn4
merged
50 µm
RA
Dbh
Cacna2d2
merged
50 µm
RA
Dbh
Id2
merged
50 µm

Supplementary Figure 17. Spatio-temporal lineage tracing identified the association between *Dbh*+ cells and cardiac conduction system (CCS) at E14.5 and P3
b) RNAscope showing the coexpression of Dbh probe (green) with CCS markers including Cacna2d2 (magenta), Id2(magenta), Hcn4(magenta) at RA and SAN regions at E14.5. Scale Bar: 50μm

c

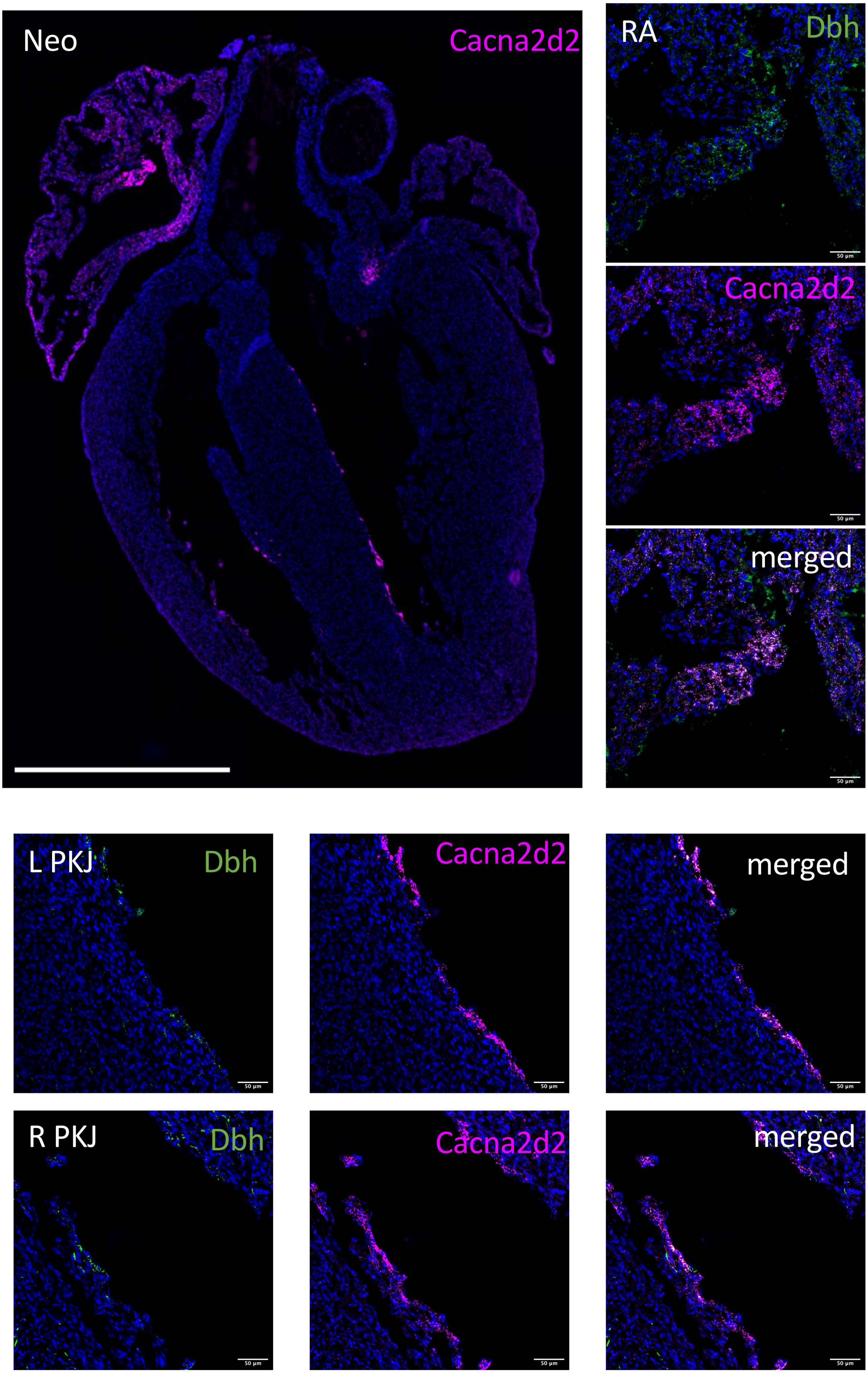

Neo
Cacna2d2
RA
Dbh
Cacna2d2
merged
L PKJ
Dbh
Cacna2d2
merged
R PKJ
Dbh
Cacna2d2
merged
50 μm

Supplementary Figure 17. Spatio-temporal lineage tracing identified the association between *Dbh*+ cells and cardiac conduction system (CCS) at E14.5 and P3
c) RNAscope showing the distribution of Dbh (green) with CCS marker Cacna2d2 (magenta) at RA and left and right PKJ regions at P3. Scale Bar: 500μm (whole field); 50μm

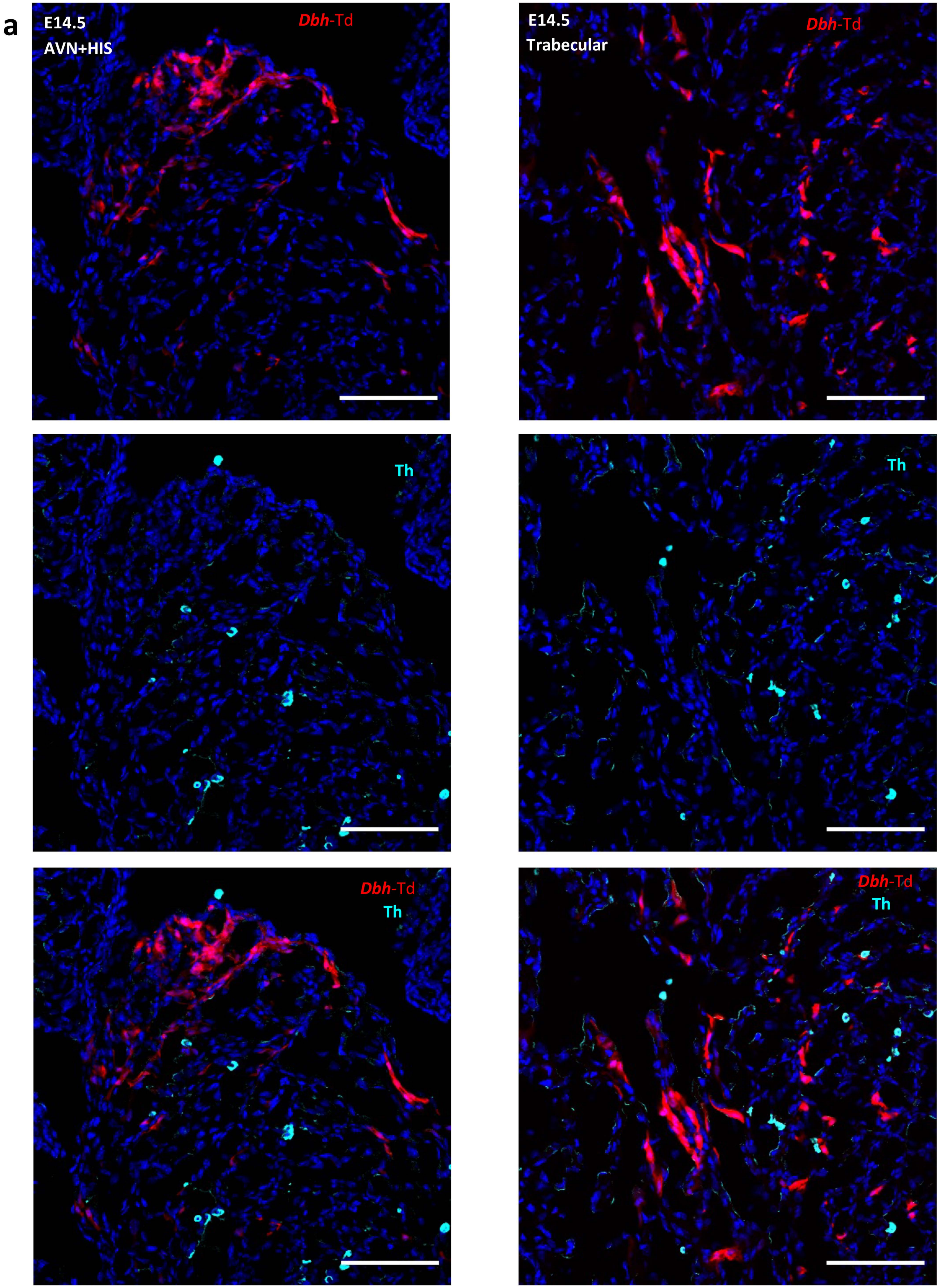
a
E14.5
AVN+HIS
Dbh-Td
E14.5
Trabecular
Dbh-Td
Th
Th
Dbh-Td
Th
Dbh-Td
Th

Supplementary Figure 18a.Immunofluorescence of Dbh+ CMs in AVN+HIS bundle and Trabecular regions stained with Th (cyan) revealing the association between *Dbh*+ derived CMs with sympathetic innervation at E14.5.
scale bar: 100um
LA: Left Atrium
RA: Right Atrium
LV: Left Ventricle
RV: Right Ventricle
AVN: Atrioventricular node,
HIS: Bundle of His

b

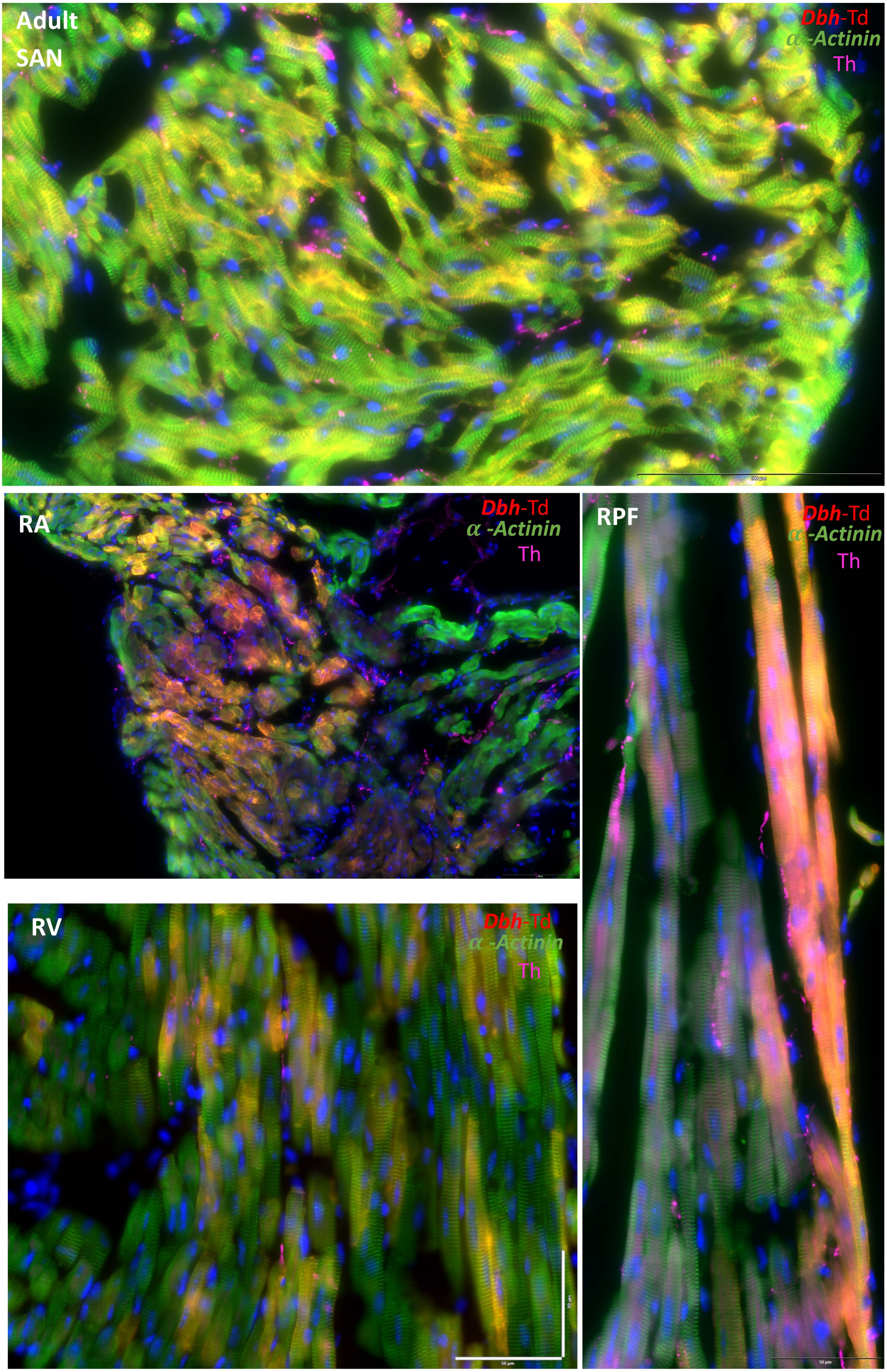

Adult
SAN
Dbh-Td
α-Actinin
Th
RA
Dbh-Td
α-Actinin
Th
RPF
Dbh-Td
α-Actinin
Th
RV
Dbh-Td
α-Actinin
Th

Supplementary Figure 18b. Immunofluorescence of Dbh+ derived CMs in SAN, RA,RV and RPF regions stained α-actinin (green) and Th (magenta) revealing the Dbh+ CMs and the association with sympathetic innervation at the adult stage.
Scale bar: 100um
LA: Left Atrium
RA: Right Atrium
LV: Left Ventricle
RV: Right Ventricle
AVN: Atrioventricular node,
HIS: Bundle of His

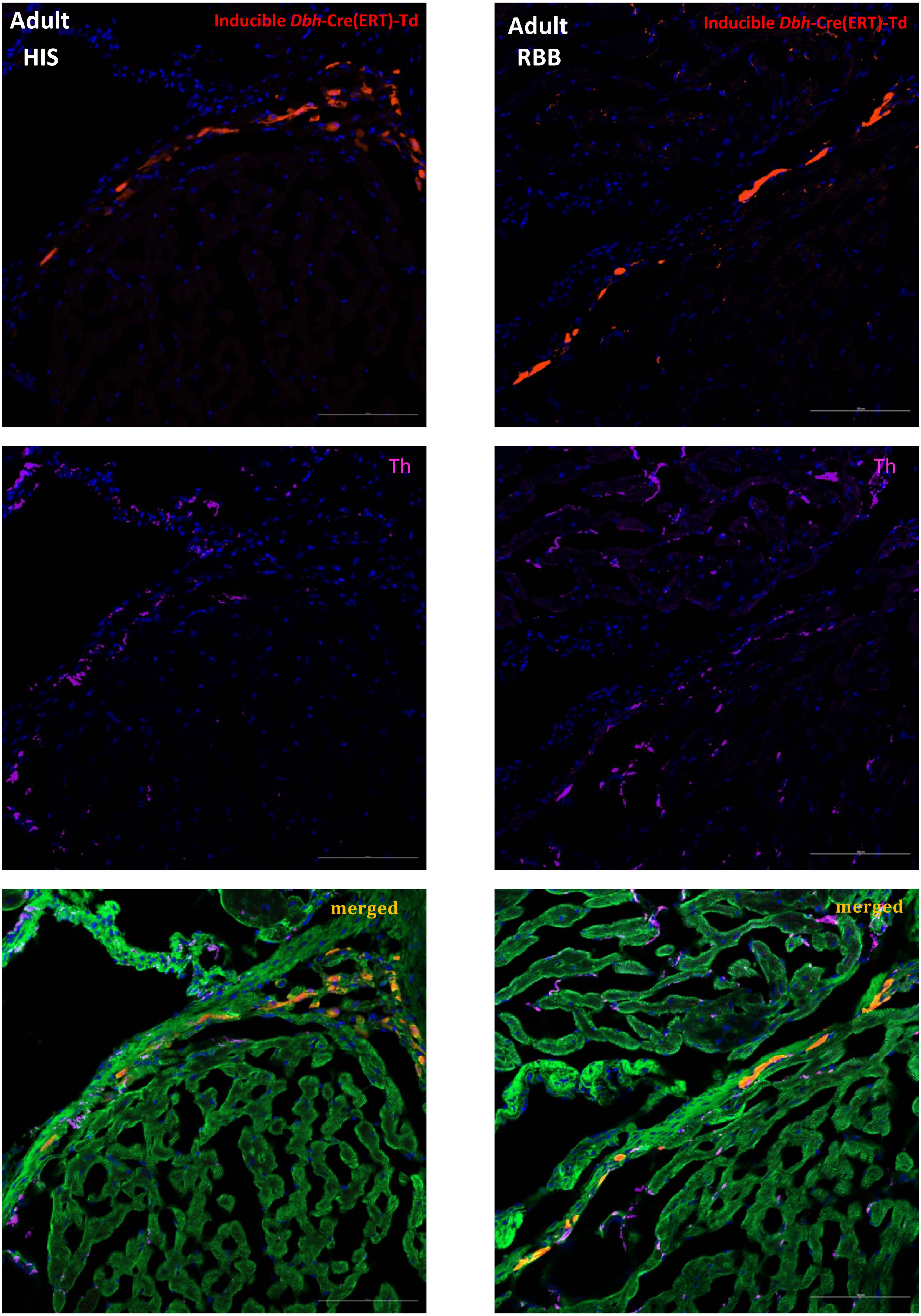
Adult
HIS
Inducible Dbh-Cre(ERT)-Td
Adult
RBB
Inducible Dbh-Cre(ERT)-Td
Th
Th
merged
merged

Supplementary Figure 19. Immunofluorescence of Dbh+ CMs in HIS and RBB regions stained α-actinin (green) and Th (magenta) revealing the Dbh+ CMs and the association with sympathetic innervation at the adult stage by using *Dbh*$_{CreERT}$/R26-tdTomato inducible reporter mouse line Scale Bar:100 μm,

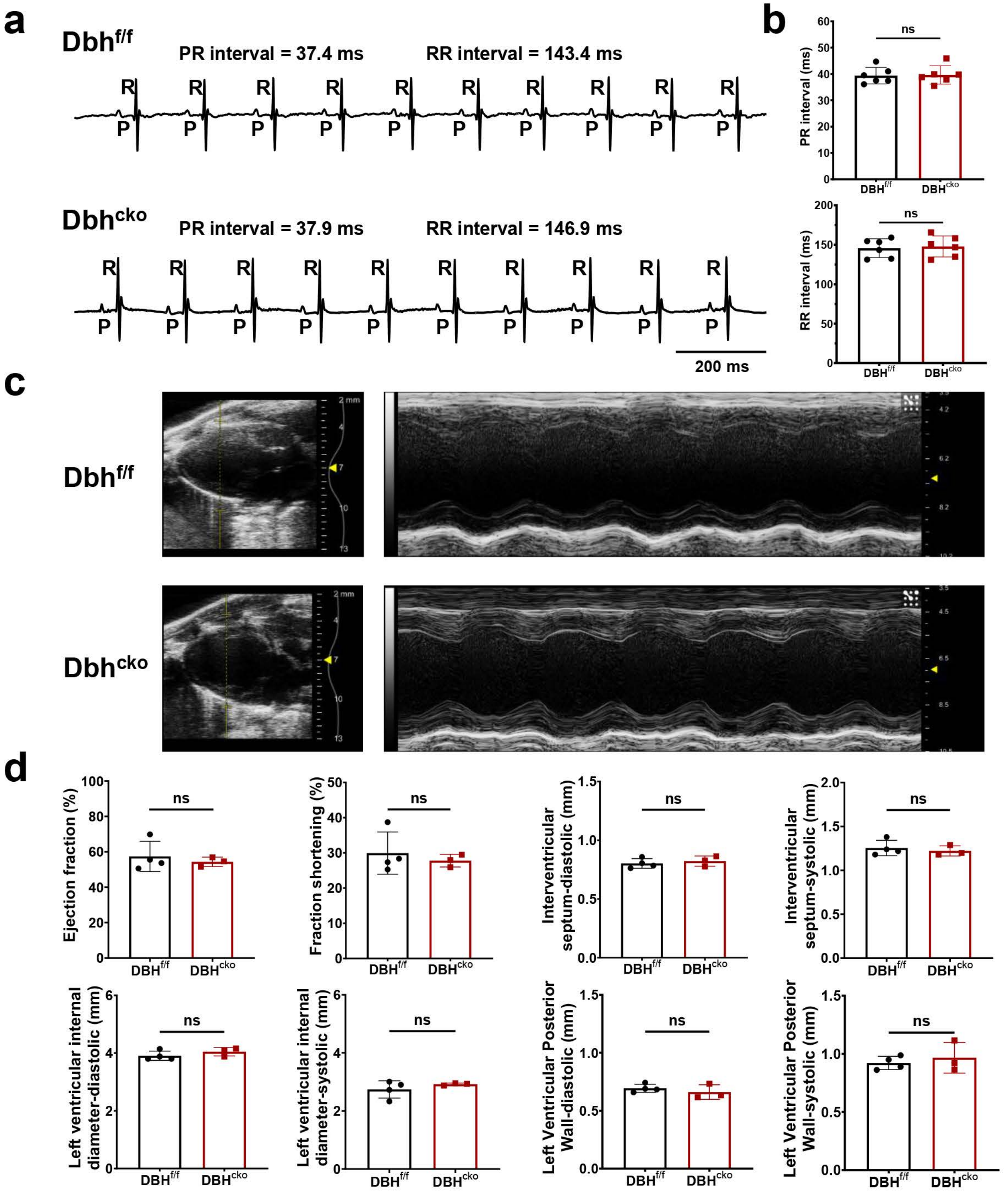
a
Dbh^f/f
PR interval = 37.4 ms
RR interval = 143.4 ms
Dbh^cko
PR interval = 37.9 ms
RR interval = 146.9 ms
200 ms
b
PR interval (ms)
RR interval (ms)
ns
DBH^f/f
DBH^cko
c
Dbh^f/f
Dbh^cko
d
Ejection fraction (%)
Fraction shortening (%)
Interventricular septum-diastolic (mm)
Interventricular septum-systolic (mm)
Left ventricular internal diameter-diastolic (mm)
Left ventricular internal diameter-systolic (mm)
Left Ventricular Posterior Wall-diastolic (mm)
Left Ventricular Posterior Wall-systolic (mm)

Supplementary Figure 20. Characterization of Dbh$_{cko}$ and Dbh$_{f/f}$ mice by electrography and echocardiography Typical ECG traces of isolated hearts at baseline from Dbh$_{cko}$ and Dbh$_{f/f}$ mice.

a) Representative ECG traces for Dbh$_{cko}$ and Dbh$_{f/f}$ mice under anaesthesia condition

b) Echocardiography analysis and comparison of P-R, RR intervals

c) Representative echocardiographic images obtained by short (left) and long (right) axis scanning from Dbhf/f and Dbhcko mice

d) Analysis of echocardiographic parameters including ejection fraction (EF), fractional shortening (FS), interventricular septum-diastolic, interventricular septum-systolic, left ventricular internal diameter diastolic, left ventricular internal diameter-systolic, left ventricular posterior wall-diastolic, left ventricular posterior wall-systolic. n=5-6 per group, * p<0.05, ** p< 0.01, + p>0.05.

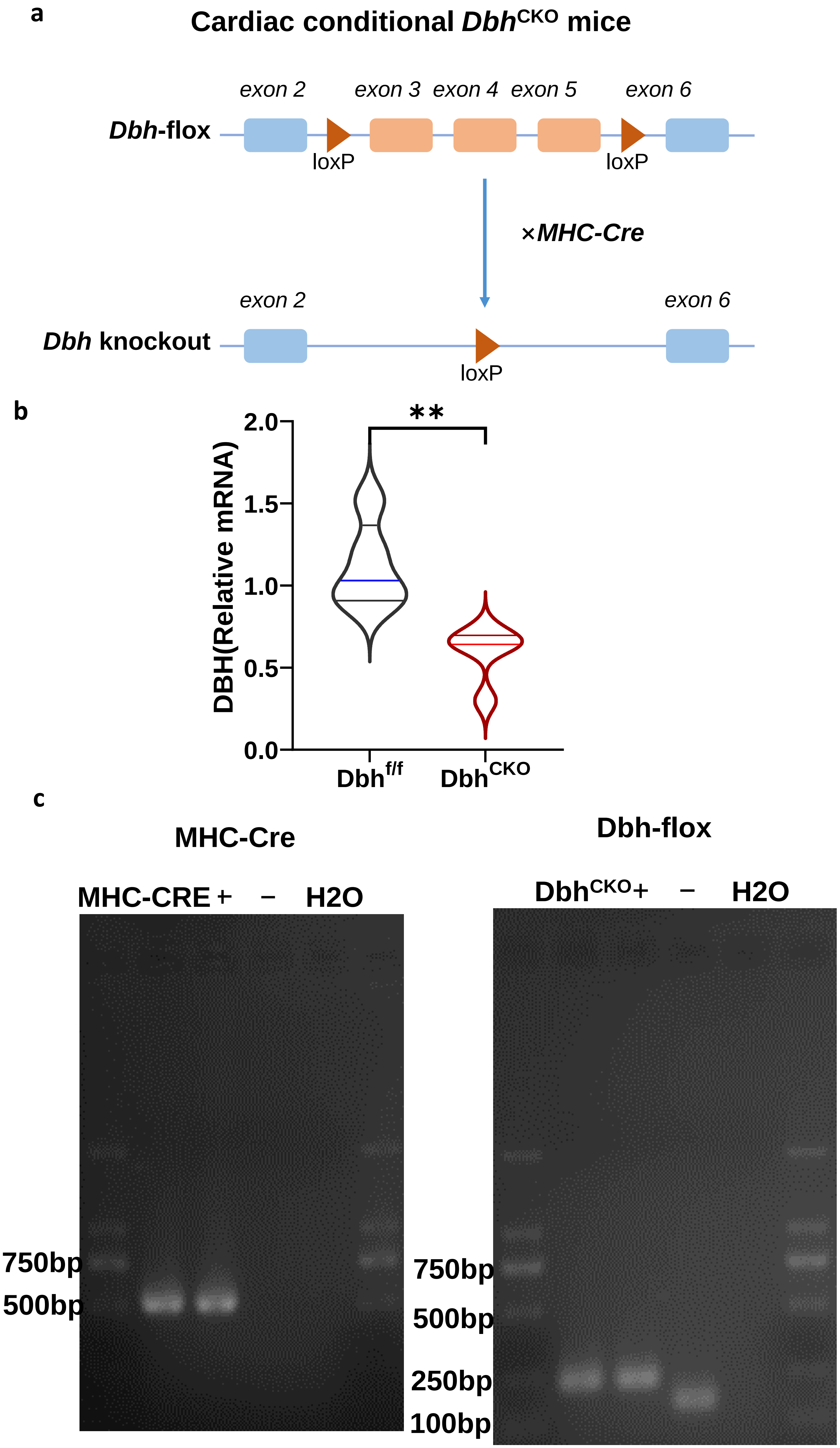
a
Cardiac conditional Dbh^CKO mice
exon 2
exon 3
exon 4
exon 5
exon 6
Dbh-flox
loxP
loxP
×MHC-Cre
exon 2
exon 6
Dbh knockout
loxP
b
DBH(Relative mRNA)
2.0
1.5
1.0
0.5
0.0
**
Dbh^f/f
Dbh^CKO
c
MHC-Cre
MHC-CRE + − H2O
750bp
500bp
Dbh-flox
Dbh^CKO + − H2O
750bp
500bp
250bp
100bp

Supplementary Figure 21. Construction and validation of cardiac conditional Dbh_{CKO} mouse model.

a) A Schematic graph showing the construction of cardiac conditional Dbh_{CKO} mouse model by crossing the Dbh-flox mouse with MHC-Cre mouse line.

b) Violin plot of evaluation on Cre mRNA expression between $Dbh_{f/f}$ and Dbh_{CKO} showing the knockout efficiency.

c) Western blot results showing the genotype of MHC-Cre and Dbh-flox mouse lines

	p_val	avg_log2FC	pct.1	pct.2	p_val_adj
Myl1	0	-0.82132	0.029	0.501	0
Rpl31	0	0.664781	0.815	0.292	0
Tnni1	0	0.55242	0.97	0.828	0
Mif	0	0.549106	0.801	0.403	0
Uqcr11	0	0.657822	0.93	0.685	0
Rpl41	0	0.536845	0.985	0.899	0
Pln	0	0.754825	0.917	0.55	0
Atp5b	0	-0.54387	0.753	0.832	0
Myl7	0	-2.86116	0.595	0.918	0
Grb10	0	0.602064	0.587	0.102	0
Sparc	0	-0.72186	0.1	0.52	0
Gpx3	0	-0.88944	0.063	0.563	0
Ubb	0	-0.9398	0.217	0.698	0
Myl4	0	-1.77982	0.595	0.916	0
Rpl38	0	0.700118	0.837	0.35	0
Rps29	0	0.810815	0.958	0.733	0
2010107E0	0	0.532544	0.933	0.669	0
Cox7c	0	0.842176	0.954	0.669	0
Myh6	0	-0.70168	0.239	0.589	0
Myh7	0	2.268165	0.919	0.153	0
Tpt1	0	0.659201	0.966	0.673	0
Cox6c	0	0.666663	0.979	0.839	0
Rpl3	0	-0.63665	0.34	0.682	0
Lgals1	0	-0.7515	0.149	0.543	0
Fam162a	0	0.689374	0.724	0.236	0
Cox17	0	0.570492	0.666	0.214	0
Rps28	0	0.59056	0.833	0.366	0
Rpl36	0	0.551748	0.93	0.651	0
Lbh	0	0.586679	0.597	0.128	0
Malat1	0	0.812705	0.946	0.877	0
Cox8a	0	0.766928	0.964	0.739	0
Acta2	0	0.697328	0.593	0.302	0
Usmg5	0	0.834194	0.919	0.549	0
Atp5g3	0	0.60912	0.919	0.535	0
Ttn	0	1.532177	0.924	0.277	0
Actc1	0	0.916254	0.997	0.924	0
Cst3	0	-0.64277	0.146	0.526	0
Atp5e	0	0.764136	0.916	0.625	0
Myl9	0	-0.73747	0.248	0.652	0
Rps21	0	0.716165	0.865	0.356	0
Ndufc1	0	0.535632	0.855	0.478	0
Nexn	0	0.886403	0.83	0.298	0
Gng5	0	0.502276	0.798	0.33	0
Nppa	0	-3.10881	0.16	0.804	0
Tomm7	0	0.506746	0.685	0.244	0
Atp5k	0	0.866033	0.904	0.53	0

Cox6a1	0	0.547064	0.846	0.507	0
Ubc	0	-0.50716	0.088	0.406	0
Actb	0	-0.57212	0.052	0.331	0
Myl2	0	1.419547	0.966	0.483	0
Cycs	0	0.514156	0.809	0.421	0
Gapdh	0	0.872422	0.936	0.651	0
Tnni3	0	-0.61438	0.932	0.905	0
Hbb-bs	0	0.569781	0.783	0.22	0
Cox8b	0	0.641144	0.7	0.355	0
H19	0	0.680188	0.614	0.253	0
Ndufa3	0	0.727311	0.81	0.313	0
Cox7a1	0	0.863614	0.746	0.266	0
Mt1	0	0.89644	0.724	0.274	0
Sln	0	-2.22211	0.021	0.747	0
Myl3	0	1.278612	0.984	0.763	0
mt-Nd1	0	1.169594	0.992	0.899	0
mt-Nd2	0	1.470762	0.969	0.504	0
mt-Co1	0	0.585344	0.713	0.24	0
mt-Nd4	0	0.625096	0.971	0.767	0
mt-Nd5	0	0.651863	0.777	0.245	0
Rpl39	0	0.825846	0.942	0.536	0
Tmsb4x	0	-0.64044	0.576	0.809	0

Table S1 (Related to Figure 1)**:** Differential expression of genes between cell types within our cardiomyocyte lineage. Each sheet corresponds to its respective cell type. p_val is the pvalue for a significantly differentially expressed gene. Avg_log2FC is the average log2 fold change for this gene compared from this cell type to all other cell types. Pct.1 is the percentage of cells expressing this gene in this given cell type, and Pct.2 is for the percentage of cells expressing this gene in all other cell types. P_val_adj is the p-value adjusted for multiple hypothesis testing by Bonferroni’s correction.

	AM	AM-CCS	AVN	Endocardiu	Endotheliu	Epicardium	Erythroid C	Fibroblast-
0	Nppa	Nr2f1	Igfbp5	Kdr	Cdh5	Upk3b	Hbb-y	Col1a1
1	Sln	Bex4	Atp1b1	Hapln1	Cd93	Smim1	Hbb-bh1	Col1a2
2	Ckm	Igf2r	Rspo3	Lsm2	Igfbp4	Loxl1	Hba-x	Col3a1
3	Gpx3	Cav1	Slc2a1	Nhp2	Ecscr	Rspo1	Hba-a2	Marcks
4	Myl1	Ttn	Sorbs2	Prelid1	Fabp5	Lrrn4	Hmgb2	Postn
5	Pgam2	Kcnq1ot1	Nid2	Mest	Igf2	Gpc3	Crabp1	Igfbp4
6	Cox6a2	Ppp1r1a	Bnip3	Cdh5	Pecam1	S100a11	Crabp2	Loxl1
7	Eno3	Myl1	Ttn	Emcn	Icam2	Myrf	Gypa	Fstl1
8	Ldhb	Ldb3	Cdh2	Plxnd1	Emcn	Col1a2	Nhp2	B2m
9	Ankrd1	Igf2	Rbm24	Plvap	Mmrn2	Cdkn1c	Alas2	Col5a1
10	Mlf1	Igfbp5	Eid1	Igfbp4	Gng11	Aprt	Mt2	S100a11
11	Acaa2	Slc2a1	Synpo2l	Gm8186	Ebf1	S100a10	Lsm2	Fn1
12	Mybphl	Rbm24	Casq1	Gng11	Tie1	Dlk1	Fabp5	Igfbp7
13	Dcn	Casq1	Clu	Hmga1	Plxnd1	Col1a1	Gm10076	Egr1
14	Tcap	Synpo2l	Ldb3	Cldn5	Marcks	Krt7	Dut	Ltbp4
15	Slc22a1	D830030K2	Igf2r	Ecscr	Mest	Ezr	Uba52	Ebf1
16	Smpx	Plekha7	Slc16a3	Marcks	Cdkn1c	Cdh11	Cenpa	S100a10
17	Atp2a2	Tnnt1	Klhdc8b	Uba52	Fn1	Fn1	Prelid1	Morf4l2
18	Fgf12	Rbpms2	Ppp1r1a	Smagp	Kdr	Aldh1a2	Smim1	Ifitm2
19	Acadl	Cacna2d2	Cacna2d2	Gm10076	Col4a1	Golm1	Gm8186	Selm
20	Cox8b	Snhg6	Atp1a1	Tgfb1	Plvap	Tm4sf5	Hmgb3	Gpc3
21	Myoz2	Hbb-bt	Tbx20	H2afy	Smagp	Flrt2	Rpgrip1	Ifitm3
22	Uqcrc1	Add3	Mybpc3	Gnb1	Nrp1	Igf2	Lsm3	Maged2
23	Actn2	Eid1	Ctsb	Xist	Col4a2	Cnpy2	Gm10073	Tpm2
24	Cxcl2	Prrx2	Pygm	Cd93	Flt1	Igfbp5	Wdr89	Mgp
25	Eln	Tbx20	Srl	Hmgb3	Sdpr	Marcks	Siva1	Vcan
26	Igfbp7	Atp1b1	Tagln	Pcbp4	F2r	Sdc4	Hmga1	Cnpy2
27	Epha4	Nudt4	Cox4i2	Siva1	B2m	Colec12	Glrx5	Dcn
28	Ccl4	Hba-a2	Myh7	Lsm3	Klf2	Mgp	Gmnn	Pdlim3
29	Col3a1	Cpne5	Rbpms2	Gm16104	Ifitm3	Siva1	Slc39a8	Junb
30	Fos	Cdc34	Rbm38	Cenpa	Ifitm2	Kcnq1ot1	Mt1	Rtn4
31	F2rl2	Bves	Rtn4	Dut	Cav1	Igfbp4	Gata1	Gnb1
32	Rgs1	Nexn	Hbb-y	Mfng	Klk8	Prr13	Ccna2	Nfib
33	Treml1	Cited1	Ppp1r3c	Cdc34	Tgfb1	Fabp5	Rrm2	Cthrc1
34	Gfi1b	Mest	Nexn	Crabp1	S100a10	Maf	Hmbs	Cd63
35	Gata1	Rbm38	Ctsa	Aprt	Kcnq1ot1	Selm	Ccne1	H2afy
36	Bmp10	Sorbs2	Vcan	Mdfi	Tnfaip1	Snhg6	Xist	Emp1
37	Postn	Gm10073	Tnnt1	Hmgb2	Npr3	Tln1	Gclm	Prelid1
38	Gp9	Pcm1	Bmp2	Traf4	Gnb1	Bex4	Ccnb2	Dpt
39	Ccl6	Ppp1r3c	Cdc34	Rap1b	Emp1	Hbb-y	Rgs10	Rap1b
40	AB124611	Fam96a	Selm	Wdr89	Snhg6	Wdr89	Tomm5	Cyba
41	Steap4	Pkn2	Kcnq1ot1	Flt1	Tln1	B2m	Cdk1	Thbs1
42	F13a1	Fxyd1	Bex4	Ppdpf	Prelid1	Col3a1	Pantr1	Fos
43	Gypa	Cdh2	Fxyd1	Tomm5	Igf2r	Prelid1	Spc25	Eid1

44	Tfap2b	Nid2	Nfib	Col4a1	Rap1b	Gm10073	Cdc34	Ppdpf
45	Pax3	Mtus2	Prelid2	Fam96a	Cdh11	Nfib	Hbb-bt	Ctsb
46	Acvr1c	Hbb-y	Mfge8	Gmnn	Ppdpf	Nrp1	Gadd45gip	Ankrd11
47	Fcgr3	Acta1	Gadd45gip	Gm10073	S100a11	H2afy	Nudt4	Col4a1
48	Pln	Atp1a1	Prelid1	Clec1b	Aprt	Use1	Dbf4	Meox1
49	Gp1bb	Tomm5	Gpx3	Col4a2	Arhgdib	Ankrd11	Top2a	Vapa

Immune Ce	Neural Cre:	PKJ	Platelets	SAN	Smooth Mu	VM	VM-trab	eAM
Fcer1g	Crabp2	Myh7	Pf4	Igfbp5	Rgs5	Myh7	Myl2	Hbb-y
Laptm5	Crabp1	Ttn	Gp9	Cacna2d2	Igfbp7	Ttn	Pln	Bex4
Rac2	Hmga1	Igf2r	Rgs10	Atp1b1	Col4a1	Myl2	Hopx	Hmgb2
Arhgdib	Metrn	Sorbs2	Nrgn	Cpne5	Ebf1	Nexn	Ttn	Hba-a2
B2m	Igdcc3	Atp1b1	Cd9	Bex4	Col4a2	Sorbs2	Uba52	Prelid1
Fcgr3	Pcbp4	Mpped2	Gp1bb	Kcnq1ot1	Mfge8	Hopx	Cd36	Acta2
Spi1	Nr2f1	Synpo2l	Clec1b	Ppp1r1a	Ndufa4l2	Pln	Cox6a2	Cited1
Cyba	Xist	Myl2	Fcer1g	Igf2r	Tpm2	Synpo2l	Myh7	Acta1
Rgs10	Gm8186	Vcan	Arhgdib	Rbpms2	Cox4i2	Ldb3	Acsl1	Gm10073
Ucp2	H2afy	Mybpc3	Plek	Ttn	Gm13889	Cox7a1	Ptgds	Mrpl12
Ctsc	Lsm2	Cdh2	Bin1	Klhdc8b	Gng11	Rbm24	Rbm24	Cdkn1c
Ctsb	Siva1	Ldb3	Treml1	Hbb-y	Pdgfrb	Bves	Hadha	Tagln
Rap1b	Nhp2	Rbm24	Rap1b	Rbm24	Ifitm3	Cdh2	Myom2	Tnnt1
Hexa	Ezh2	Pygm	Tgfb1	Nr2f1	Fn1	Hbb-bs	Etfdh	Dut
Pf4	Hmgb3	Srl	Hbb-y	Slc2a1	Sparcl1	Mt1	Irf2bp2	Ppp1r1a
Maf	Lsm3	Eid1	Cyba	Eid1	Maged2	Ptgds	Pdk4	Sh3bgr
Tgfb1	Prelid1	Kcnq1ot1	Prr13	Ldb3	Col1a2	Mybpc3	Atp2a2	Fam96a
Ifi30	Cdc34	Fxyd1	Tln1	Synpo2l	Cygb	Hbb-bt	Sorbs2	Tceal8
Rnf130	Uba52	Atp1a1	Itga2b	Atp1a1	Fstl1	Igf2r	Mybpc3	Gm10076
Lsp1	Dut	Cited1	Cdkn2d	Cav1	Ltbp4	Fxyd1	Srl	Myl2
AB124611	Gmnn	Igfbp5	Thbs1	Hbb-bt	Colec11	Srl	Cox8b	Tomm5
Irf8	Marcks	Pln	Rasgrp2	Casq1	B2m	Nnt	Acadm	Glrx5
Ctsa	Gm10076	Ppp1r1a	Hist1h1c	Sorbs2	Kcnj8	Nrp1	Pygm	Gadd45gip
Plek	Sox2	Nexn	R3hdm4	Smim1	S100a11	Hba-a2	Acadvl	Hbb-bt
Prr13	Cenpa	Klhdc8b	Capn3	Ppp1r3c	Ifitm2	Nfib	Cacna1c	Cenpa
Tln1	Ppdpf	Hopx	Hexa	Add3	Mylk	Gja1	Slc16a1	Smpx
Junb	Srrm1	Kcnk3	F2rl2	Fam96a	Selm	Pygm	Nnt	Snhg6
Neat1	Rrm2	Cacna1c	Gng11	Hba-a2	Fabp5	Acadm	Ldb3	Apobec2
Psap	Hmgb2	Acadm	Tspo	Rbm38	Marcks	Ndufb4	Synpo2l	Wdr89
Ccl4	Traf4	Txnip	Gclm	Nid2	Rgs4	Casq2	Cdh2	Rbpms2
Ifi27l2a	Cenpf	Ezr	Cela1	Prrx2	Junb	Igf2	Hist1h1c	Eid1
Smagp	Hbb-bh1	Slit2	Tpm2	Bmp2	Postn	Cited1	Nfib	Lsm2
Marcks	Wdr89	Nnt	Smim1	Gadd45gip	Cd63	Atp1b1	Gm17428	Siva1
Ccl6	Gm10073	Irx3	Rbpms2	Cdh2	Fos	Mtus2	Txnip	Myl1
Aprt	Hba-x	Hadha	Gata1	Cox7a1	Arhgdib	Tomm5	Mt1	Prrx2
Fos	Gnb1	Cd36	Clu	Nfib	Klf9	Acta2	Ezr	Rrm2
Ifitm3	Ccna2	Cdc34	Hba-x	Pcm1	Jun	Mrpl12	Casq2	Hba-x
H2afy	Papola	Nfib	Ubash3b	Fxyd1	Rap1b	Ppp1r3c	Mt2	D830030K2
Tgfbi	Msi1	Apobec2	S100a10	Ctsb	Psap	Hadha	Psap	Lsm3
Prelid1	Ccnb2	Rbm38	Rbm38	Tomm5	Rtn4	Dnajc15	Igf2r	Prdx6
F13a1	Top2a	Hmgn3	Tmem158	Nexn	Mgp	Bnip3	Cox7a1	Spc25
Rgs1	Hbb-y	Selm	Laptm5	Egr1	Tln1	Cox8b	Dnajc15	Cox7a1
Tspo	Spc25	Cdkn1c	Gfi1b	Gm10073	Steap4	Pcm1	Nppb	Cdk1
Mt1	Prpf40a	Rbpms2	Hbb-bh1	Mybpc3	Txnip	Glrx5	Bves	Cdc34

Psenen	Ccne1	Cyba	Bcl2l1	Cdc34	Nrp1	Gadd45gip	Mtus2	Cdkn2d
Cxcl2	9-Sep	Ctsb	Esd	Shox2	Col3a1	Cacna1c	Fxyd1	Ndufb4
Fam96a	Cntfr	Psap	Vwf	Tnnt1	Hmgn3	Ezr	Aco2	Ccna2
Use1	Tomm5	Mest	F2r	Tbx20	Acta2	Acsl1	Actn2	Aprt
Bin1	Igfbp4	Bves	Fam96a	Adipor1	Col5a1	Slc16a1	Ech1	S100a11
Scand1	Fabp5	Gja1	Rit1	Hba-x	Serpini1	Mt2	Ckm	Vapa

eVM	eVM-trab
Myl2	Myh7
Hbb-y	Sorbs2
Cited1	Hbb-y
Bex4	Igf2r
Hba-a2	Myl2
Sh3bgr	Cdh2
Acta2	Synpo2l
Gm10073	Bves
Acta1	Kcnq1ot1
Prelid1	Slc2a1
Cdkn1c	Ttn
Ppp1r1a	Bex4
Mrpl12	Rbm24
Gtsf1	Ppp1r1a
Smpx	Tnnt1
Hbb-bt	Atp1b1
Rbpms2	Prelid1
Myh7	Bnip3
Tagln	Rbpms2
Apobec2	Slc16a3
Tnnt1	Hbb-bt
Fam96a	Tomm5
Snhg6	Cdc34
Glrx5	Hmgn3
Hba-x	Snhg6
Tomm5	Igf2
Gm10076	Gadd45gip1
Wdr89	Mpped2
Gadd45gip	Nrp1
Fbxl22	Eid1
Pln	Ldb3
Cox7a1	Siva1
Dut	Gpc3
Eid1	Cdkn1c
Mpped2	Nnt
Cdc34	Prrx2
Cenpa	Nexn
Des	Hopx
Hmgb2	Hba-a2
Hmgn3	Glrx5
Prrx2	Vcan
Myoz2	Mybpc3
Cdkn2d	Gnb1
Lsm3	Sfrp1

Aprt	Gm10073
Prdx6	Wdr89
Adipor1	Dut
Mest	Aprt
Siva1	Rbm38
Gmnn	Mtus2

Table S2 The top 50 genes that contributed most to the classification of each cell type by using SMASH method.

Table S2. Primers used in RT-PCR and genotyping

Gene name	Sequence(5'-3')	Species
$Dbh^{f/f}$	5’-GTCAGGGTTCCCCAGATTAGTTTC-3’	Mus musculus
	5’-CCATTTATAGATCCAACCACTGGG-3’	Mus musculus
Rosa26-LSL-Tdtomato	GGC ATT AAA GCA GCG TAT CC	Mus musculus
	CTG TTC CTG TAC GGC ATG G	Mus musculus
Myh6-Cre	GCG GTC TGG CAG TAA AAA CTA TC	Mus musculus
	GTG AAA CAG CAT TGC TGT CAC TT	Mus musculus
Dbh-CFP	ATCCCTCAACCCTGGGAACT	Mus musculus
	TCTGCATGGAAGACAGCAGG	Mus musculus
Dbh-CreERT	GCCCCGCTAACCCCACTG	Mus musculus
	GCCCGGACCGACGATGAAGC	Mus musculus
Dbh-Cre	ATCCCTCAACCCTGGGAACT	Mus musculus
	TCTGCATGGAAGACAGCAGG	Mus musculus
Cx40-ERT	CAGCCTCTAGAAAGTAGAGGG	Mus musculus
	GCATCGACCGGTAATGCAGGC	Mus musculus
Cx40-EGFP	CTCCAATTA ACTCCTTGTGAGCC	Mus musculus
	AGGCTGAATGGTATCGCACC	Mus musculus
tdTomato-ChR2	CTG TTC CTG TAC GGC ATG G	Mus musculus
	GGC ATT AAA GCA GCG TAT CC	Mus musculus

Table S3 The Primers used for genotyping all mouse model shown in this paper
