# Supplementary material for: Dbh+ catecholaminergic cardiomyocytes contribute to the structure and function of the cardiac conduction system in murine heart
Source: Nat Commun. 2023 Nov 28;14:7801. doi: 10.1038/s41467-023-42658-9 (PMC10684617; doi:10.1038/s41467-023-42658-9)
Supplement: Supplementary file 3 — Description of Additional Supplementary Files [file 41467_2023_42658_MOESM3_ESM.pdf]

Description of Additional Supplementary Files

Supplementary Data 1: (Related to Figure 1): A 3D interactive plot of UMAP dimensions from cell types identified in our original whole embryo (E8.5, E10.5) and whole heart (E12.5, E14.5, E16.5, P3) scRNAseq post-quality control. Cells are coloured by cell identity. Hovering over a cell produces a readout of the stage of a cell's identity, stage, nUMI, nGene, Ratio of mtUMI/nUMI, and its position in 3D space.

Supplementary Data 2: (Related to Figure 1): A 3D interactive plot of PHATE dimensions from cells identified within our cardiomyocyte-lineage. Cells are coloured by cell identity. Hovering over a cell produces a readout of the stage of a cell's identity, stage, nUMI, nGene, Ratio of mtUMI/nUMI, and its position in 3D space.

Supplementary Data 3: (Related to Figure 1): A 3D interactive plot of PHATE dimensions from Dbh+ cells identified within our cardiomyocyte-lineage. Cells are coloured by cell identity. Hovering over a cell produces a readout of the stage of a cell's identity, stage, nUMI, nGene, Ratio of mtUMI/nUMI, and its position in 3D space

Supplementary Movie 1: (Related to Figure 6): 3D reconstruction of Dd+-CMs in P3 murine heart

Supplementary Movie 2: (Related to Figure 6): 3D reconstruction of Dd+-CMs in adult murine heart
