# Supplementary material for: Dbh+ catecholaminergic cardiomyocytes contribute to the structure and function of the cardiac conduction system in murine heart
Source: Nat Commun. 2023 Nov 28;14:7801. doi: 10.1038/s41467-023-42658-9 (PMC10684617; doi:10.1038/s41467-023-42658-9)
Supplement: Supplementary file 9 — Reporting Summary [file 41467_2023_42658_MOESM9_ESM.pdf]

nature portfolio

Corresponding author(s): Ming Lei

Last updated by author(s): 03 Oct, 2023

Reporting Summary

Nature Portfolio wishes to improve the reproducibility of the work that we publish. This form provides structure for consistency and transparency in reporting. For further information on Nature Portfolio policies, see our Editorial Policies and the Editorial Policy Checklist.

Statistics

For all statistical analyses, confirm that the following items are present in the figure legend, table legend, main text, or Methods section.

n/a	Confirmed	
[ ]	[x]	The exact sample size (*n*) for each experimental group/condition, given as a discrete number and unit of measurement
[ ]	[x]	A statement on whether measurements were taken from distinct samples or whether the same sample was measured repeatedly
[ ]	[x]	The statistical test(s) used AND whether they are one- or two-sided<br>*Only common tests should be described solely by name; describe more complex techniques in the Methods section.*
[x]	[ ]	A description of all covariates tested
[x]	[ ]	A description of any assumptions or corrections, such as tests of normality and adjustment for multiple comparisons
[ ]	[x]	A full description of the statistical parameters including central tendency (e.g. means) or other basic estimates (e.g. regression coefficient) AND variation (e.g. standard deviation) or associated estimates of uncertainty (e.g. confidence intervals)
[ ]	[x]	For null hypothesis testing, the test statistic (e.g. *F*, *t*, *r*) with confidence intervals, effect sizes, degrees of freedom and *P* value noted<br>*Give P values as exact values whenever suitable.*
[x]	[ ]	For Bayesian analysis, information on the choice of priors and Markov chain Monte Carlo settings
[x]	[ ]	For hierarchical and complex designs, identification of the appropriate level for tests and full reporting of outcomes
[x]	[ ]	Estimates of effect sizes (e.g. Cohen's *d*, Pearson's *r*), indicating how they were calculated

Our web collection on statistics for biologists contains articles on many of the points above.

Software and code

Policy information about availability of computer code

Data collection

FastQC v.0.12.2
Trimmomatic v 0.36
Seurat v3.1.0
edgeR v3.18.1
clusterProfiler v. 3.4.4
Cell Ranger v3.0

Data analysis

R analysis:
R v4.0
package * version date lib source
abind 1.4-5 2016-07-21 [1] CRAN (R 4.0.3)
AnnotationDbi * 1.52.0 2020-10-27 [1] Bioconductor
AnnotationForge 1.32.0 2020-10-27 [1] Bioconductor
askpass 1.1 2019-01-13 [1] CRAN (R 4.0.4)
assertthat 0.2.1 2019-03-21 [1] CRAN (R 4.0.4)
backports 1.2.1 2020-12-09 [1] CRAN (R 4.0.3)
Biobase * 2.50.0 2020-10-27 [1] Bioconductor
BiocFileCache 1.14.0 2020-10-27 [1] Bioconductor
BiocGenerics * 0.36.1 2021-04-16 [1] Bioconductor

```
BiocManager     * 1.30.16  2021-06-15 [1] CRAN (R 4.0.5)
BiocParallel    * 1.24.1   2020-11-06 [1] Bioconductor
biomaRt         * 2.46.3   2021-02-09 [1] Bioconductor
bit               4.0.4    2020-08-04 [1] CRAN (R 4.0.4)
bit64             4.0.5    2020-08-30 [1] CRAN (R 4.0.4)
bitops            1.0-6    2013-08-17 [1] CRAN (R 4.0.3)
blob              1.2.2    2021-07-23 [1] CRAN (R 4.0.5)
broom             0.7.9    2021-07-27 [1] CRAN (R 4.0.5)
cachem            1.0.4    2021-02-13 [1] CRAN (R 4.0.4)
Cairo             1.5-12.2 2020-07-07 [1] CRAN (R 4.0.3)
callr             3.7.0    2021-04-20 [1] CRAN (R 4.0.5)
cellranger        1.1.0    2016-07-27 [1] CRAN (R 4.0.4)
circlize        * 0.4.13   2021-06-09 [1] CRAN (R 4.0.5)
cli               3.0.1    2021-07-17 [1] CRAN (R 4.0.5)
clue              0.3-59   2021-04-16 [1] CRAN (R 4.0.5)
cluster           2.1.0    2019-06-19 [2] CRAN (R 4.0.4)
clusterProfiler * 3.18.1   2021-02-09 [1] Bioconductor
codetools         0.2-18   2020-11-04 [2] CRAN (R 4.0.4)
colorspace        2.0-0    2020-11-11 [1] CRAN (R 4.0.4)
ComplexHeatmap  * 2.6.2    2020-11-12 [1] Bioconductor
cowplot           1.1.1    2020-12-30 [1] CRAN (R 4.0.4)
crayon            1.4.1    2021-02-08 [1] CRAN (R 4.0.4)
curl              4.3.2    2021-06-23 [1] CRAN (R 4.0.5)
data.table        1.14.2   2021-09-27 [1] CRAN (R 4.0.5)
DBI               1.1.1    2021-01-15 [1] CRAN (R 4.0.4)
dbplyr            2.1.1    2021-04-06 [1] CRAN (R 4.0.5)
deldir            1.0-5    2021-10-09 [1] CRAN (R 4.0.5)
dendextend        1.15.1   2021-05-08 [1] CRAN (R 4.0.5)
desc              1.4.0    2021-09-28 [1] CRAN (R 4.0.5)
devtools        * 2.4.2    2021-06-07 [1] CRAN (R 4.0.4)
DiagrammeR        1.0.6.1  2020-05-08 [1] CRAN (R 4.0.4)
digest            0.6.27   2020-10-24 [1] CRAN (R 4.0.4)
DO.db             2.9      2021-04-17 [1] Bioconductor
DOSE              3.16.0   2020-10-27 [1] Bioconductor
downloader        0.4      2015-07-09 [1] CRAN (R 4.0.4)
dplyr           * 1.0.6    2021-05-05 [1] CRAN (R 4.0.5)
DT                0.19     2021-09-02 [1] CRAN (R 4.0.5)
dynamicTreeCut    1.63-1   2016-03-11 [1] CRAN (R 4.0.3)
ellipsis          0.3.2    2021-04-29 [1] CRAN (R 4.0.5)
enrichplot        1.10.2   2021-01-28 [1] Bioconductor
fansi             0.4.2    2021-01-15 [1] CRAN (R 4.0.4)
farver            2.1.0    2021-02-28 [1] CRAN (R 4.0.4)
fastmap           1.1.0    2021-01-25 [1] CRAN (R 4.0.4)
fastmatch         1.1-3    2021-07-23 [1] CRAN (R 4.0.5)
fgsea             1.16.0   2020-10-27 [1] Bioconductor
fitdistrplus      1.1-6    2021-09-28 [1] CRAN (R 4.0.5)
forcats         * 0.5.1    2021-01-27 [1] CRAN (R 4.0.5)
foreach           1.5.1    2020-10-15 [1] CRAN (R 4.0.4)
fs                1.5.0    2020-07-31 [1] CRAN (R 4.0.4)
future          * 1.22.1   2021-08-25 [1] CRAN (R 4.0.5)
future.apply      1.8.1    2021-08-10 [1] CRAN (R 4.0.4)
generics          0.1.0    2020-10-31 [1] CRAN (R 4.0.4)
GetoptLong        1.0.5    2020-12-15 [1] CRAN (R 4.0.4)
ggforce           0.3.3    2021-03-05 [1] CRAN (R 4.0.5)
ggfun             0.0.4    2021-09-17 [1] CRAN (R 4.0.5)
ggplot2         * 3.3.5    2021-06-25 [1] CRAN (R 4.0.5)
ggraph            2.0.5    2021-02-23 [1] CRAN (R 4.0.5)
ggrepel         * 0.9.1    2021-01-15 [1] CRAN (R 4.0.4)
ggridges          0.5.3    2021-01-08 [1] CRAN (R 4.0.4)
GlobalOptions     0.1.2    2020-06-10 [1] CRAN (R 4.0.4)
globals           0.14.0   2020-11-22 [1] CRAN (R 4.0.3)
glue              1.4.2    2020-08-27 [1] CRAN (R 4.0.4)
GO.db           * 3.12.1   2021-03-26 [1] Bioconductor
goftest           1.2-3    2021-10-07 [1] CRAN (R 4.0.5)
GOSemSim          2.16.1   2020-10-29 [1] Bioconductor
graph           * 1.68.0   2020-10-27 [1] Bioconductor
graphlayouts      0.7.1    2020-10-26 [1] CRAN (R 4.0.5)
gridExtra         2.3      2017-09-09 [1] CRAN (R 4.0.4)
```

```
gtable          0.3.0    2019-03-25 [1] CRAN (R 4.0.4)
haven           2.4.3    2021-08-04 [1] CRAN (R 4.0.5)
heatmaply       1.3.0    2021-10-09 [1] CRAN (R 4.0.4)
hms             1.1.1    2021-09-26 [1] CRAN (R 4.0.5)
htmltools       0.5.2    2021-08-25 [1] CRAN (R 4.0.5)
htmlwidgets   * 1.5.4    2021-09-08 [1] CRAN (R 4.0.5)
httpuv          1.6.3    2021-09-09 [1] CRAN (R 4.0.5)
httr            1.4.2    2020-07-20 [1] CRAN (R 4.0.4)
ica             1.0-2    2018-05-24 [1] CRAN (R 4.0.3)
igraph          1.2.6    2020-10-06 [1] CRAN (R 4.0.5)
IRanges       * 2.24.1   2020-12-12 [1] Bioconductor
irlba           2.3.3    2019-02-05 [1] CRAN (R 4.0.4)
iterators       1.0.13   2020-10-15 [1] CRAN (R 4.0.4)
jsonlite        1.7.2    2020-12-09 [1] CRAN (R 4.0.4)
KernSmooth      2.23-18  2020-10-29 [2] CRAN (R 4.0.4)
later           1.3.0    2021-08-18 [1] CRAN (R 4.0.5)
lattice         0.20-41  2020-04-02 [2] CRAN (R 4.0.4)
lazyeval        0.2.2    2019-03-15 [1] CRAN (R 4.0.4)
leiden          0.3.9    2021-07-27 [1] CRAN (R 4.0.5)
lifecycle       1.0.1    2021-09-24 [1] CRAN (R 4.0.5)
listenv         0.8.0    2019-12-05 [1] CRAN (R 4.0.4)
lmtest          0.9-38   2020-09-09 [1] CRAN (R 4.0.4)
lubridate       1.7.10   2021-02-26 [1] CRAN (R 4.0.5)
magrittr        2.0.1    2020-11-17 [1] CRAN (R 4.0.4)
MASS          * 7.3-53   2020-09-09 [2] CRAN (R 4.0.4)
Matrix        * 1.3-4    2021-06-01 [1] CRAN (R 4.0.5)
matrixStats     0.58.0   2021-01-29 [1] CRAN (R 4.0.4)
memoise         2.0.0    2021-01-26 [1] CRAN (R 4.0.4)
mgcv            1.8-33   2020-08-27 [2] CRAN (R 4.0.4)
mime            0.12     2021-09-28 [1] CRAN (R 4.0.5)
miniUI          0.1.1.1  2018-05-18 [1] CRAN (R 4.0.4)
modelr          0.1.8    2020-05-19 [1] CRAN (R 4.0.4)
munsell         0.5.0    2018-06-12 [1] CRAN (R 4.0.4)
nlme            3.1-152  2021-02-04 [2] CRAN (R 4.0.4)
openssl         1.4.5    2021-09-02 [1] CRAN (R 4.0.5)
org.Mm.eg.db  * 3.12.0   2021-04-17 [1] Bioconductor
parallelly      1.28.1   2021-09-09 [1] CRAN (R 4.0.5)
patchwork       1.1.1    2020-12-17 [1] CRAN (R 4.0.4)
pbapply         1.5-0    2021-09-16 [1] CRAN (R 4.0.5)
phateR        * 1.0.7    2021-02-12 [1] CRAN (R 4.0.5)
pillar          1.6.3    2021-09-26 [1] CRAN (R 4.0.5)
pkgbuild        1.2.0    2020-12-15 [1] CRAN (R 4.0.5)
pkgconfig       2.0.3    2019-09-22 [1] CRAN (R 4.0.4)
pkgload         1.2.3    2021-10-13 [1] CRAN (R 4.0.4)
plotly        * 4.10.0   2021-10-09 [1] CRAN (R 4.0.4)
plyr            1.8.6    2020-03-03 [1] CRAN (R 4.0.4)
png             0.1-7    2013-12-03 [1] CRAN (R 4.0.3)
polyclip        1.10-0   2019-03-14 [1] CRAN (R 4.0.3)
prettyunits     1.1.1    2020-01-24 [1] CRAN (R 4.0.4)
processx        3.5.2    2021-04-30 [1] CRAN (R 4.0.5)
progress        1.2.2    2019-05-16 [1] CRAN (R 4.0.4)
promises        1.2.0.1  2021-02-11 [1] CRAN (R 4.0.4)
ps              1.6.0    2021-02-28 [1] CRAN (R 4.0.4)
purrr         * 0.3.4    2020-04-17 [1] CRAN (R 4.0.4)
qvalue          2.22.0   2020-10-27 [1] Bioconductor
R.methodsS3   * 1.8.1    2020-08-26 [1] CRAN (R 4.0.3)
R.oo          * 1.24.0   2020-08-26 [1] CRAN (R 4.0.3)
R.utils       * 2.11.0   2021-09-26 [1] CRAN (R 4.0.5)
R6              2.5.1    2021-08-19 [1] CRAN (R 4.0.5)
RANN            2.6.1    2019-01-08 [1] CRAN (R 4.0.4)
rappdirs        0.3.3    2021-01-31 [1] CRAN (R 4.0.4)
RColorBrewer  * 1.1-2    2014-12-07 [1] CRAN (R 4.0.3)
Rcpp            1.0.7    2021-07-07 [1] CRAN (R 4.0.5)
RcppAnnoy       0.0.19   2021-07-30 [1] CRAN (R 4.0.5)
RCurl           1.98-1.3 2021-03-16 [1] CRAN (R 4.0.4)
readr         * 2.0.2    2021-09-27 [1] CRAN (R 4.0.5)
readxl          1.3.1    2019-03-13 [1] CRAN (R 4.0.4)
registry        0.5-1    2019-03-05 [1] CRAN (R 4.0.3)
```

```
remotes          2.4.1    2021-09-29 [1] CRAN (R 4.0.5)
reprex           2.0.1    2021-08-05 [1] CRAN (R 4.0.4)
reshape2         1.4.4    2020-04-09 [1] CRAN (R 4.0.4)
reticulate     * 1.22     2021-09-17 [1] CRAN (R 4.0.5)
rjson            0.2.20   2018-06-08 [1] CRAN (R 4.0.3)
rlang            0.4.10   2020-12-30 [1] CRAN (R 4.0.4)
ROCR             1.0-11   2020-05-02 [1] CRAN (R 4.0.4)
rpart            4.1-15   2019-04-12 [2] CRAN (R 4.0.4)
rprojroot        2.0.2    2020-11-15 [1] CRAN (R 4.0.5)
RSQLite          2.2.7    2021-04-22 [1] CRAN (R 4.0.5)
rstudioapi       0.13     2020-11-12 [1] CRAN (R 4.0.4)
Rtsne            0.15     2018-11-10 [1] CRAN (R 4.0.4)
rvcheck          0.1.5    2019-10-01 [1] CRAN (R 4.0.4)
rvest            1.0.1    2021-07-26 [1] CRAN (R 4.0.5)
S4Vectors      * 0.28.1   2020-12-09 [1] Bioconductor
scales           1.1.1    2020-05-11 [1] CRAN (R 4.0.4)
scattermore      0.7      2020-11-24 [1] CRAN (R 4.0.4)
scatterpie       0.1.7    2021-08-20 [1] CRAN (R 4.0.5)
sctransform    * 0.3.2    2020-12-16 [1] CRAN (R 4.0.4)
see            * 0.6.8    2021-10-03 [1] CRAN (R 4.0.4)
seriation        1.3.0    2021-06-30 [1] CRAN (R 4.0.5)
sessioninfo      1.1.1    2018-11-05 [1] CRAN (R 4.0.5)
Seurat         * 4.0.2    2021-05-20 [1] CRAN (R 4.0.5)
SeuratObject   * 4.0.2    2021-06-09 [1] CRAN (R 4.0.5)
shadowtext       0.0.9    2021-09-19 [1] CRAN (R 4.0.5)
shape            1.4.6    2021-05-19 [1] CRAN (R 4.0.5)
shiny            1.7.1    2021-10-02 [1] CRAN (R 4.0.5)
SparseM        * 1.81     2021-02-18 [1] CRAN (R 4.0.4)
spatstat.core    2.3-0    2021-07-16 [1] CRAN (R 4.0.5)
spatstat.data    2.1-0    2021-03-21 [1] CRAN (R 4.0.4)
spatstat.geom    2.3-0    2021-10-09 [1] CRAN (R 4.0.5)
spatstat.sparse  2.0-0    2021-03-16 [1] CRAN (R 4.0.5)
spatstat.utils   2.2-0    2021-06-14 [1] CRAN (R 4.0.5)
stringi          1.5.3    2020-09-09 [1] CRAN (R 4.0.3)
stringr        * 1.4.0    2019-02-10 [1] CRAN (R 4.0.4)
survival         3.2-7    2020-09-28 [2] CRAN (R 4.0.4)
tensor           1.5      2012-05-05 [1] CRAN (R 4.0.3)
testthat         3.1.0    2021-10-04 [1] CRAN (R 4.0.5)
tibble         * 3.1.2    2021-05-16 [1] CRAN (R 4.0.5)
tidygraph        1.2.0    2020-05-12 [1] CRAN (R 4.0.5)
tidyr          * 1.1.3    2021-03-03 [1] CRAN (R 4.0.4)
tidyselect       1.1.1    2021-04-30 [1] CRAN (R 4.0.5)
tidyverse      * 1.3.1    2021-04-15 [1] CRAN (R 4.0.5)
topGO          * 2.42.0   2020-10-31 [1] Bioconductor
TSP              1.1-10   2020-04-17 [1] CRAN (R 4.0.5)
tweenr           1.0.2    2021-03-23 [1] CRAN (R 4.0.5)
tzdb             0.1.2    2021-07-20 [1] CRAN (R 4.0.5)
UpSetR           1.4.0    2019-05-22 [1] CRAN (R 4.0.4)
usethis        * 2.0.1    2021-02-10 [1] CRAN (R 4.0.5)
utf8             1.2.1    2021-03-12 [1] CRAN (R 4.0.4)
uwot             0.1.10   2020-12-15 [1] CRAN (R 4.0.4)
vctrs            0.3.8    2021-04-29 [1] CRAN (R 4.0.5)
viridis        * 0.6.2    2021-10-13 [1] CRAN (R 4.0.4)
viridisLite    * 0.4.0    2021-04-13 [1] CRAN (R 4.0.5)
ViSEAGO        * 1.4.0    2020-10-28 [1] forgemia.inra.fr, bioconductor.org (R 4.0.3)
visNetwork       2.1.0    2021-09-29 [1] CRAN (R 4.0.4)
webshot          0.5.2    2019-11-22 [1] CRAN (R 4.0.4)
withr            2.4.2    2021-04-18 [1] CRAN (R 4.0.5)
XML              3.99-0.6 2021-03-16 [1] CRAN (R 4.0.4)
xml2             1.3.2    2020-04-23 [1] CRAN (R 4.0.4)
xtable           1.8-4    2019-04-21 [1] CRAN (R 4.0.4)
zoo              1.8-9    2021-03-09 [1] CRAN (R 4.0.4)
```
Seurat.multicore https://github.com/vertesy/Seurat.multicore

Python analysis:
anaconda v3
conda v4.10.3
python 3.6

absl-py	0.13.0
alabaster	0.7.12
anndata	0.7.6
appdirs	1.4.4
argh	0.26.2
arrow	1.2.0
astroid	2.6.6
astunparse	1.6.3
async-generator	1.10
atomicwrites	1.4.0
attrs	21.2.0
autopep8	1.5.7
Babel	2.9.1
backcall	0.2.0
bcrypt	3.2.0
binaryornot	0.4.4
black	21.9b0
bleach	4.1.0
brotlipy	0.7.0
cached-property	1.5.2
cachetools	4.2.2
certifi	2021.5.30
cffi	1.14.6
chardet	4.0.0
charset-normalizer	2.0.4
clang	5.0
click	8.0.1
cloudpickle	1.6.0
colorama	0.4.4
cookiecutter	1.7.3
cryptography	35.0.0
cycler	0.10.0
dataclasses	0.8
datatable	1.0.0
decorator	4.4.2
defusedxml	0.7.1
diff-match-patch	20200713
docutils	0.17.1
entrypoints	0.3
flake8	3.9.2
flatbuffers	1.12
future	0.18.2
gast	0.4.0
get-version	2.1
google-auth	1.35.0
google-auth-oauthlib	0.4.5
google-pasta	0.2.0
grpcio	1.39.0
h5py	2.10.0
harmonypy	0.0.5
idna	3.2
imagesize	1.2.0
imbalanced-learn	0.8.0
importlib-metadata	3.10.0
inflection	0.5.1
intervaltree	3.1.0
ipykernel	5.5.6
ipython	7.16.1
ipython-genutils	0.2.0
isort	5.9.3
jedi	0.18.0
Jinja2	2.11.3
jinja2-time	0.2.0
joblib	1.0.1
jsonschema	3.2.0
jupyter-client	6.1.12
jupyter-core	4.8.1

jupyterlab-pygments	0.1.2
keras	2.6.0
Keras-Preprocessing	1.1.2
keyring	23.2.1
kiwisolver	1.3.1
lazy-object-proxy	1.6.0
legacy-api-wrap	1.2
llvmlite	0.36.0
Markdown	3.3.4
MarkupSafe	1.1.1
matplotlib	3.3.4
mccabe	0.6.1
mistune	0.8.4
mkl-fft	1.3.0
mkl-random	1.0.4
mkl-service	2.3.0
mock	4.0.3
mypy-extensions	0.4.3
natsort	7.1.1
nbclient	0.5.4
nbconvert	6.0.7
nbformat	5.1.3
nest-asyncio	1.5.1
networkx	2.5.1
numba	0.53.1
numexpr	2.7.3
numpy	1.19.5
numpydoc	1.1.0
oauthlib	3.1.1
opt-einsum	3.3.0
packaging	21.0
pandas	1.1.5
pandocfilters	1.5.0
paramiko	2.8.0
parso	0.8.2
pathspec	0.9.0
patsy	0.5.1
pexpect	4.8.0
pickleshare	0.7.5
Pillow	8.3.1
pip	21.0.1
platformdirs	2.4.0
plotly	5.3.0
pluggy	1.0.0
poyo	0.5.0
prompt-toolkit	3.0.20
protobuf	3.17.3
psutil	5.8.0
ptyprocess	0.7.0
pyasn1	0.4.8
pyasn1-modules	0.2.8
pycodestyle	2.7.0
pycparser	2.20
pydocstyle	6.1.1
pyflakes	2.3.1
Pygments	2.10.0
pylint	2.9.6
pyls-black	0.4.6
pyls-spyder	0.4.0
PyNaCl	1.4.0
pynndescent	0.5.4
pyOpenSSL	20.0.1
pyparsing	2.4.7
PyQt5	5.12.3
PyQt5-sip	12.9.0
PyQtWebEngine	5.12.1
pyrsistent	0.18.0
PySocks	1.7.1

python-dateutil	2.8.2
python-jsonrpc-server	0.4.0
python-language-server	0.36.2
python-lsp-black	1.0.0
python-lsp-jsonrpc	1.0.0
python-lsp-server	1.2.4
python-slugify	5.0.2
pytz	2021.1
pywin32	302
pywin32-ctypes	0.2.0
PyYAML	5.4.1
pyzmq	22.3.0
QDarkStyle	3.0.2
qstylizer	0.2.1
QtAwesome	1.0.3
qtconsole	5.1.1
QtPy	1.11.2
regex	2021.10.8
requests	2.26.0
requests-oauthlib	1.3.0
rope	0.21.0
rsa	4.7.2
Rtree	0.9.7
scanpy	1.7.2
scikit-learn	0.24.2
scipy	1.5.4
seaborn	0.11.2
setuptools	52.0.0.post20210125
shap	0.39.0
sinfo	0.3.4
six	1.16.0
slicer	0.0.7
smashpy	0.1.1
snowballstemmer	2.1.0
sortedcontainers	2.4.0
Sphinx	4.2.0
sphinxcontrib-applehelp	1.0.2
sphinxcontrib-devhelp	1.0.2
sphinxcontrib-htmlhelp	2.0.0
sphinxcontrib-jsmath	1.0.1
sphinxcontrib-qthelp	1.0.3
sphinxcontrib-serializinghtml	1.1.5
spyder	5.1.5
spyder-kernels	2.1.3
statsmodels	0.12.2
stdlib-list	0.8.0
tables	3.6.1
tenacity	8.0.1
tensorboard	2.6.0
tensorboard-data-server	0.6.1
tensorboard-plugin-wit	1.8.0
tensorflow	2.6.0
tensorflow-estimator	2.6.0
termcolor	1.1.0
testpath	0.5.0
text-unidecode	1.3
textdistance	4.2.1
threadpoolctl	2.2.0
three-merge	0.1.1
tinycss	0.4
tinycss2	1.1.0
toml	0.10.2
tomli	1.2.1
tornado	6.1
tqdm	4.62.2
traitlets	4.3.3
typed-ast	1.4.3
typing-extensions	3.7.4.3

ujson	4.2.0
umap-learn	0.5.1
Unidecode	1.2.0
urllib3	1.26.6
watchdog	2.1.6
wcwidth	0.2.5
webencodings	0.5.1
Werkzeug	2.0.1
wheel	0.37.0
whichcraft	0.6.1
win-inet-pton	1.1.0
wincertstore	0.2
wrapt	1.12.1
xgboost	1.4.2
xlrd	1.2.0
yapf	0.31.0
zipp	3.5.0

For manuscripts utilizing custom algorithms or software that are central to the research but not yet described in published literature, software must be made available to editors and reviewers. We strongly encourage code deposition in a community repository (e.g. GitHub). See the Nature Portfolio guidelines for submitting code & software for further information.

Data

Policy information about availability of data

All manuscripts must include a data availability statement. This statement should provide the following information, where applicable:

- Accession codes, unique identifiers, or web links for publicly available datasets
- A description of any restrictions on data availability
- For clinical datasets or third party data, please ensure that the statement adheres to our policy

Raw sequencing data generated by Stereo-seq and nucleic acid dye staining images have been deposited to Spatial Transcript Omics DataBase (STOmics DB) with the accession number STT0000009. The raw data and processed data of single cell RNA sequence have been deposited into CNGB Sequence Archive of China National GeneBank DataBase with accession number CNP0002316. Representative code is available on github (https://github.com/Lei-group/Dbh_paper)

Research involving human participants, their data, or biological material

Policy information about studies with human participants or human data. See also policy information about sex, gender (identity/presentation), and sexual orientation and race, ethnicity and racism.

Reporting on sex and gender	not applicable
Reporting on race, ethnicity, or other socially relevant groupings	not applicable
Population characteristics	not applicable
Recruitment	not applicable
Ethics oversight	not applicable

Note that full information on the approval of the study protocol must also be provided in the manuscript.

Field-specific reporting

Please select the one below that is the best fit for your research. If you are not sure, read the appropriate sections before making your selection.

☒ Life sciences ☐ Behavioural & social sciences ☐ Ecological, evolutionary & environmental sciences

For a reference copy of the document with all sections, see nature.com/documents/nr-reporting-summary-flat.pdf

Life sciences study design

All studies must disclose on these points even when the disclosure is negative.

Sample size	Sample size:<br>The sample size for each experiment is determined by the nature of the experiment, acceptable level of significance and power of the study. For example, adequately powered scRNAseq requires a significant number of cells given the inherent noise associated with read dropout

across cells. However, the sample size for the physiological and imaging experiments is smaller, given larger expected effect sizes and reduced variance. The details of sample size for each experiment are listed below:
ScRNAseq: Whole embryos (E8.5, E10.5, n=10 embryos per stage) or isolated hearts (E12.5, E14.5, P3, n= 10 hearts per stage) giving ~450,000 cells before cellwise quality control with at least 20,000 cells per stage. Post quality-control we had cell n =175237.

SrT: at least n = 3 sections for embryonic hearts from DbhCre/R26-tdTomato mice at E12.5, E14.5, and P3.

Lineage tracing experiments, Whole embryos (E8.5, E9.5, E10.5, E12.5, E14.5, n=5 embryos per stage) or isolated hearts (E12.5, E13.5, E14.5, E16.5, P3, n=5 hearts per stage)
Optogenetic electrophysiology studies: comparing photostimulation-induced electrophysiological characteristics of DbhCre/ChR2-tdTomato hearts with Cx40-CreERT/ChR2-tdTomato and MHC-Cre/ChR2-tdTomato and DbhCKO hearts.characterised and compared the RV effective refractory periods (ERPs) determined by RV epicardial optical programmed pacing S1S2 protocol selectively photostimulation of Dbh-ChR2, Cx40-ChR2 and MHC-ChR2 expressing cells in these models (n=5-8 per group per experiments)

Data exclusions

We filtered our original ~450,000 cells by the quality control metrics described in the methods. From these post-quality control datasets we then selected cardiomyocyte lineage cell types for downstream analysis as described in the manuscript. During analysis of the cardiomyocyte lineage, a small cluster of 'cells' were excluded from presentation of analysis results on the basis that their transcriptional signatures were not consistent with any clear biological cell type, but more likely reflected either background cell-free floating RNA droplets, or extremely stressed cells. However, these cells were included in the normalisation of the dataset via SCTransform, so this lack of presentation should not bias the results from other cell types.

Replication

Each of the developmental embryos or heart tissues were collected at unique time points, i.e. E8.5, E10.5, E12.5, E14.5 and P3. Samples are considered biological replicates for the purpose of SrT and scRNAseq. Consecutive tissue sections from the same heart tissue were considered technical replicates in the SrT (Figure 2C, Figure 3C) and RNAscope and immunohistology (Figure 3B,C; Figure 4, Figures S4, S5) experiments. However, it is important to notice that consecutive sections are highly similar but not identical.

Randomization

Not performed since all groups received the same conditions, within the remit of control or experimental conditions. There was no differential treatment between groups on the basis of genotypes or other biological distinctions, and thus randomisation was deemed to be unnecessary.

Blinding

Blinding was not performed. Initial scRNAseq interrogation was hypothesis generating, and so blinding would have been mostly meaningless. For further experimentation, given the expected binary-like differences between Dbh-promoter based constructs, such as in Dbh-ChR2 in optical mapping, it was deemed that the experimenter would have been unblinded rapidly upon experiment start, which could have then lead to possible overconfidence in the effect of blinding. Thus, blinding was deemed regretably not possible.

Reporting for specific materials, systems and methods

We require information from authors about some types of materials, experimental systems and methods used in many studies. Here, indicate whether each material, system or method listed is relevant to your study. If you are not sure if a list item applies to your research, read the appropriate section before selecting a response.

Materials & experimental systems

n/a	Involved in the study
☐	☒ Antibodies
☒	☐ Eukaryotic cell lines
☒	☐ Palaeontology and archaeology
☐	☒ Animals and other organisms
☒	☐ Clinical data
☒	☐ Dual use research of concern
☒	☐ Plants

Methods

n/a	Involved in the study
☒	☐ ChIP-seq
☒	☐ Flow cytometry
☒	☐ MRI-based neuroimaging

Antibodies

Antibodies used

Anti-α-actinin, Ms, Abcam Ab9465, 1:200
Anti-Th Abcam, Rb, Ab6211, 1:200
Anti-DYKDDDDK Epitope Tag Antibody (L5), Rat, Novus Biologicals, NBP1-06712, 1:50
Anti-CFP Polyclonal antibody, Rb, Biovision, 5986-30T, 1:200
Donkey anti-Mouse 488 Abcam Ab150105, 1:200
Donkey anti-Rat 594, Abcam, Ab150156, 1:200
Donkey anti-Rabbit 594 Abcam ab150076, 1:200
Donkey anti-Rabbit 647 Abcam ab150075, 1:200
Mounting-Media Abcam Ab104139, one drop
Hcn4, Advanced Cell Diagnostics, Inc Cat No. 421271-C2
Dbh, Advanced Cell Diagnostics, Inc Cat No. 407851-C3
Cacna2d2, Advanced Cell Diagnostics, Inc Cat No. 449221-C2
Id2, Advanced Cell Diagnostics, Inc Cat No. 445871-C2

Tbx18, Advanced Cell Diagnostics, Inc Cat No. 515221-C2
Shox2, Advanced Cell Diagnostics, Inc Cat No. 554291-C3
Th, Advanced Cell Diagnostics, Inc Cat No. 317621-C4
Opal 520, AKOYA BIOSCIENCES, FP1487001KT
Opal 690, AKOYA BIOSCIENCES, FP1497001KT

Validation

The Ab validation was done by the suppliers and are verified by published papers.
Key reference for Anti-α-actinin Abcam Ab9465:
Lyu L, Chen J, Wang W, Yan T, Lin J, Gao H, Li H, Lv R, Xu F, Fang L, Chen Y. Scoparone alleviates Ang II-induced pathological myocardial hypertrophy in mice by inhibiting oxidative stress. J Cell Mol Med. 2021 Mar;25(6):3136-3148. doi: 10.1111/jcmm.16304. Epub 2021 Feb 9. PMID: 33560596; PMCID: PMC7957216.
Key reference for Anti-Th Abcam Ab6211:
Song N, Zhu H, Xu R, Liu J, Fang Y, Zhang J, Ding J, Hu G, Lu M. Induced Expression of kir6.2 in A1 Astrocytes Propagates Inflammatory Neurodegeneration via Drp1-dependent Mitochondrial Fission. Front Pharmacol. 2021 Jan 28;11:618992. doi: 10.3389/fphar.2020.618992. PMID: 33584303; PMCID: PMC7876245.
Key reference for Anti-DYKDDDDK Epitope Tag Antibody
Lobb-Rabe M, DeLong K, Salazar RJ et al. Dpr10 and Nocte are required for Drosophila motor axon pathfinding Neural development 2022-10-21 [PMID: 36271407] (IF/IHC)
Key reference for Anti-CFP Polyclonal antibody
Bhattacharya et al., Pharmacological disruption of hepatitis C NS5A protein intra- and intermolecular conformations. J. Gen. Virol., Feb 2014; 95: 363 - 372.
RNAscope probes:
Hcn4, Advanced Cell Diagnostics, Inc Cat No. 421271-C2, validated by the supplier
Bmp10, Advanced Cell Diagnostics, Inc Cat No. 415921, validated by the supplier
Th, Advanced Cell Diagnostics, Inc Cat No. 317621-C4, validated by the supplier
The secondary antibodies are validated by the suppliers and are widely used.

Animals and other research organisms

Policy information about studies involving animals; ARRIVE guidelines recommended for reporting animal research, and Sex and Gender in Research

Laboratory animals

C57BL/899 6J mice are used for genetic mouse model generation.
Information if housing is shown below: 1.1.2 Temperature Rodent rooms should be maintained within a temperature range appropriate for the species and strain, usually 20°C to 24°C for mice, rats, gerbils and hamsters and 15-21°C for guinea pigs. Biocontainment systems, for example IVCs or isolators, should be set to ensure that the temperature that the animal experiences is appropriate, usually within these ranges. Local temperatures among groups of rodents in solid-floored enclosures will often be higher than room temperatures. Even with adequate ventilation the enclosure temperatures may be up to 6o C above room temperature, and this may represent the preferred nesting temperature for the animals as indicated by good breeding performance and absence of indicators of poor welfare. Nesting material/ nest boxes give animals the opportunity to control their own microclimate. Special attention should be paid to the temperatures provided for hairless animals.
1.1.3 Humidity It is advisable that the relative humidity in rodent facilities should be kept at 45 to 65%. Excepted from this principle are gerbils, where it is advisable that the relative humidity be maintained between 35 and 55%.
1.1.4 Lighting It is recommended that light levels within the enclosure are low. The light levels within cages are more important to the welfare of breeding rats, mice and hamsters than the light level in the room. All racks should ideally have shaded tops to reduce the risk of retinal degeneration. This is of particular importance for albino animals. A period of red light at frequencies undetectable to the rodents can be useful during the dark period so that staff can monitor the rodents in their active phase. The importance of light to dark cycles in regulating circadian rhythms and stimulating and synchronising breeding cycles is well documented. A daily cycle of 12:12 is usual. It is advisable that animals, especially when breeding, are given the opportunity to withdraw to shaded areas within the cage, for example by provision of adequate nesting materials.
1.1.5 Noise As rodents are very sensitive to ultrasound, and use it for communication, it is important that this extraneous noise is minimised. Ultrasonic noise (over 20 kHz) produced by many common laboratory fittings, including dripping taps, trolley wheels and computer monitors, can cause abnormal behaviour and breeding cycles. It is advisable to check the acoustic environment over a broad range of frequencies and over extended time periods. Sudden irregular noises create more disturbance in breeding rodents than continuous or predictable sounds. The rodent neonate uses ultrasound production to communicate distress – it is important that extraneous noise is minimised during late pregnancy and early lactation to reduce the risk of mismothering or cannibalism

Wild animals

No wild animals were used for this study

Reporting on sex

both genders were applied

Field-collected samples

No field collected samples were used for study

Ethics oversight

All animal experiments were performed on neonatal or adult mice in accordance with the United Kingdom Animals (Scientific Procedures) Act 1986 and were approved by the University of Oxford Pharmacology ethical committee (approval ref. PPL: PP8557407) and the national guidelines under which the institution operates. The mice used in this study were maintained in a pathogen-free facility at the University of Oxford. Mice were given ad libitum access to food and water.

Note that full information on the approval of the study protocol must also be provided in the manuscript.
